# A practical guide to evaluating sensitivity of literature search strings for systematic reviews using relative recall

**DOI:** 10.1017/rsm.2024.6

**Published:** 2025-03-07

**Authors:** Malgorzata Lagisz, Yefeng Yang, Sarah Young, Shinichi Nakagawa

**Affiliations:** 1 Department of Biological Sciences, University of Alberta, Edmonton, AB, Canada; 2 Evolution & Ecology Research Centre and School of Biological, Earth and Environmental Sciences, University of New South Wales, Sydney, NSW, Australia; 3 Theoretical Sciences Visiting Program, Okinawa Institute of Science and Technology Graduate University, Onna, Japan; 4 Carnegie Mellon University, Pittsburgh, PA, USA

**Keywords:** bibliographic databases, evidence synthesis, information storage, information retrieval, searching, validity

## Abstract

Systematic searches of published literature are a vital component of systematic reviews. When search strings are not “sensitive,” they may miss many relevant studies limiting, or even biasing, the range of evidence available for synthesis. Concerningly, conducting and reporting evaluations (validations) of the sensitivity of the used search strings is rare, according to our survey of published systematic reviews and protocols. Potential reasons may involve a lack of familiarity or inaccessibility of complex sensitivity evaluation approaches. We first clarify the main concepts and principles of search string evaluation. We then present a simple procedure for estimating a relative recall of a search string. It is based on a pre-defined set of “benchmark” publications. The relative recall, that is, the sensitivity of the search string, is the retrieval overlap between the evaluated search string and a search string that captures only the benchmark publications. If there is little overlap (i.e., low recall or sensitivity), the evaluated search string should be improved to ensure that most of the relevant literature can be captured. The presented benchmarking approach can be applied to one or more online databases or search platforms. It is illustrated by five accessible, hands-on tutorials for commonly used online literature sources. Overall, our work provides an assessment of the current state of search string evaluations in published systematic reviews and protocols. It also paves the way to improve evaluation and reporting practices to make evidence synthesis more transparent and robust.

## Highlights

### What is already known

- Designing and optimizing search strategies is one of the key steps in systematic reviews and meta-analyses.
- Objectively assessing search performance is difficult when the whole body of relevant evidence is unknown.
- A relative recall approach (benchmarking) is based on testing the ability to capture a pre-defined set of relevant studies.
- There currently needs to be more practical guidance on how to conduct objective evaluations of search strings using a benchmarking approach.

### What is new

- We show that search string evaluations are almost never reported.
- Our tutorial introduces a simple and practical benchmarking workflow using a relative recall approach for search string sensitivity evaluation.
- The proposed workflow can be easily implemented exclusively using online user search interfaces of the commonly used online databases.
- We provide work examples of how to conduct benchmarking for five online search platforms and databases.

### Potential impact for RSM readers outside the authors’ field

- Descriptions of search string evaluations should be provided in the documentation of systematic reviews, and in our tutorial, we provide practical recommendations on how to do this.
- Our methodological guidance promotes and enables objective search string evaluations, which are easy, quick, and relevant to commonly used database platforms.
- Objective search string evaluations not only offer an opportunity to refine search strategies but they also can be used as evidence that the search captures a sufficiently complete and representative range of studies or can provide information about limitations of the search.
- Objective evaluation of search strings using benchmarking can be a simple but powerful tool for ensuring research evidence is as complete and unbiased as possible.

## 1 Introduction

### 1.1 Systematic searching for evidence using search strings

**Figure 1:**
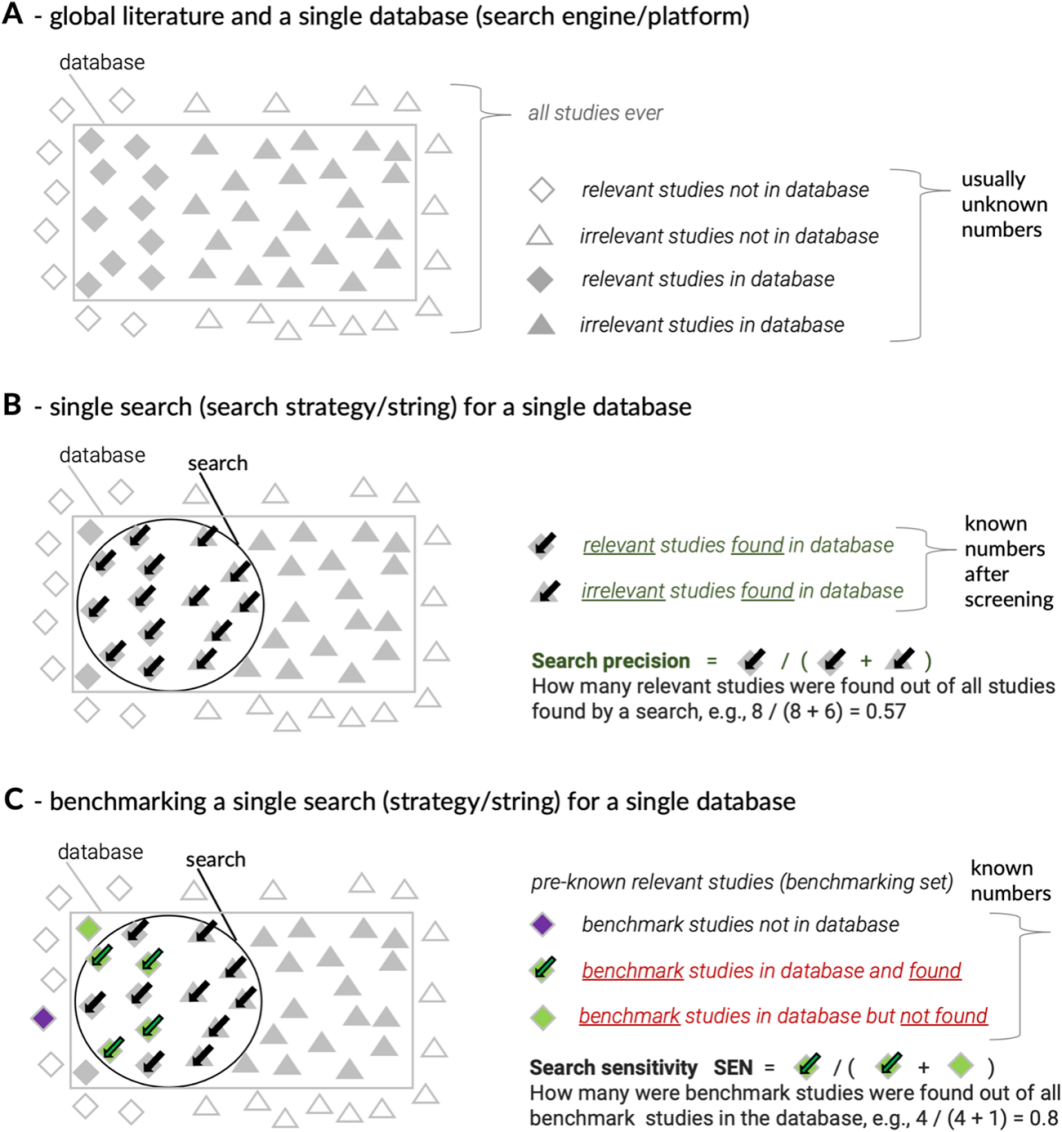
Conceptual representation of the body of evidence, database coverage, search string capture, and search string evaluation. (a) The vast body of evidence contains a certain unknown number of relevant studies but only some are indexed in a given database. The true number of relevant bibliographic records in a given database is unknown. (b) The subset of records retrieved by a database search contains an unknown number of relevant and irrelevant bibliographic records until all relevant records are assessed for relevance (screened). Then, search precision can be calculated as the proportion of relevant records. Search precision and the total number of captured relevant records can also be estimated by screening a random sub-sample of records from all search hits. (c) Search evaluation (validation or benchmarking) can be performed using a predefined test set of relevant studies (benchmarking set). Search sensitivity is calculated as a proportion (or percentage) of indexed benchmark studies (bibliographic records) that are found by a search string.

Systematic reviews usually aim to identify all or a majority of relevant and representative evidence.^1^ Here, by evidence, we mean published academic research. Most published academic research is cataloged in online databases, which typically collate vast numbers of bibliographic records of publications. However, there is no single database or search platform that collates all available evidence (Figure 1a). To increase the comprehensiveness of located evidence, systematic reviewers use more than one database or search platform.^2^ ^,^ ^3^ This increases the reviewers’ workload but also exposes them to different database user interfaces and search functionalities. Still, most of the online databases of academic literature work by interpreting user-provided search strings (Figure 1b). Search strings are logical (Boolean) expressions built from combinations of search terms (words and phrases) reflecting the focus of the research question behind the literature search. Search strings are interpreted by database algorithms as data filters helping to retrieve relevant bibliographic records. When researchers use inadequate search strings, they may miss important evidence or end up with a set of records that is not representative of the whole body of evidence. Non-representative samples may exacerbate publication bias or introduce other biases affecting the conclusions of a systematic review.^4^ ^,^ ^5^ Thus, getting search strings right is one of the critical steps in the evidence search workflow, but there is limited guidance on testing search strings. This paper presents a practical approach to evaluating search strings during their development.

### 1.2 Search string development theory

Developing optimal search strings is a balancing act between sensitivity and precision.^6^ The basic premise is to find a combination of search terms that will retrieve most of the relevant records (high sensitivity) but not too many non-relevant records (high precision) so that the total number of records to be screened is manageable. This is a hard balance to strike for two reasons. First, sensitive searches tend not to be precise and return many irrelevant records because they involve using many search terms with broad meanings. Second, precise searches are usually not sensitive and miss relevant records because using only a few precise search terms excludes studies vaguely described by complementary terms with broader meanings.^7^ To find the right balance between sensitivity and precision, researchers usually follow an iterative process, trying out different combinations of search terms.^4^ Usually, searches with a high number of hits (retrieved records) have low precision (the ratio between captured relevant papers and the total number of retrieved papers). Precision can be easily estimated by screening a random sample of records retrieved by a search (e.g., if out of 100 records 5 are relevant, then precision is 0.05, or 5%). In contrast, to estimate sensitivity one needs to also know the number of missed relevant papers—which is difficult as they are usually unknown. The process of estimating the sensitivity of a search string is called “evaluation” (or “validation”; see^8^ and^9^ for a discussion on terminology).

### 1.3 Search sensitivity evaluations

There are two main types of search sensitivity evaluation: “conceptual” and “objective,” both of which should be implemented in any evidence synthesis review.^10^ The first one is based on peer review by an expert, where the expert usually is an experienced information specialist. Having such a specialist involved in the process is highly recommended in general, and often explicitly required.^11^ ^,^ ^12^ Expert evaluation can tell us if a search string development followed the best practice, which is usually a set of general rules and recommendations, but is subjective. Objective evaluation is based on explicit testing of a search string performance, where sensitivity is estimated quantitatively as the proportion of the relevant articles being captured. However, there are a few challenges that need to be considered when thinking about implementing search sensitivity evaluations.

### 1.4 Challenges for wide adoption and implementation

There are four types of challenges (and barriers) to the adoption and implementation of objective search sensitivity evaluations. The first challenge stems from the mismatch between the theory of performing such evaluations (as presented above) and its practical implementation: calculating sensitivity, by definition, requires performing a comparison against the perfect retrieval of all relevant evidence, which is usually unknown.^3^ ^,^ ^13^ ^,^ ^14^ In practice, it is often performed using a known subset of all relevant evidence such as a priori collection of relevant studies (a “benchmarking set” in Figure 1c; also known as “gold standard,” “golden-standard set,” “gold studies”, “validation set,” “test set,” “comparator set,” “known set,” “reference standard,” “reference set,” “reference standard records,” “seed documents,” “seed studies”). Under this approach, search sensitivity may be called “recall ratio”^15^ or “relative recall.”^16^ This indicates a second challenge - the confusion stemming from the diverse and inconsistent terminology (e.g., on top of the examples mentioned above, the interchangeable use of “sensitivity” and “recall,” while doing “evaluation” or “validation” or “testing” or “benchmarking”). Inconsistent terminology makes finding relevant practical advice more difficult. The third challenge is closely related—the lack of clear guidance on how to perform search string evaluation in practice. Relevant and specific advice is scattered across disciplines and constantly evolving. Many of the proposed new tools and techniques are accessible only to users with expertise in computing and information science because they use text mining (e.g., Hausner et al.^10^), language modeling (e.g., Scells et al.^17^) or custom machine learning algorithms (e.g., Scells and Zuccon^18^), and general lack of formal evaluations of the effectiveness of various approaches.^19^ The final challenge is related to implementing sensitivity evaluations across many databases or search engines. This is because comparing the composition of large sets of records from many disparate searches and sources is time-consuming and error-prone. Rather than doing this manually, researchers need to use efficient workflows for detecting overlaps and differences between retrieved sets of bibliographic records and their benchmarking set. Given these four challenges, objective string sensitivity evaluations are likely rare in published systematic reviews and systematic review protocols, particularly when information specialists are not involved.

### 1.5 Aims

This article aims to address the four challenges to the practical implementation of objective search string evaluations. We achieve this by giving an overview of current reporting practices related to search string evaluations and providing practical recommendations with workflows for implementing search sensitivity evaluations via relative recall (benchmarking). Specifically, this work consists of two parts:

1. Evaluating reported practices in search string sensitivity evaluation using two surveys of recently published literature across disciplines. Here, we investigate whether search string development and evaluation processes have been reported, the availability of final search strings for each search source, the main search sources used, and the involvement of an information specialist.
2. Based on the assessments of reported current practices, we provide practical recommendations for conducting search string sensitivity evaluations. We focus on the relative recall (benchmarking) approach because of its simplicity and efficiency, which makes it easy to implement even for researchers who are not information specialists. With such researchers in mind, we include a series of hands-on examples using five online academic literature databases.

## 2 Survey

We conducted two separate literature surveys using representative samples of recent systematic reviews from two sources to assess current practice. We registered a survey protocol on OSF (https://osf.io/wq6dh).

### 2.1 Methods

Our first survey aimed to reveal the state of practice in the “general population” of systematic reviews published across disciplines, which are considered to be generally of low quality.^20^ ^,^ ^21^ Here, we used a representative cross-disciplinary sample of 100 recent systematic reviews (all published in 2022) to assess the current frequency of reporting search string evaluation procedures, and how they are conducted. Next, we also surveyed a sample of 100 Cochrane Reviews protocols (also published in 2022), to elucidate practices implemented in systematic reviews that are recognized as a gold standard for their rigor.^18^ We focused on protocols because the completed Cochrane Reviews usually only present their final search methods. We would expect to find details about how the search strategy was developed and evaluated, and any justifications or rationale for the search strategy approach, in the protocol. Moreover, a brief sampling of published Cochrane reviews showed no information about search strategy development. In Supplementary File 2, we provide the description of collating representative samples for each survey, extracted variables, and data validation procedure.

### 2.2 Results and discussion

#### 2.2.1 Cross-disciplinary sample of systematic reviews

**Figure 2:**
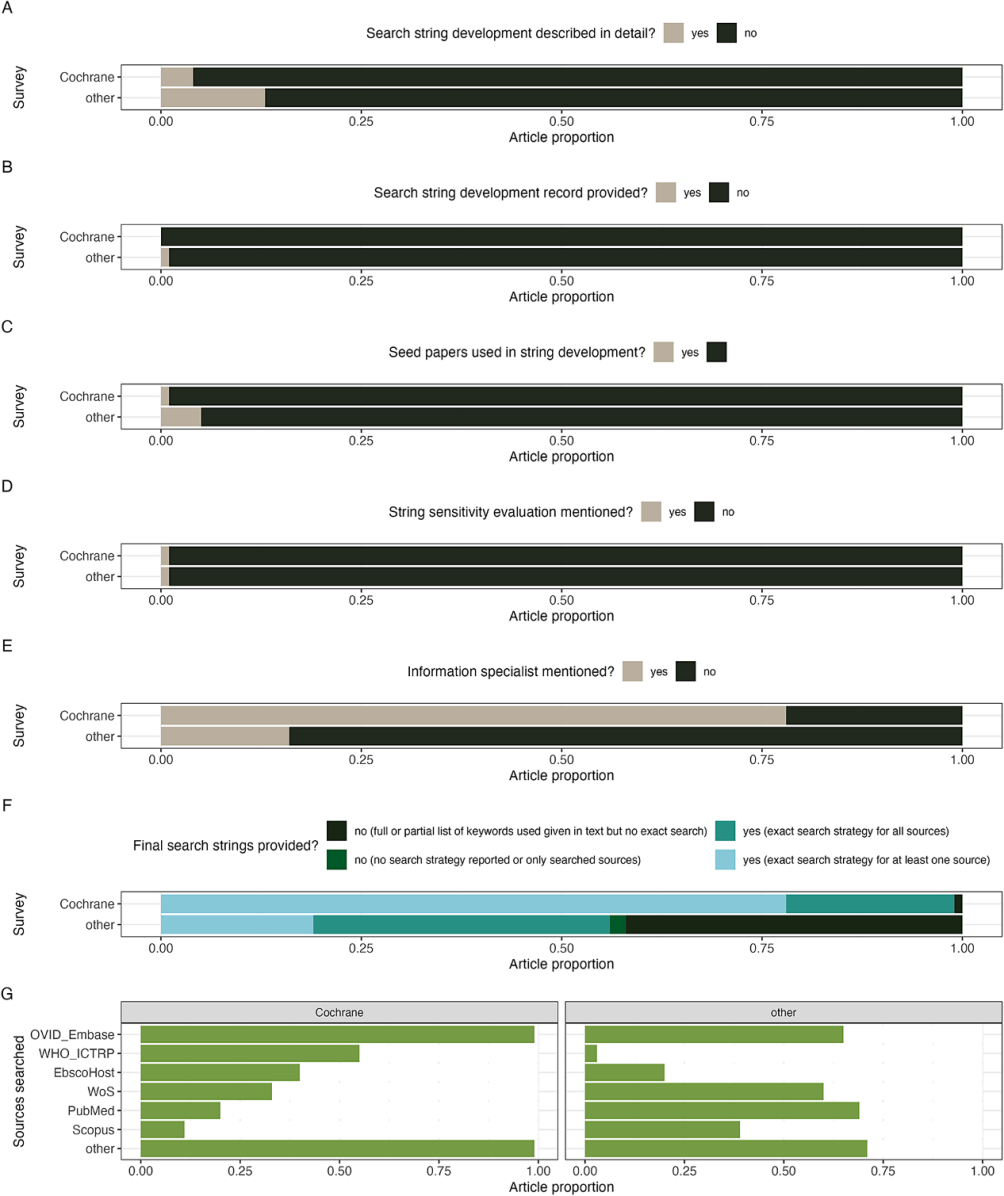
Results of two surveys assessing reporting of search string development and evaluation in two types of representative literature samples: a sample of 100 published Cochrane protocols (Cochrane) and a cross-disciplinary sample of systematic reviews (other), from 2022. Comparison between the two literature samples (Cochrane vs. other) for: (a) frequencies of providing a description of the process used for developing the final search string, (b) frequencies of providing a record of different search string variants tried during string development, (c) frequencies of reviewers noting using a set of known relevant studies to discover relevant terms for the search string, (d) frequencies of providing a mention of performing search string evaluation (validation, benchmarking, etc.), (e) frequencies of involving an information specialist in planning or performing the systematic review, (f) frequencies of providing the final search strings and in how much detail. (g) Bar plot showing the most common search sources (databases, search platforms, or engines) that were used (or planned) for performing searches (most systematic reviews used more than one search source so that proportions do not add to 1) for the two literature samples. All detailed results and our analysis code are available in Supplementary File 2 and at https://github.com/mlagisz/method_benchmarking_survey.

In our general sample of 100 systematic reviews, 13 (13%) described their approach to search string development. Only one provided an explicit record of testing variants of their search string (i.e., a search string development record; Figure 2). Five reviews acknowledged harvesting initial search terms from a set of known relevant papers (seed papers). One review mentioned search string validation “with the 105 journal articles already included in the previous version of the ASDB.”^22^ The involvement of an information specialist was mentioned in 16% of the sampled reviews and associated with the reporting of one or all the final search strings (*p* = 0.006, Odds Ratio = 6.88, 95% CI = 1.44 to 66.24). Reviews without or with only vague information on the final search strings (no search strategy reported, or only searched sources, or full or partial list of keywords used but no exact search string) were common (44%). At least one exact search string was reported in 19%, and all final search strings were reported in 37% of the cross-disciplinary systematic reviews. The three most used databases or database platforms were: PubMed, EBSCOhost, and Web of Science.

#### 2.2.1 Cochrane sample of published protocols

In our sample of 100 Cochrane systematic review protocols, 4 (4%) described their approach to search string development and none provided an explicit search string development record (Figure 2). One review acknowledged using seed papers for harvesting initial search terms.^23^ One review benchmarked their Embase search “against a set of known studies for each of the five self-report instruments (index tests).”^24^ The involvement of an information specialist was mentioned in 78% of the sampled review protocols and was not associated with the reporting of one or all the final search strings (*p* = 1.00, Odds Ratio = 0.00, 95% CI = 0.00 to 137.98). Protocols without or with only vague information on the final search strings were rare (1%). At least one exact search string was reported in 78%, and all final search strings were reported in 21% of the published Cochrane protocols of systematic reviews. The three most used search sources were: Ovid Embase, EBSCOHost, and the WHO International Clinical Trials Registry Platform.

#### 2.2.3 Comparison and discussion of survey results

Overall, we found that most published systematic reviews and published Cochrane protocols do not describe how search strings were developed and whether they were evaluated for sensitivity, indicating these are universal issues. Cochrane reviews protocols are more likely to have an information specialist involved (Fisher’s exact test *p* < 0.001, odds ratio = 18.25, 95% confidence interval (CI) = 8.65 to 40.65) and to present an exact final search string for at least one searched literature source (*p* < 0.001, odds ratio = 7.22, 95% CI = 8.65 to 31.95). Both general systematic reviews and published Cochrane protocols tended to use several sources for their searches (median = 5). However, the distributions of the most used search sources differed between general and Cochrane-based surveys. Still, Ovid Embase, PubMed, Web of Science, and Scopus, each appeared in at least 40% of the cross-disciplinary systematic reviews.

Our finding of limited reporting of search sensitivity evaluations could be potentially explained by the observed poor reporting of the search development process in both surveys. The ubiquitous involvement of information specialists in Cochrane reviews appears to be linked only with more exact reporting of the final search strings, but not with mentions of performing objective evaluations of search strategies. This lack of reporting on search strategy development procedures and evaluation could also be due to an absence of this requirement in Cochrane’s Methodological Expectations of Cochrane Intervention Reviews (MECIR^25^) and only the recent addition of the search string evaluation recommendations to PRISMA2020 reporting guideline.^26^

## 3 Tutorial

### 3.1 Search sensitivity evaluations—key principles

Conducting search sensitivity evaluations is a critical part of search string development, but as shown by our surveys, it is not regularly reported. Conducting and disclosing evaluations of search string sensitivity is recommended in general advice articles (e.g.,^27^ ^,^ ^28^), reporting checklists (ROSES-SR,^29^ PRISMA 2020^26^), and a registration template.^30^ Furthermore, this critical procedure needs to be considered and conducted early on—when planning the search strategy. The planning of search evaluation requires considering early how to objectively evaluate the string performance, that is, answering the question “what set of studies should we compare our search string results to?”

As noted in the Introduction, we cannot know the true number of all existing relevant studies on a given topic, so we cannot use this number to evaluate our search success. Usually, we also do not even know the true number of relevant studies on a given topic in a given online database of literature (Figure 1a,b^31^). This means that when performing the evaluation of search sensitivity (recall), we need to rely on some other point of reference (relative recall), rather than trying to get the true value (absolute recall). This situation is equivalent to a typical problem with scientific research where we want to know the true value for something of interest for a whole population, but for practical reasons, we can only take measurements from a representative sample. We then use these measurements to get an estimate of the true population value. The same approach could be applied to search sensitivity evaluation.

For search sensitivity estimation, we can use a representative (non-exhaustive) set of known relevant studies (Figure 1c^1^). We call them a “benchmarking set,” “benchmark studies,” or “benchmarks” for short (for other equivalent terms used in this context, see the Introduction section). Benchmark studies must be collected before any targeted database searches are performed (i.e., before trying to develop a search string). It is critical that they are representative of the relevant literature. They must come from many diverse sources, such as earlier narrative or systematic reviews, personal collections or recommendations, online searches based on similarity or citation tracking, and they should be peer-reviewed by experts.^10^ ^,^ ^28^ The exact approach to creating a benchmarking set can be customized to each systematic review topic. For example, publications describing the development of search filters for study methodologies use different combinations of hand searches, bibliography, and database searches, ideally across multiple journals and years.^32^ However, no studies have examined the effect of different approaches on the representativeness of the benchmarking set. In contrast, search strategies can be validated via peer review, which can be performed informally or using a structured tool, such as the PRESS 2015 Evidence-Based Checklist.^33^ ^,^ ^34^

As noted briefly in the Introduction, search sources (databases) differ in their coverage of the evidence base.^3^ ^,^ ^35^ For example, PubMed mostly collates health-related studies, but Scopus has a much broader cross-disciplinary coverage with fewer health-related records. Thus, some of our benchmark studies may be absent in a given database. This needs to be accounted for when benchmarking using a single database, by removing the absent benchmark studies from the evaluation for that database. Benchmarking evaluations can be expanded across many databases by combining the results of evaluations of each database or by combining retrieved records before performing the evaluation. In addition, the absence of benchmark studies from a given source can be valuable information that informs source selection and may indicate that other databases or grey literature sources should be considered to minimize search bias and improve comprehensiveness.

Finally, databases that allow long and flexible search strings are easy to evaluate efficiently because we can use this functionality to check the overlaps of sets of records. Additionally, being able to search by study ID numbers (e.g., DOI, or some other unique study identifiers) is essential. It prevents ambiguity in the retrieval of benchmark studies and allows the construction of a compact search string for the whole benchmarking set. This is achieved by piecing together benchmark ID numbers using the “OR” Boolean operator. This benchmarking search string can then be combined, using the “AND” operator, with any other search string, revealing the overlap. The extent of the overlap (the count of overlapping records) is then divided by the size of the relevant benchmarking set to estimate the sensitivity (relative recall; SEN in Figure 1c).

Next, we break this process down further into even smaller and clearer steps with more details. In our Supporting Information, we also provide specific examples of an actual benchmarking process conducted for five online databases. We note that search string construction and development in a broad sense is beyond the scope of this tutorial, we thus refer the interested readers to other resources (e.g.,^1^ ^,^ ^4^).

### 3.2 Search sensitivity evaluations—steps

**Figure 3:**
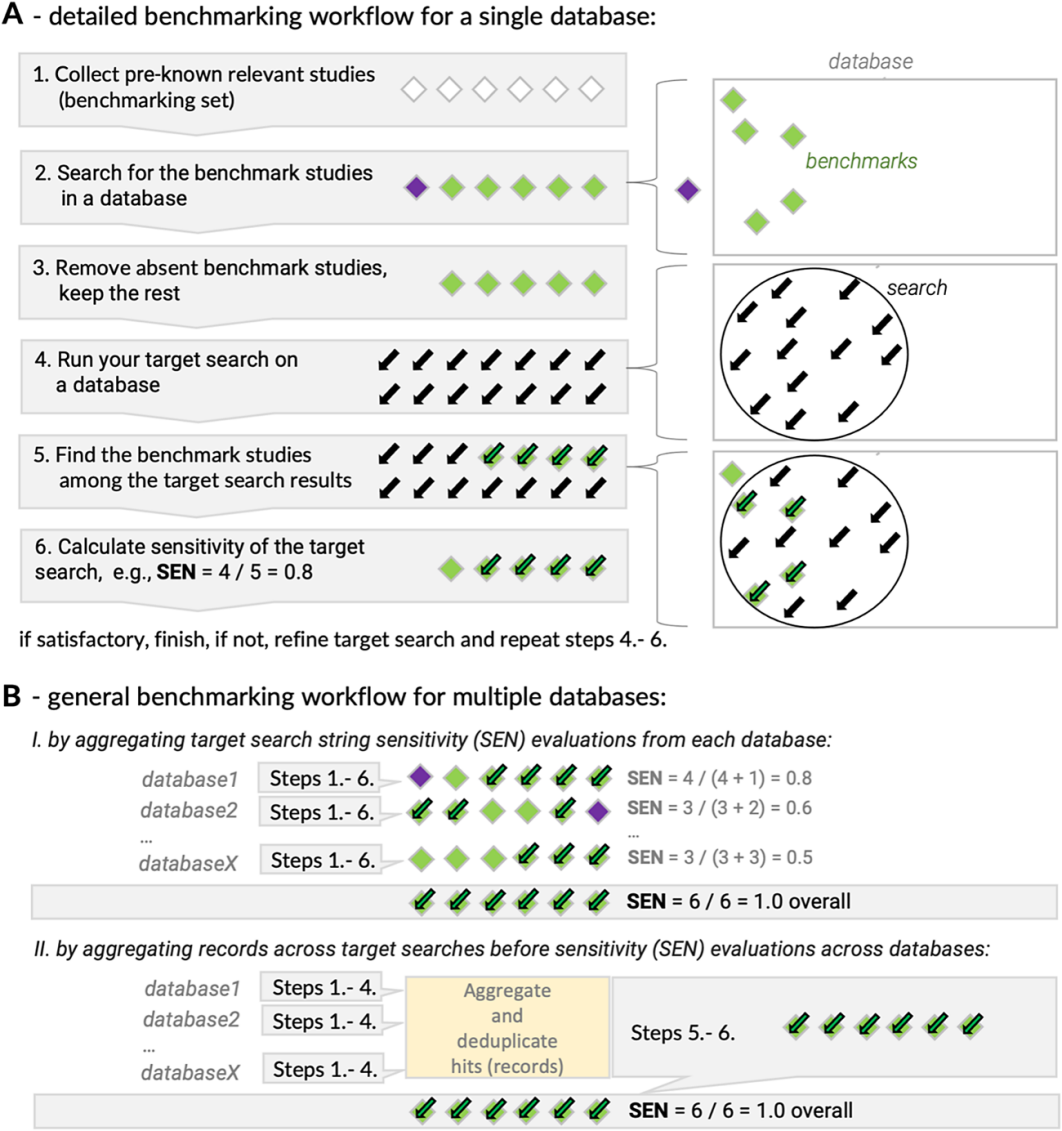
Practical implementations of search sensitivity evaluation (benchmarking)s. (a) A simplified benchmarking workflow for a single database (search source). (b) two alternative approaches to working with multiple databases (search sources): I—searches are evaluated separately for each database before being aggregated into an overall estimate; II—records (hits) retrieved by search strings in all databases are pooled together before evaluation is performed. “Steps” refer to steps 1–6 shown in panel A.

Figure 3 shows the main steps of the search sensitivity evaluation for a single online database (search source). Below we provide more details and additional advice on how to adjust this workflow if evaluating searches across multiple databases:

1. Collect pre-known relevant studies (benchmarking set):
  - Define the scope of your systematic review (or any systematic-like review using a systematic search approach) and its inclusion and exclusion criteria.
  - Select the search sources to be used in your systematic review.
  - Decide if search evaluation will be performed for one or more search sources, and which ones.
  - Gather a set of potential benchmark studies from diverse sources. Avoid using the databases you are planning to use as your systematic review search sources.
2. Search for the benchmark studies in a database you are evaluating:
  - Create a benchmarking search string from all ID numbers (e.g., DOI) of the benchmark studies, using the “OR” Boolean operator.
  - If a benchmark study is not found by its ID, it is either because of the true absence of the study record or incorrect/missing ID. Thus, for each incorrect/missing benchmark study run a search using its title or other identifying details (e.g., author, year). If found, check if the ID is correct and fix/replace the ID if needed, then search again by ID only. Pay attention to other potential issues, such as duplicated records, or single ID representing collections of works (e.g., conference abstracts book). Continue checking and refining until you have a benchmark search string that retrieves all benchmark studies present in each database.
  - Optional: Repeat for each database that will be used in search string evaluations.
3. Remove absent benchmark studies, and keep the rest (i.e., customize your benchmarking set for each database so that you do not count absent benchmark studies in search string sensitivity calculations for that database).
  - You can do this by simply removing IDs of the missing benchmark studies from a search string for a given database. This way you will have a clean benchmark search string with the IDs matching all benchmark studies present in a given database, which will make your search refinement and calculations easier.
  - Alternatively, you can just note which and how many benchmark studies are missing from a given database, and later adjust your search string sensitivity calculations accordingly.
  - If relevant, set aside any benchmark studies that are absent from all of the databases you had planned to search. You can come back to these later to determine where they can be found (e.g., a grey literature source, an unindexed journal) and to determine if additional sources should be searched for your review.
4. Run your target search string on a database.
  - Typically, your target search string is a string composed by combining review scope-related terms (e.g., keywords, fixed expressions, controlled vocabulary, etc.) using Boolean (AND, OR) or other operators and field filters (e.g., which part of the bibliographic record to search, and any additional search limitations, like publication years or subject areas).
  - The number of returned records (“hits”) can vary vastly and you should keep track of it for later target search string refinement.
5. Find the benchmark studies among the target search results.
  - This step tests the overlap between records retrieved by the target search string and the benchmark set. Here we can simply combine the two strings. For example, if StringA is a target search string to be evaluated for recall, and if StringB retrieves bibliographic records for all benchmark studies by using their ID numbers, then running a combined search sting in a format “(StringA) AND (StringB)” will retrieve the records that overlap between the two. You could also have a look at which benchmark papers were found.
  - Optional: If some benchmark records are missing, you can sometimes use a “NOT” operator to see which ones are missing (i.e. “(StringB) NOT (StringA)”).
6. Calculate the sensitivity of the target search string.
  - The number of overlapping records between the two search strings (target and benchmarking) is the number of benchmark studies found by the evaluated target string (StringA). Thus, this number, divided by the total number of records retrieved by the benchmarking string (StringB) is the estimate of your search sensitivity (SEN or relative recall).
  - Optional: You can iteratively modify your target search string (StringA). At every iteration, it is very easy to re-evaluate the new target StringA against the benchmarking StringB using the same method as above (combining the strings). When modifying your search string, you can start by reading through the titles and abstracts of these missed studies. Determine why the study was missed by your current search strategy. What terms are missing from your search string? If reasonable, add the missing search terms to your search (e.g., add terms that are synonyms of concepts already included in your search, expand proximity windows, adjust stemming, etc.). If there is no reasonable way to adjust the search to capture the study, make a note of this as a potential limitation of your search strategy.
  - Recommended: Keep a good record of the search development and testing process (e.g., in a table, see our example tables in Supplementary File 1), so you can document it transparently in your systematic review or protocol.

### 3.3 Search sensitivity evaluations—limitations

There are five limitations to the proposed benchmarking workflow. First, it relies on relative recalls, rather than absolute recall of all existing relevant evidence. We currently have no objective way to judge whether the benchmark papers are representative or whether a given relative recall is a good estimate of the absolute recall. Again, we would need to know all available relevant evidence to judge this first. In such a case, we should be able to assess absolute recall directly (and it would be only possible in rare cases where all relevant studies are already known). This, in turn, makes conducting a systematic search for them unnecessary, rendering this limitation irrelevant.

Second, evaluation workflow using relative recall requires additional effort in assembling the set of benchmark studies early on, ideally having them reviewed by an expert, and checking if they are indexed in each of the evaluated databases. This is not necessarily a major obstacle, as it can be a part of the initial scoping process of a systematic review,^4^ and checking database indexing is usually quick using the search function.

Third, the estimates of search string sensitivity are not precise for small benchmarking sets. For example, evaluating search strings against two benchmark studies will not be very informative. The optimal number of studies in a benchmarking set has not been established yet.^32^ Publications describing the development of search filters for study methodologies used between 15 and 1,347 studies in their benchmarking sets.^32^ Benchmarking sets may introduce bias in the search strategy if they are not representative of the whole range of available evidence. However, sometimes finding many benchmark studies may not be easy. This could be simply because only a few relevant studies exist on the given topic, or they are not indexed by the literature databases used. In general, the more benchmarking studies you have, the more robust your evaluation.

Fourth, the evaluated databases need to allow the use of long and flexible search strings with nested Boolean operators and search fields for document ID codes (e.g., DOI). Workarounds with exporting sets of records and manually (or via programming code) checking the overlaps are possible. However, such workarounds would be more time-consuming and less accessible to many systematic reviewers in comparison to performing all the operations directly within the search source (an online database user interface). The same limitation applies when combining records downloaded from multiple databases before performing sensitivity evaluation.

Fifth, there is no one universal sensitivity value that should be used as a threshold when refining search strings and strategies. While some guidelines for systematic reviews insist on full comprehensiveness (100% = finding all; e.g.,^36^ ^,^ ^37^), it is also accepted that it is more realistic to aim for the majority of the relevant evidence (e.g.,^1^ ^,^ ^5^ ^,^ ^28^). However, the question is open on where and how the search strategy refinement should stop and needs to be answered by the review team on a case-by-case basis.^38^

## 4 Conclusions and recommendations

### 4.1 Search sensitivity evaluations—current practices

Our two surveys of recent literature show that reporting of objective search string sensitivity evaluations is almost absent from published systematic reviews and their protocols. Critically, our surveys represent both a cross-disciplinary sample of systematic reviews and a sample of published Cochrane protocols, but we found little difference in reporting of search string development and evaluation between the two samples. This finding indicates that the reporting requirements for the search string development process, as well as recommendations on performing objective evaluations of a search strategy, are usually ignored. How can we encourage search string evaluations and improve their reporting?

### 4.2 Recommendations for conducting search sensitivity evaluations

We provide seven methodological recommendations for conducting objective search string evaluations:

1. Evaluate search strings for at least one of the main search sources using a pre-defined set of benchmarking papers.
2. Create your set of benchmarking papers by combining different approaches, such as hand searches, bibliographic database searches, and personal recommendations.
3. Use a search source that makes benchmarking easy by combining search strings.
4. Follow the hands-on examples provided in Supplementary File 1, which are accessible to all researchers and information professionals.
5. Use search string evaluation as an opportunity to refine your search strings to balance their sensitivity and precision, bringing the total number of bibliographic records to be screened to an acceptable range by focusing on the review question.
6. Use benchmarking studies that are absent from search sources to identify additional sources to search in your review or to identify potential limitations in your search strategy.
7. If possible, get an information specialist involved in search string development and credit them for their contributions in acknowledgments or via authorship, as appropriate.

### 4.2 Recommendations for reporting search sensitivity evaluations

Search string development and evaluation need to be not only conducted but also transparently reported in the protocol or a systematic review report.^39^ We provide five recommendations for achieving this:

1. Provide a list of references of the studies used as a benchmarking set and describe how the set was collated.
2. Report exact search strings used to retrieve benchmark studies for each search source alongside the exact target search strings being benchmarked and search sensitivity estimates.
3. Treat benchmarking as an integral part of the search string development process and report them in detail together, including dates, filters, and comments on how decisions are made on refining the search strings during the search refinement iterations.
4. Report any potential limitations of your search strategy that were identified in this process. What benchmark studies were not captured by your final search string and why? Based on these missing studies, what can you say about the potential for other similar missing but unknown studies from your search?
5. Report on biases that may be present in your benchmark set due to the approach used to create it, e.g., database coverage, publication years, journals, or language limitations.

Finally, following the above recommendations will help you build high-quality search strategies and improve the transparency of the development process of search strategies. Further, it will signal the robustness and validity of your search strategy to the reviewers and readers of your systematic review or meta-analysis. Unfortunately, few reporting checklists require objective search string validations or even documentation of the search string development process (note that PRISMA-S item 14 currently only requires to “Describe any search peer review process”^40^). Thus, we also recommend adding the use of an objective search sensitivity evaluation approach to reporting checklists. More research is needed on the optimal development and use of benchmarking sets for evaluating systematic searches. Hopefully, our work will contribute to the wider adoption of this critical procedure for making systematic searches more transparent and reliable.

## Supporting information

Lagisz et al. supplementary material Lagisz et al. supplementary material

## Data Availability

Project GitHub repository with all data and code can be found at https://github.com/mlagisz/method_benchmarking_survey and is archived on Zenodo at https://doi.org/10.5281/zenodo.14017730.

## Supplementary material

To view supplementary material for this article, please visit http://doi.org/10.1017/rsm.2024.6.
