# Supplementary material for: A practical guide to evaluating sensitivity of literature search strings for systematic reviews using relative recall
Source: Res Synth Methods. 2025 Mar 7;16(1):1–14. doi: 10.1017/rsm.2024.6 (PMC12621535; doi:10.1017/rsm.2024.6)
Supplement: Lagisz et al. supplementary material [file S1759287924000061sup001.zip › Supplementary File 2.html]

Supplementary Information - Survey methods and results


# Supplementary Information - Survey methods and results

#### Malgorzata Lagisz

#### 20/02/2024

- Project set
  up
- Literature survey methods
  - Survey
    protocol
  - Deviations from survey
    protocol
  - Survey aims
  - Survey methods
    - Survey1 -
      workflow
    - Survey2 -
      workflow
    - Data
      extraction
    - Data
      cross-checking
    - Data
      analyses
- Literature survey results
  - Load and
    check data
  - List of
    extracted papers for Survey1 and Survey2
    - Survey
      1 - cross-disciplinary sample of 100 systematic reviews
    - Survey 2 -
      sample of 100 Cochrane Reviews protocols
  - Process
    and plot extracted data for each key extracted vairiable
    - String\_development\_described
    - String\_development\_record\_provided
    - String\_development\_seed\_papers\_used
    - String\_sensitivity\_evaluation\_mentioned
    - Information\_specialist\_involved
    - Final\_string\_provided
    - Search\_sources
  - Combined
    figure
  - Statitical
    tests
  - Session Info

This document contains supplementary methods, results and code
for:

“A practical guide to evaluating sensitivity of literature search
strings for systematic reviews using relative recall”

by

Malgorzata Lagisz, Yefeng Yang, Sarah Young, Shinichi Nakagawa.

NOTE: project GitHub repository with all data and code can be found
at https://github.com/mlagisz/method\_benchmarking\_survey
(it will be archived to Zenodo when manuscript is accepted for
publication).

# Project set up

Loading packages and general settings.

```
#set global code chunk parameters for knitting:  
knitr::opts_chunk$set(echo = TRUE)

#load libraries (install if absent):  
pacman::p_load(tidyverse, #various tools
               here, #easy file paths
                DT, #data tables
                knitr, #document knitting
                wacolors, #color-blind friendly color palettes
                patchwork
                # ggimage, #adding images
                # ggcharts, #plots functionality
                # scales, #plots extra
                # readxl, #loading Excel
                # forcats, #category wrangling
                # ggupset, #upset plots
                # patchwork, #plot assembly
                # stringr, #text wrangling
                # ggbeeswarm, #beeswarm plots
                # tidytext, #text processing
                # stopwords, #text processing
                # tokenizers, #text processing
                # ggpatterns, #patterning for plots
                # lme4, #linear models
                # sjPlot
               )

## may need:
# install.packages("remotes")
# remotes::install_github("coolbutuseless/ggpattern")
#library(ggpattern)

## set a global plotting theme with background transparency:   
theme_set(theme_classic(base_size = 14) + 
            theme(panel.background = element_blank(), 
                  plot.background = element_blank(), 
                  legend.background = element_blank(), 
                  text = element_text(colour = "white")))
```

# Literature survey methods

## Survey protocol

Before conducting the survey (before data collection) we registered
our project protocol.  
The protocol is available on OSF at https://osf.io/wq6dh.

## Deviations from survey protocol

We did not deviate from our survey protocol except aggregating search
sources with frequency less than 10% into “other” category in the plots
showing our results.

## Survey aims

To evaluate current practices in search string
validation/evaluation/testing/benchmarking across disciplines.

## Survey methods

We conducted two separate literature surveys using two representative
surveys of 100 samples from recent systematic reviews and protocols,
respectively.  
Survey1 covered systematic reviews published across journals and
disciplines and Survey 2 focused on systematic reviews protocols
published in Cochrane Reviews. Due to differences among disciplines in
the degree of adoption of published protocols we did not use protocols
in Survey1. In contrast, Cochrane Reviews have an established standard
for publishing protocols and they are all related to medical sciences.
Also, the search strategy for Cochrane reviews is usually developed at
the protocol stage and is expected to be described in detail in the
protocol with the final report referring to the details in the published
protocol.

### Survey1 - workflow

1. Collecting a sample of general-population systematic reviews
   published in 2022 using Scopus database search string TITLE (
   systematic\* W/3 review\* ) AND ( LIMIT-TO ( PUBYEAR , 2022 ) ). Over
   45,000 records were available for sampling.
2. Drawing a random sample of 100 records published in 2022.
3. Articles without full text available, articles in languages other
   than English, and articles that were not systematic reviews were
   excluded. We then iteratively substituted excluded articles with another
   randomly drawn article with full text available, until we had a sample
   of 100 eligible systematic reviews articles for assessment.
4. Performing data extraction and coding using pre-piloted GoogleForm,
   as described below.
5. Summarizing the extracted data.

### Survey2 - workflow

1. Collecting a sample of systematic reviews protocols from Cochrane
   Reviews protocols published in 2022 Issues 1-12. Approximately 220
   document records were available for sampling.
2. Drawing a random sample of 100 Cochrane Reviews protocols published
   in 2022 (excluding sub-categories of Prototypes, Overviews, Methodology,
   Rapid Reviews, etc., which are not representative of a typical
   systematic review protocol) and checking for full text
   availability.
3. Articles without full text available were iteratively substituted
   with another randomly drawn article from the search, until we have a
   sample of 100 eligible articles with full text available for
   assessment.
4. Performing data extraction and coding using pre-piloted GoogleForm,
   as described below (same as for Survey1).
5. Summarizing the extracted data.

### Data extraction

For each article, ML manually extracted:

1. Information on article identity: DOI, title, first author name,
   publication journal (full reference will be also archived
   separately).
2. Information on search sources: literature sources (e.g.online
   databases/platforms) used for the systematic review following 28 most
   commonly used as listed in Gusenbauer, M, Haddaway, NR. (Which academic
   search systems are suitable for systematic reviews or meta-analyses?
   Evaluating retrieval qualities of Google Scholar, PubMed, and 26 other
   resources. Res Syn Meth. 2020; 11: 181–217;doi:10.1002/jrsm.1378)
   [checkboxes to code the most common 28 search platforms/databases and an
   option to add and describe other sources].
3. Information on search strategy reporting, development, and search
   string sensitivity assessment/validation:

   - whether any description is provided of search string
     development(i.e. providing a description of how the final search string
     was arrived at by describing term harvesting techniques, iteration
     methods, testing strategies, etc. for at least one database; if none or
     only final search strings are reported select “no”) [values: yes / no /
     unclear],
   - notes and text quotations describing search string development
     approach (for at least one literature source) [values: free text],
   - whether explicit recordof search string development is provided for
     at least one online database (e.g. as a table of consecutive search
     string variants used before setting on the final one) [vslues: yes / no
     / unclear],
   - notes and text quotations describing search string development
     record [values: free text],
   - whether a final search stringis provided for at least one source
     (e.g., in text or as a table -this is sometimes included in supplements
     for one or more databases). Note that there are two levels of “no” and
     two levels of “yes” [values: no (no search strategy reported or only
     searched sources) / no (full or partial list of keywords used given in
     text but no exact search) / yes (exact search strategy for at least one
     source) / yes (exact search strategy for all sources) / unclear],
   - notes and text quotations describing where and how a final search
     string is reported for at least one source and/or write a comment (e.g.,
     “Table S1 in SI”) [values: free text],
   - whether a predefined set of papers was used to seedsearch string
     development [values: yes / no / unclear],
   - terms used to describe predefined set of papers used for search
     string development [values: free text / NA],
   - whether full text or SI mentions any type of search string
     sensitivity evaluationfor at least one literature source [values: yes /
     no / unclear],
   - notes and text quotations describing search string sensitivity
     evaluation approach [values:free text],
   - number of literature sources (e.g., online databases/platforms) for
     which search string sensitivity evaluation was conducted [values:
     numeric -integer],
   - names of literature sources (e.g.,online databases/platforms) for
     which search string sensitivity evaluation was conducted [values: free
     text],
   - whether a predefined set of papers was used to evaluate the
     developed search string in terms of sensitivity [values: yes / no /
     unclear],
   - terms used to describe the predefined set of papers used for search
     string sensitivity evaluation [values: free text / NA],
   - terms used to describe the search string sensitivity evaluation
     (e.g., validation, evaluation, testing, benchmarking) [values: free text
     / NA],
   - number of papers used in the predefined set of papers used for
     search string development (either seeding or evaluation) [values:
     numeric -integer],
   - notes on the reported way of assembling the set of papers used for
     search string development (either seeding or evaluation) [values: free
     text / NA],
   - notes and text quotations describing the search string sensitivity
     testing approach [values: free text].
4. Information on whether an information specialist was involved in
   the development of search strategies:

   - whether any information specialist (e.g., a librarian) was involved
     in the systematic review [values: yes / no / unclear],
   - notes and text quotations describing involvement of an information
     specialist [values: free text].

### Data cross-checking

Extracted data were independently cross-checked by a second reviewer
(YY - Survey1, SY - Survey2).  
Disagreements or unclear data were resolved to consensus via comments
provided to the original reviewer and discussion, as needed.

### Data analyses

ML used the R computational environment for final data cleaning,
summaries, analyses, and visualizations, with code versioning via git
and GitHub. For Survey1 and Survey2, we summarized manually coded data
using simple tables of counts. We then visualized the results as bar
plots of proportions. All data processing and visualisation code is
provided below.

# Literature survey results

## Load and check data

```
## Load extracted data (add an associated meta-data table later), preliminary clean and overview:   
mydat <- read.csv(here("data", "Benchmarking_lit_survey_(cross-checked) - Form Responses 1.csv")) 
#str(mydat)
#names(mydat)
names(mydat) <- gsub(" ", "_", names(mydat)) #change spaces to _ in the column names
names(mydat) <- gsub("\\.","", names(mydat)) #remove . the column names
names(mydat) <- gsub("Searchsources","Search_sources", names(mydat)) #remove . the column names

mydat <- mydat %>% select(!ends_with("1")) #remove old empty columns
#names(mydat)

#add a new column for Cochrane vs other journals
mydat$Survey <- ifelse(mydat$Article_journal == "Cochrane Database of Systematic Reviews", "Cochrane", "other")
#table(mydat$Survey)
mydat$Survey <- factor(mydat$Survey) # convert to factor
mydat$Survey <- ordered(mydat$Survey, levels = c("other", "Cochrane")) #reorder levels

#table(mydat$Checker_initials, mydat$Survey, useNA = "always")
#table(mydat$String_development_described, mydat$Survey, useNA = "always")
#table(mydat$String_development_record_provided, mydat$Survey, useNA = "always")
#table(mydat$String_development_seed_papers_used, mydat$Survey, useNA = "always")
#table(mydat$String_sensitivity_evaluation_mentioned, mydat$Survey, useNA = "always") #only one review tested for 1 source, in each Survey
#mydat[mydat$String_sensitivity_evaluation_mentioned == "yes", ] #see 2 reviews that did sensitivity evaluation

#table(mydat$String_sensitivity_evaluation_source_number, mydat$Survey, useNA = "always") #only one review tested for 1 source, in each Survey
#table(mydat$String_sensitivity_test_papers_used, mydat$Survey, useNA = "always") #only one review
#table(mydat$String_sensitivity_test_papers_terminology, mydat$Survey, useNA = "always") #benchmarking, validation
#table(mydat$String_sensitivity_test_papers_number, mydat$Survey, useNA = "always") #105 once, in other journal Survey
#table(mydat$String_sensitivity_test_papers_sources, mydat$Survey, useNA = "always") #known set
#table(mydat$String_sensitivity_test_approach, useNA = "always") #benchmarking
#table(mydat$Information_specialist_involved, mydat$Survey, useNA = "always")
#table(mydat$Final_string_provided, mydat$Survey, useNA = "always") #4 levels of response
#table(mydat$Search_sources, mydat$Survey, useNA = "always") #needs processing
#table(is.na(mydat$Search_sources)) #note no empty values
```

## List of extracted papers for Survey1 and Survey2

### Survey 1 - cross-disciplinary sample of 100 systematic reviews

Create a table of extracted articles for Survery1.

```
mydat %>% filter(Survey == "other") %>% select(Article_DOI, Article_author, Article_journal, Article_title) %>% arrange(Article_author, Article_journal, Article_title) -> mydat_other_articles

#create a html table for mydat_other_articles   
mydat_other_articles %>%
  datatable(extensions = 'Buttons', 
            options = list(
              dom = 'Bfrtip',
              buttons = c('copy', 'csv', 'excel', 'pdf', 'print'),
              pageLength = 20
            ))
```

```
## making a html table for saving SI into the pdf:
# kable(mydat_other_articles, "html") #%>%
  ##kable_styling("striped", position = "left") #%>%
  ##scroll_box(width = "100%", height = "1500px")
```

### Survey 2 - sample of 100 Cochrane Reviews protocols

```
mydat %>% filter(Survey == "Cochrane") %>% select(Article_DOI, Article_author, Article_journal, Article_title) %>% arrange(Article_author, Article_journal, Article_title) -> mydat_Cochrane_articles

#create a html table for mydat_other_articles   
mydat_Cochrane_articles %>%
  datatable(extensions = 'Buttons', 
            options = list(
              dom = 'Bfrtip',
              buttons = c('copy', 'csv', 'excel', 'pdf', 'print'),
              pageLength = 20
            ))
```

```
## making a html table for saving SI into the pdf:
# kable(mydat_Cochrane_articles, "html") #%>%
  ##kable_styling("striped", position = "left") #%>%
  ##scroll_box(width = "100%", height = "1500px")
```

## Process and plot extracted data for each key extracted vairiable

### String\_development\_described

```
panel_String_development_described <- 
  mydat %>% 
  group_by(Survey) %>%
  count(String_development_described) %>%
  ggplot(aes(x = Survey, 
             y = n, 
             fill = String_development_described)) +
  geom_bar(aes(fill = String_development_described), 
           stat = "identity", 
           position = "fill") + # use , position = "fill" for proportion plot
  coord_flip() + 
  scale_fill_manual(values = c("#21281D", "#BAAF9F"),
                    guide = guide_legend(reverse = TRUE)) +
  theme_bw() +
  theme(legend.position = "top", 
        legend.box = "horizontal", 
        axis.text = element_text(size = 10)) +
  labs(fill = "Search string development described in detail?") +
  labs(x = "Survey", 
       y = "Article proportion")  

panel_String_development_described
```

### String\_development\_record\_provided

```
panel_String_development_record_provided <- 
  mydat %>% 
  group_by(Survey) %>%
  count(String_development_record_provided) %>%
  ggplot(aes(x = Survey, 
             y = n, 
             fill = String_development_record_provided)) +
  geom_bar(aes(fill = String_development_record_provided), 
           stat = "identity", 
           position = "fill") + # use , position = "fill" for proportion plot
  coord_flip() + 
  scale_fill_manual(values = c("#21281D", "#BAAF9F"),
                    guide = guide_legend(reverse = TRUE)) +
  theme_bw() +
  theme(legend.position = "top", 
        legend.box = "horizontal", 
        axis.text = element_text(size = 10)) +
  labs(fill = "Search string development record provided?") +
  labs(x = "Survey", 
       y = "Article proportion")  

panel_String_development_record_provided
```

### String\_development\_seed\_papers\_used

```
panel_String_development_seed_papers_used <- 
  mydat %>% 
  group_by(Survey) %>%
  count(String_development_seed_papers_used) %>%
  ggplot(aes(x = Survey, 
             y = n, 
             fill = String_development_seed_papers_used)) +
  geom_bar(aes(fill = String_development_seed_papers_used), 
           stat = "identity", 
           position = "fill") + # use , position = "fill" for proportion plot
  coord_flip() + 
  scale_fill_manual(values = c("#21281D", "#BAAF9F"),
                    guide = guide_legend(reverse = TRUE)) +
  theme_bw() +
  theme(legend.position = "top", 
        legend.box = "horizontal", 
        axis.text = element_text(size = 10)) +
  labs(fill = "Seed papers used in string development?") +
  labs(x = "Survey", 
       y = "Article proportion")  

panel_String_development_seed_papers_used
```

### String\_sensitivity\_evaluation\_mentioned

```
panel_String_sensitivity_evaluation_mentioned <- 
  mydat %>% 
  group_by(Survey) %>%
  count(String_sensitivity_evaluation_mentioned) %>%
  ggplot(aes(x = Survey, 
             y = n, 
             fill = String_sensitivity_evaluation_mentioned)) +
  geom_bar(aes(fill = String_sensitivity_evaluation_mentioned), 
           stat = "identity", 
           position = "fill") + # use , position = "fill" for proportion plot
  coord_flip() + 
  scale_fill_manual(values = c("#21281D", "#BAAF9F"),
                    guide = guide_legend(reverse = TRUE)) +
  theme_bw() +
  theme(legend.position = "top", 
        legend.box = "horizontal", 
        axis.text = element_text(size = 10)) +
  labs(fill = "String sensitivity evaluation mentioned?") +
  labs(x = "Survey", 
       y = "Article proportion")  

panel_String_sensitivity_evaluation_mentioned
```

### Information\_specialist\_involved

```
panel_Information_specialist_involved <- 
  mydat %>% 
  group_by(Survey) %>%
  count(Information_specialist_involved) %>%
  ggplot(aes(x = Survey, 
             y = n, 
             fill = Information_specialist_involved)) +
  geom_bar(aes(fill = Information_specialist_involved), 
           stat = "identity", 
           position = "fill") + # use , position = "fill" for proportion plot
  coord_flip() + 
  scale_fill_manual(values = c("#21281D", "#BAAF9F"),
                    guide = guide_legend(reverse = TRUE)) +
  theme_bw() +
  theme(legend.position = "top", 
        legend.box = "horizontal", 
        axis.text = element_text(size = 10)) +
  labs(fill = "Information specialist mentioned?") +
  labs(x = "Survey", 
       y = "Article proportion")  

panel_Information_specialist_involved
```

### Final\_string\_provided

```
#wacolors$diablo #preview colour-blind scale used

panel_Final_string_provided <- 
  mydat %>% 
  group_by(Survey) %>%
  count(Final_string_provided) %>%
  ggplot(aes(x = Survey, 
             y = n, 
             fill = Final_string_provided)) +
  geom_bar(aes(fill = Final_string_provided), 
           stat = "identity", 
           position = "fill") + # use , position = "fill" for proportion plot
  coord_flip() + 
  guides(fill = guide_legend(ncol = 2)) +
  scale_fill_manual(values = c("#172512", "#005929", "#279085", "#87C8D8"), #four custom colors
                    guide = guide_legend(reverse = TRUE)) +
  theme_bw() +
  theme(legend.position = "top", 
#        legend.direction = "vertical",
#        legend.box = "horizontal", 
        axis.text = element_text(size = 10)) +
  labs(fill = "Final search strings provided?") +
  labs(x = "Survey", 
       y = "Article proportion")  

panel_Final_string_provided
```

### Search\_sources

```
#table(mydat$Search_sources, mydat$Survey, useNA = "always") #needs processing

# process Search_sources
mydat$Scopus <- str_count(mydat$Search_sources, pattern = "Scopus")
mydat$WoS <- str_count(mydat$Search_sources, pattern = "Web of Science")
mydat$WoS <- ifelse(mydat$WoS == 0, 0, 1) #collapse to 2 levels: 0, 1
mydat$WHO_ICTRP <- str_count(mydat$Search_sources, pattern = "WHO")
mydat$WHO_ICTRP <- ifelse(mydat$WHO_ICTRP == 0, 0, 1) #collapse to 2 levels: 0, 1
mydat$PubMed <- str_count(mydat$Search_sources, pattern = "PubMed")
mydat$EbscoHost <- str_count(mydat$Search_sources, pattern = "EbscoHost")
mydat$OVID_Embase <- str_count(mydat$Search_sources, pattern = "OVID")
mydat$OVID_Embase <- ifelse(mydat$OVID == 0, 0, 1) #collapse to 2 levels: 0, 1
#mydat$ScienceDirect <- str_count(mydat$Search_sources, pattern = "ScienceDirect") #code as "other" below
#mydat$ProQuest <- str_count(mydat$Search_sources, pattern = "ProQuest")  #code as "other" below
mydat$other <- str_count(str_to_lower(mydat$Search_sources), pattern = c("sciencedirect|proquest|other|researchers|authors|references|related|registry|handsearching|internet|websites|google|scholar|personal|similar|open|zenodo|cnki|pistemonikos|unspecified|chinese|iran|islamic|nhs|briggs|xiv|abstracts|conference|ultimate|grey|wanfang|square|dimensions"))
#table(mydat$other, mydat$Survey)

#approx. how many sources used per article?
mydat$sum_sources <- mydat$Scopus + mydat$WoS + mydat$WHO_ICTRP + mydat$PubMed + mydat$EbscoHost + mydat$OVID_Embase + mydat$other 
min(mydat$sum_sources)
```

```
## [1] 1
```

```
max(mydat$sum_sources)
```

```
## [1] 14
```

```
median(mydat$sum_sources)
```

```
## [1] 5
```

```
#hist(mydat$sum_sources)

mydat %>%
  ggplot( aes(x = sum_sources, fill = Survey)) +
    geom_histogram(color = "#e9ecef", alpha = 1, position = 'identity', binwidth = 1) +
    scale_fill_manual(values=c("#e9ecef", "#e9ecef")) +
    labs(x = "Number of sources searched", y = "Article proportion", fill = "") + 
    theme_bw() +
    theme(
      legend.position="none",
      panel.spacing = unit(0.1, "lines"),
      strip.text.x = element_text(size = 8)
    )  +
    facet_wrap(~ factor(Survey, levels = c("Cochrane", "other"), 
                  labels = c("Cochrane journal", "Other journals")), ncol = 2)
```

```
#prep for the barplot of counts per main  source type
mydat$other <- ifelse(mydat$other == 0, 0, 1) #collapse to 2 levels: 0, 1 #aggregate all "other" per article into a single count (1)

# Reshape to a long data format
longer_data <- mydat %>% 
  select(Survey, Scopus:other) %>% 
  pivot_longer(Scopus:other, names_to = "Source", values_to = "use") %>%
  arrange(Source, Survey) %>%
  mutate(Use = ifelse(use == 1, "yes", "no"))
print(longer_data)
```

```
## # A tibble: 1,400 × 4
##    Survey Source      use Use  
##    <ord>  <chr>     <dbl> <chr>
##  1 other  EbscoHost     0 no   
##  2 other  EbscoHost     0 no   
##  3 other  EbscoHost     0 no   
##  4 other  EbscoHost     0 no   
##  5 other  EbscoHost     0 no   
##  6 other  EbscoHost     0 no   
##  7 other  EbscoHost     0 no   
##  8 other  EbscoHost     0 no   
##  9 other  EbscoHost     0 no   
## 10 other  EbscoHost     1 yes  
## # ℹ 1,390 more rows
```

```
str(longer_data)
```

```
## tibble [1,400 × 4] (S3: tbl_df/tbl/data.frame)
##  $ Survey: Ord.factor w/ 2 levels "other"<"Cochrane": 1 1 1 1 1 1 1 1 1 1 ...
##  $ Source: chr [1:1400] "EbscoHost" "EbscoHost" "EbscoHost" "EbscoHost" ...
##  $ use   : num [1:1400] 0 0 0 0 0 0 0 0 0 1 ...
##  $ Use   : chr [1:1400] "no" "no" "no" "no" ...
```

```
longer_data$Source <- factor(longer_data$Source, levels = c("other","Scopus","PubMed","WoS","EbscoHost","WHO_ICTRP","OVID_Embase"), ordered = TRUE)

# plot counts from Search_sources
panel_search_sources <- 
  longer_data %>% 
  group_by(Survey, Source) %>%
  count(Use) %>%
  ggplot(aes(x = Source, 
             y = n, 
             fill = Use)) +
  geom_bar(aes(fill = Use), 
           stat = "identity", 
           position = "fill") + # use , position = "fill" for proportion plot 
  facet_wrap(~ factor(Survey, levels = c("Cochrane", "other"), 
                  labels = c("Cochrane journal", "Other journals")), ncol = 2) +
  coord_flip() + 
  scale_fill_manual(values = c("white", "#759C44"), #four custom colors
                    guide = guide_legend(reverse = TRUE)) +
  theme_bw() +
  theme(legend.position = "none", 
        legend.direction = "horizontal",
        legend.box = "horizontal", 
        axis.text = element_text(size = 10)) +
  labs(fill = "used") +
  labs(x = "Sources searched", 
       y = "Article proportion")  

panel_search_sources
```

## Combined figure

Main manuscript figure - ombine all relevant panels to and save.

```
#assemble the panels using patchwork package
figure1 <- (panel_String_development_described /
              panel_String_development_record_provided /
              panel_String_development_seed_papers_used /
              panel_String_sensitivity_evaluation_mentioned /
              panel_Information_specialist_involved /
              panel_Final_string_provided /
              panel_search_sources) +
              plot_annotation(tag_levels = "A") +
  plot_layout(widths = c(1, 1, 1, 1, 1, 1, 1), 
              heights = c(1, 1, 1, 1, 1, 1, 3)) 

#ggsave(plot = figure1, here("plots", "Fig_survey_results_v0.png"), width = 16, height = 19, units = "cm", dpi = "retina", scale = 1.8)
#ggsave(plot = figure1, here("plots", "Fig_survey_results_v0.pdf"), width = 16, height = 19, units = "cm", scale = 1.8)
```

## Statitical tests

Perform Fisher’s Exact Tests for Information\_specialist\_involved and
Final\_string\_provided variables.

```
#Information_specialist_involved vs Survey
M <- table(mydat$Survey, mydat$Information_specialist_involved)
fisher.test(M)
```

```
## 
##  Fisher's Exact Test for Count Data
## 
## data:  M
## p-value < 2.2e-16
## alternative hypothesis: true odds ratio is not equal to 1
## 95 percent confidence interval:
##   8.648036 40.646986
## sample estimates:
## odds ratio 
##   18.24954
```

```
#table(mydat$Final_string_provided, mydat$Survey, useNA = "always") #4 levels of response
#M <- table(mydat$Survey, mydat$Final_string_provided)
#fisher.test(M) #hard to interpret

## Test exact search strategy for at least one source
#Final_string_provided = yes (exact search strategy for at least one source) vs. Survey
M <- table(mydat$Survey, mydat$Final_string_provided == "yes (exact search strategy for at least one source)")
fisher.test(M)
```

```
## 
##  Fisher's Exact Test for Count Data
## 
## data:  M
## p-value < 2.2e-16
## alternative hypothesis: true odds ratio is not equal to 1
## 95 percent confidence interval:
##   7.220758 31.944680
## sample estimates:
## odds ratio 
##    14.8384
```

```
#Final_string_provided = yes (exact search strategy for at least one source) vs. Information_specialist_involved (both surveys)
M <- table(mydat$Final_string_provided == "yes (exact search strategy for at least one source)", mydat$Information_specialist_involved)
fisher.test(M)
```

```
## 
##  Fisher's Exact Test for Count Data
## 
## data:  M
## p-value = 4.994e-08
## alternative hypothesis: true odds ratio is not equal to 1
## 95 percent confidence interval:
##  2.720847 9.916620
## sample estimates:
## odds ratio 
##   5.135776
```

```
## Test exact search strategy for all sources
#Final_string_provided = yes (exact search strategy for all sources) vs. Survey
M <- table(mydat$Survey, mydat$Final_string_provided == "yes (exact search strategy for all sources)")
fisher.test(M)
```

```
## 
##  Fisher's Exact Test for Count Data
## 
## data:  M
## p-value = 0.01899
## alternative hypothesis: true odds ratio is not equal to 1
## 95 percent confidence interval:
##  0.2282208 0.8869901
## sample estimates:
## odds ratio 
##  0.4544329
```

```
#Final_string_provided = yes (exact search strategy for all sources) vs. Information_specialist_involved (both surveys)
M <- table(mydat$Final_string_provided == "yes (exact search strategy for all sources)", mydat$Information_specialist_involved)
fisher.test(M)
```

```
## 
##  Fisher's Exact Test for Count Data
## 
## data:  M
## p-value = 0.7558
## alternative hypothesis: true odds ratio is not equal to 1
## 95 percent confidence interval:
##  0.4559256 1.7065156
## sample estimates:
## odds ratio 
##  0.8847357
```

```
## Cochrane survey
mydat_Cochrane <- mydat %>% filter(Survey == "Cochrane") #subset to Cochrane survey
M <- table(mydat_Cochrane$Final_string_provided, mydat_Cochrane$Information_specialist_involved)
fisher.test(M)
```

```
## 
##  Fisher's Exact Test for Count Data
## 
## data:  M
## p-value = 0.8259
## alternative hypothesis: two.sided
```

```
#Final_string_provided = yes (any)  vs. Information_specialist_involved (Cochrane survey)
M <- table(mydat_Cochrane$Final_string_provided == "yes (exact search strategy for at least one source)" | mydat_Cochrane$Final_string_provided == "yes (exact search strategy for all sources)", mydat_Cochrane$Information_specialist_involved)
fisher.test(M)
```

```
## 
##  Fisher's Exact Test for Count Data
## 
## data:  M
## p-value = 1
## alternative hypothesis: true odds ratio is not equal to 1
## 95 percent confidence interval:
##    0.0000 137.9768
## sample estimates:
## odds ratio 
##          0
```

```
## Other survey
mydat_Other <- mydat %>% filter(Survey == "other") #subset to Other survey
M <- table(mydat_Other$Final_string_provided, mydat_Other$Information_specialist_involved)
fisher.test(M)
```

```
## 
##  Fisher's Exact Test for Count Data
## 
## data:  M
## p-value = 0.03232
## alternative hypothesis: two.sided
```

```
#Final_string_provided = yes (any)  vs. Information_specialist_involved (Other survey)
M <- table(mydat_Other$Final_string_provided == "yes (exact search strategy for at least one source)" | mydat_Other$Final_string_provided == "yes (exact search strategy for all sources)", mydat_Other$Information_specialist_involved)
fisher.test(M)
```

```
## 
##  Fisher's Exact Test for Count Data
## 
## data:  M
## p-value = 0.005853
## alternative hypothesis: true odds ratio is not equal to 1
## 95 percent confidence interval:
##   1.443345 66.237912
## sample estimates:
## odds ratio 
##   6.885311
```

```
library("gplots")
```

```
## 
## Attaching package: 'gplots'
```

```
## The following object is masked from 'package:stats':
## 
##     lowess
```

```
# convert the data as a table
dt <- as.table(as.matrix(M))
# Graph
balloonplot(t(dt), main ="other journals survey", xlab ="Information_specialist_involved", ylab="Final_string_provided",
            text.size = 0.8, label = FALSE, show.margins = TRUE, cum.margins = FALSE)
```

## Session Info

Display all R session information.

```
sessionInfo()
```

```
## R version 4.3.2 (2023-10-31)
## Platform: x86_64-apple-darwin20 (64-bit)
## Running under: macOS Sonoma 14.3.1
## 
## Matrix products: default
## BLAS:   /Library/Frameworks/R.framework/Versions/4.3-x86_64/Resources/lib/libRblas.0.dylib 
## LAPACK: /Library/Frameworks/R.framework/Versions/4.3-x86_64/Resources/lib/libRlapack.dylib;  LAPACK version 3.11.0
## 
## locale:
## [1] en_US.UTF-8/en_US.UTF-8/en_US.UTF-8/C/en_US.UTF-8/en_US.UTF-8
## 
## time zone: Australia/Sydney
## tzcode source: internal
## 
## attached base packages:
## [1] stats     graphics  grDevices utils     datasets  methods   base     
## 
## other attached packages:
##  [1] gplots_3.1.3    patchwork_1.2.0 wacolors_0.3.1  knitr_1.45     
##  [5] DT_0.31         here_1.0.1      lubridate_1.9.3 forcats_1.0.0  
##  [9] stringr_1.5.1   dplyr_1.1.4     purrr_1.0.2     readr_2.1.5    
## [13] tidyr_1.3.1     tibble_3.2.1    ggplot2_3.5.0   tidyverse_2.0.0
## 
## loaded via a namespace (and not attached):
##  [1] sass_0.4.8         utf8_1.2.4         generics_0.1.3     bitops_1.0-7      
##  [5] KernSmooth_2.23-22 gtools_3.9.5       stringi_1.8.3      caTools_1.18.2    
##  [9] hms_1.1.3          digest_0.6.34      magrittr_2.0.3     evaluate_0.23     
## [13] grid_4.3.2         timechange_0.2.0   fastmap_1.1.1      rprojroot_2.0.4   
## [17] jsonlite_1.8.8     fansi_1.0.6        crosstalk_1.2.1    scales_1.3.0      
## [21] jquerylib_0.1.4    cli_3.6.2          rlang_1.1.3        ellipsis_0.3.2    
## [25] munsell_0.5.0      withr_3.0.0        cachem_1.0.8       yaml_2.3.8        
## [29] tools_4.3.2        tzdb_0.4.0         colorspace_2.1-0   pacman_0.5.1      
## [33] vctrs_0.6.5        R6_2.5.1           lifecycle_1.0.4    htmlwidgets_1.6.4 
## [37] pkgconfig_2.0.3    pillar_1.9.0       bslib_0.6.1        gtable_0.3.4      
## [41] glue_1.7.0         highr_0.10         xfun_0.42          tidyselect_1.2.0  
## [45] rstudioapi_0.15.0  farver_2.1.1       htmltools_0.5.7    labeling_0.4.3    
## [49] rmarkdown_2.25     compiler_4.3.2
```
