# Supplementary material for: A practical guide to evaluating sensitivity of literature search strings for systematic reviews using relative recall
Source: Res Synth Methods. 2025 Mar 7;16(1):1–14. doi: 10.1017/rsm.2024.6 (PMC12621535; doi:10.1017/rsm.2024.6)
Supplement: Lagisz et al. supplementary material [file S1759287924000061sup001.zip › Supplementary_File_1.docx]

**SUPPORTING INFORMATION**

For

**TUTORIAL**

A practical guide to evaluating sensitivity of literature search strings for systematic reviews using relative recall

**AUTHORS**

Malgorzata Lagisz^1,2^, Yefeng Yang^1^, Sarah Young^3^, Shinichi Nakagawa^1,2^

^1^ Evolution & Ecology Research Centre and School of Biological, Earth and Environmental Sciences, University of New South Wales, Sydney, NSW 2052, Australia

^2^ Theoretical Sciences Visiting Program, Okinawa Institute of Science and Technology Graduate University, Onna, 904-0495, Japan

^3^ Carnegie Mellon University, Pittsburgh PA, 15213 USA

* Correspondence: M. Lagisz

e-mail: m.lagisz@unsw.edu.au

## Table of contents

[Table of contents 2](#_Toc160200068)

[Overview 3](#_Toc160200069)

[Databases 3](#_Toc160200070)

[Examples 3](#_Toc160200071)

[OVID Embase 7](#_Toc160200072)

[Step1 7](#_Toc160200073)

[Step2 8](#_Toc160200074)

[Step3 9](#_Toc160200075)

[Step4 11](#_Toc160200076)

[Step5 11](#_Toc160200077)

[Step6 13](#_Toc160200078)

[EBSCO Host 16](#_Toc160200079)

[Step1 16](#_Toc160200080)

[Step2 17](#_Toc160200081)

[Step3 20](#_Toc160200082)

[Step4 21](#_Toc160200083)

[Step5 22](#_Toc160200084)

[Step6 25](#_Toc160200085)

[PubMed 32](#_Toc160200086)

[Step1 33](#_Toc160200087)

[Step2 34](#_Toc160200088)

[Step3 37](#_Toc160200089)

[Step4 38](#_Toc160200090)

[Step5 40](#_Toc160200091)

[Step6 42](#_Toc160200092)

[Web of Science Core Collection 45](#_Toc160200093)

[Step1 45](#_Toc160200094)

[Step2 46](#_Toc160200095)

[Step3 50](#_Toc160200096)

[Step4 50](#_Toc160200097)

[Step5 52](#_Toc160200098)

[Step6 54](#_Toc160200099)

[Scopus 57](#_Toc160200100)

[Step1 57](#_Toc160200101)

[Step2 58](#_Toc160200102)

[Step3 61](#_Toc160200103)

[Step4 61](#_Toc160200104)

[Step5 63](#_Toc160200105)

[Step6 66](#_Toc160200106)

[Combining benchmarking across multiple databases 68](#_Toc160200107)

[Databases and benchmarking searches 68](#_Toc160200108)

[Combining benchmarking across databases 69](#_Toc160200109)

## Overview

This document contains hands-on examples for conducting benchmarking (sensitivity evaluation) of search strings for online academic databases. We provide examples for the following five databases/platforms: OVID Embase, EBSCO Host, PubMed, Web of Science Core Collection, Scopus. The online user interface of WHO International Clinical Trials Registry Platform (https://trialsearch.who.int/) and the closely related ClinicalTrials.gov do not allow combining search strings (queries) and there is no easy way for performing benchmarking there, so they are not included in our tutorial.

### Databases

We acknowledge that some of what we call “databases” are search / database platforms connecting multiple databases / collections. Some of them are free (PubMed), some require subscriptions (Scopus - except Author search, Web of Science, OVID Embase), and some include a mix of free and subscription-based databases (EBSCO Host).

We assume users’ basic and general familiarity with using online user interfaces of the above (and similar databases). Where possible, we provide a general link to a database search information and help page, which contains details on the idiosyncrasies of the search string construction and other functionalities of a given database.

We include screenshots of the views of the database online user interfaces alongside the benchmarking stages, showing the inputs and outputs of the key performed actions. However, we note that database interfaces can change, and the screenshots (as well as names of links or buttons) may no longer be accurate at some point in time. However, the general functionalities of each database should be still available, and often improved and expanded, making the examples still valid.

For each database, we provide its general access link (most require subscription and may need to be accessed in a way appropriate to the arrangements of the given subscription, e.g., via institutional library and/or a proxy server).

### Examples

For the first five databases (OVID Embase, EBSCO Host, PubMed, Web of Science Core Collection, Scopus), we will use the same example of a hypothetical scoping systematic review and the same benchmarking set (Table S1). However, each database will be presented as a separate example workflow, with combined results presented at the end as an additional example of combining sensitivity analyses across databases.

Please note that the search examples used are hypothetical and do not represent any real project (but were useful for finding additional relevant references for this work). Similarly, the target search strings used in the examples do not aim to be the final or perfect search strings used in the project – they are more like initial or intermediate search strings that need to be evaluated for sensitivity before further refinement.


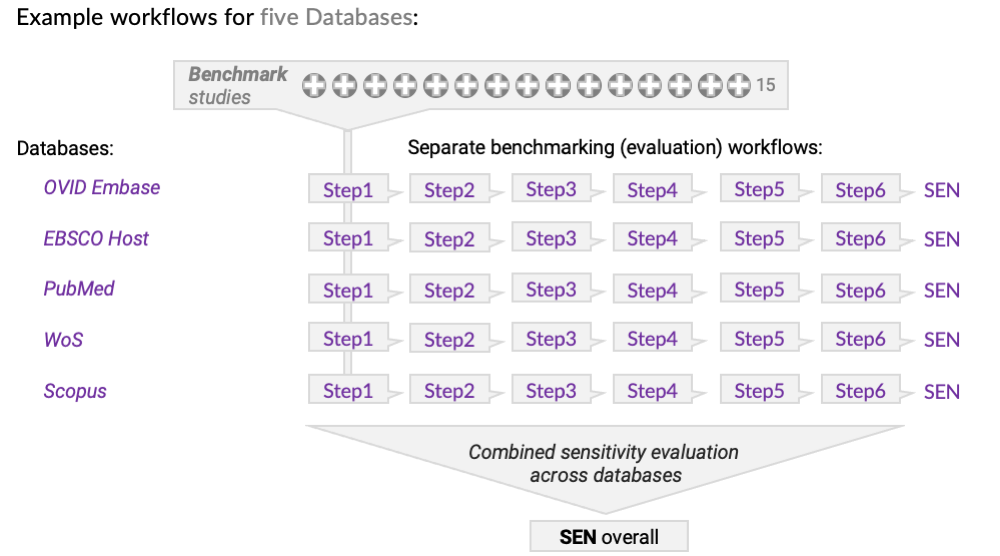


**Figure S1**

*Conceptual schematics of the examples used for the 5 databases. For each database we present an example of simple target search string evaluation (benchmarking), as a stand-alone workflow. We then show how to combine individual database evaluations into an overall estimate of search sensitivity.*

**Table S1**

*List of benchmark papers used in search sensitivity evaluations of a systematic search for a scoping review on the use of search string benchmarking. This benchmarking set is used in the first five examples of workflows for the databases: OVID Embase, EbscoHost, PubMed, Web of Science Core Collection, and Scopus.*

| **Nr** | **Reference** | **DOI** |
| --- | --- | --- |
| 1 | Adams CE, Power A, Frederick K, Lefebvre C. An investigation of the adequacy of MEDLINE searches for randomized controlled trials (RCTs) of the effects of mental health care. Psychol Med. 1994 Aug;24(3):741-8 | 10.1017/s0033291700027896 |
| 2 | Agoritsas T, Merglen A, Courvoisier DS, Combescure C, Garin N, Perrier A, Perneger TV. Sensitivity and predictive value of 15 PubMed search strategies to answer clinical questions rated against full systematic reviews. J Med Internet Res. 2012 Jun 12;14(3):e85 | 10.2196/jmir.2021 |
| 3 | Goossen K, Tenckhoff S, Probst P, Grummich K, Mihaljevic AL, Büchler MW, Diener MK. Optimal literature search for systematic reviews in surgery. Langenbecks Arch Surg. 2018 Feb;403(1):119-129 | 10.1007/s00423-017-1646-x |
| 4 | Harbour J, Fraser C, Lefebvre C, Glanville J, Beale S, Boachie C, Duffy S, McCool R, Smith L. Reporting methodological search filter performance comparisons: a literature review. Health Info Libr J. 2014 Sep;31(3):176-94 | 10.1111/hir.12070 |
| 5 | Hausner E, Guddat C, Hermanns T, Lampert U, Waffenschmidt S. Development of search strategies for systematic reviews: validation showed the noninferiority of the objective approach. J Clin Epidemiol. 2015 Feb;68(2):191-9 | 10.1016/j.jclinepi.2014.09.016 |
| 6 | Haynes RB, Kastner M, Wilczynski NL; Hedges Team. Developing optimal search strategies for detecting clinically sound and relevant causation studies in EMBASE. BMC Med Inform Decis Mak. 2005 Mar 22;5:8 | 10.1186/1472-6947-5-8 |
| 7 | Jenkins M. Evaluation of methodological search filters--a review. Health Info Libr J. 2004 Sep;21(3):148-63 | 10.1111/j.1471-1842.2004.00511.x |
| 8 | Lefebvre C, Glanville J, Beale S, Boachie C, Duffy S, Fraser C, Harbour J, McCool R, Smith L. Assessing the performance of methodological search filters to improve the efficiency of evidence information retrieval: five literature reviews and a qualitative study. Health Technol Assess. 2017 Nov;21(69):1-148 | 10.3310/hta21690 |
| 9 | Li L, Smith HE, Atun R, Tudor Car L. Search strategies to identify observational studies in MEDLINE and Embase. Cochrane Database Syst Rev. 2019 Mar 12;3(3):MR000041 | 10.1002/14651858.MR000041.pub2 |
| 10 | Marson AG, Chadwick DW. How easy are randomized controlled trials in epilepsy to find on Medline? The sensitivity and precision of two Medline searches. Epilepsia. 1996 Apr;37(4):377-80 | 10.1111/j.1528-1157.1996.tb00575.x |
| 11 | Raza MA, Mokhtar R, Ahmad N, Pasha M, Pasha U, A Taxonomy and Survey of Semantic Approaches for Query Expansion. IEEE Access 2019 7:17823-17833 | 10.1109/ACCESS.2019.2894679 |
| 12 | Sampson M, McGowan J. Inquisitio validus Index Medicus: A simple method of validating MEDLINE systematic review searches. Res Synth Methods. 2011 Jun;2(2):103-9 | 10.1002/jrsm.40 |
| 13 | Sampson M, Zhang L, Morrison A, Barrowman NJ, Clifford TJ, Platt RW, Klassen TP, Moher D. An alternative to the hand searching gold standard: validating methodological search filters using relative recall. BMC Med Res Methodol. 2006 Jul 18;6:33 | 10.1186/1471-2288-6-33 |
| 14 | Spoor P, Airey M, Bennett C, Greensill J, Williams R. Use of the capture-recapture technique to evaluate the completeness of systematic literature searches. BMJ. 1996 Aug 10;313(7053):342-3 | 10.1136/bmj.313.7053.342 |
| 15 | Wilczynski NL, McKibbon KA, Walter SD, Garg AX, Haynes RB. MEDLINE clinical queries are robust when searching in recent publishing years. J Am Med Inform Assoc. 2013 Mar-Apr;20(2):363-8 | 10.1136/amiajnl-2012-001075 |

## OVID Embase

### Step1

1. **Collect pre-known relevant studies (benchmarking set)**
   1. **Define the scope of your systematic review (or any systematic-like review using a systematic search approach), its inclusion and exclusion criteria:**

A hypothetical systematic-like review (a scoping review) on literature related to search string evaluations / benchmarking in systematic reviews and meta-analyses.

The scope of this scoping review in **PICo** framework:

- **P**opulation: systematic-like reviewers
- phenomena of **I**nterest: approaches and recommendations for benchmarking search strings
- **Co**ntext: systematic reviews and meta-analyses
  1. **Select the search sources to be used in your systematic review:**

OVID Embase.

Link: <https://ovidsp.ovid.com/>

Searching information and help on conducting searches in this database can be found: <https://ospguides.ovid.com/OSPguides/embase.htm>

- 1. **Decide if search evaluation will be performed for one or more search sources, and which ones:**

OVID Embase only (for now).

- 1. **Gather a set of potential “benchmark” studies from diverse sources. Avoid using the databases you are planning to use as your systematic review search sources:**

The benchmark set of 15 relevant articles presented in **Table S1** has been assembled a priori from personal collections of articles, their reference lists and citations, similarity recommendations, and Google Scholar searches. The articles were pre-selected to represent diverse first authors, journals and disciplines. However, there was no restriction on study type, publication time or language, thus we include empirical, methodological and review articles, published anytime and in any language.

### Step2

1. **Search for the benchmark studies in a database you are evaluating:**
   1. **Create a benchmarking search string from all ID numbers (e.g., DOI) of the benchmark studies, using “OR” Boolean operator:**

*("10.1017/s0033291700027896".do.) OR ("10.2196/jmir.2021".do.) OR ("10.1007/s00423-017-1646-x".do.) OR ("10.1111/hir.12070".do.) OR ("10.1016/j.jclinepi.2014.09.016".do.) OR ("10.1186/1472-6947-5-8".do.) OR ("10.1111/j.1471-1842.2004.00511.x".do.) OR ("10.3310/hta21690".do.) OR ("10.1002/14651858.MR000041.pub2".do.) OR ("10.1111/j.1528-1157.1996.tb00575.x".do.) OR ("10.1109/ACCESS.2019.2894679".do.) OR ("10.1002/jrsm.40".do.) OR ("10.1186/1471-2288-6-33".do.) OR ("10.1136/bmj.313.7053.342".do.) OR ("10.1136/amiajnl-2012-001075".do.)*

- 1. **If a benchmark study is not found by its ID, it is either because of the true absence of the study record or incorrect/missing ID. Thus, for each incorrect/missing benchmark study run a search using its title or other identifying details (e.g., author, year). If found, check if the ID is correct and fix/replace the ID if needed, then search again by ID only. Pay attention to other potential issues, such as duplicated records, or single ID representing collections of works (e.g. conference abstracts book). Continue checking and refining this sub-step until you have a benchmark search string that retrieves all benchmark studies present in each database:**

Results of the initial search using 15 DOI as search terms: 12 articles found.


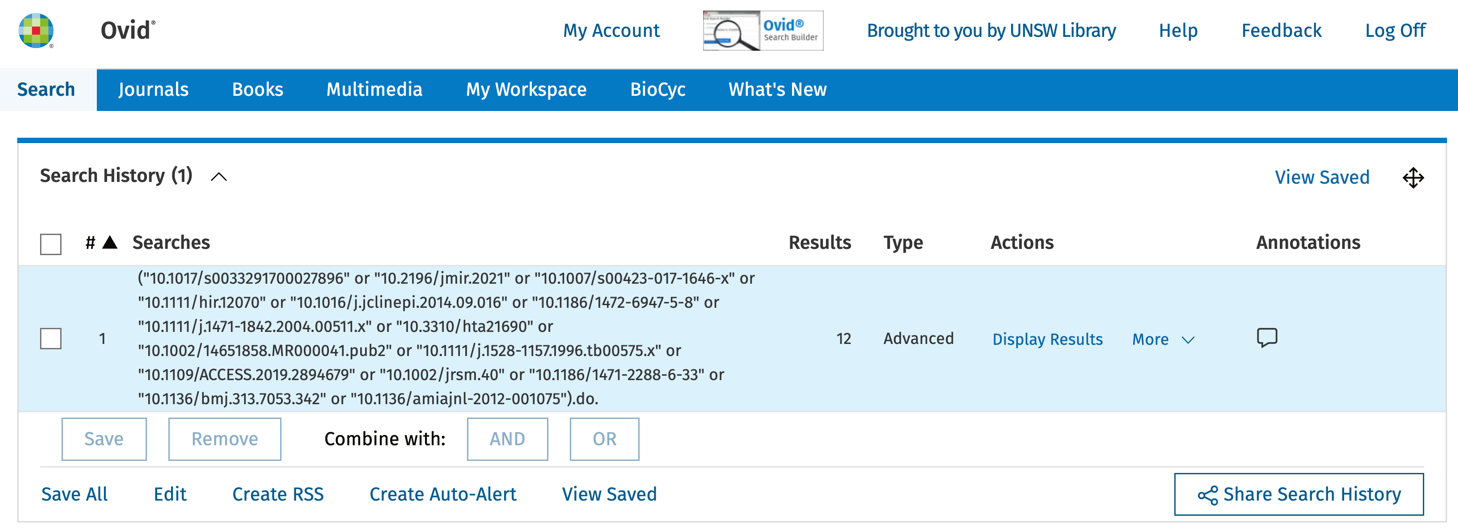


***Screenshot OVID Embase 1.***

*Search History window with an initial benchmarking search string.*

After identifying the missing benchmark articles, you can run a title-based search for each one in turn. IF found, you can check whether it has a correct DOI number or any other ID number which could be used instead of a DOI. If a title search does not bring up the record, you can try searching by a combination of author name and year or journal. If you cannot find the article in a database, it is likely not there – i.e. it is a missing benchmark.

Missing benchmark articles and comments on the reasons:

1. Adams et al. (1994) - 10.1017/s0033291700027896 – searched by title and found. This article has no DOI indexed in this database, but has PMID number (.pm.), which can be used instead (7991756).

2. Raza et al. (2019) - 10.1109/ACCESS.2019.2894679 – could not find by title – not indexed in this database.

3. Sampson et al. (2011) - 10.1002/jrsm.40 – could not find by title – not indexed in this database.

Revised benchmarking search string:

*("7991756".pm.) OR ("10.2196/jmir.2021".do.) OR ("10.1007/s00423-017-1646-x".do.) OR ("10.1111/hir.12070".do.) OR ("10.1016/j.jclinepi.2014.09.016".do.) OR ("10.1186/1472-6947-5-8".do.) OR ("10.1111/j.1471-1842.2004.00511.x".do.) OR ("10.3310/hta21690".do.) OR ("10.1002/14651858.MR000041.pub2".do.) OR ("10.1111/j.1528-1157.1996.tb00575.x".do.) OR ("10.1109/ACCESS.2019.2894679".do.) OR ("10.1002/jrsm.40".do.) OR ("10.1186/1471-2288-6-33".do.) OR ("10.1136/bmj.313.7053.342".do.) OR ("10.1136/amiajnl-2012-001075".do.)*

This search strings returns 13 (out of 15) benchmark articles which are indexed in Ovid EMBASE.

- 1. **Optional: Repeat for each database that will be used in search string evaluations:**

Not applicable (single database evaluation).

### Step3

1. **Remove absent benchmark studies, keep the rest (i.e. customise your benchmarking set for each database).**
   1. **You can do this by simply removing IDs of the missing benchmark studies from a search string for a given database. This way you will have a clean benchmark search string with the IDs matching all benchmark studies present in a given database, which will make your search refinement and calculations easier:**

Revised benchmarking search string:

*("7991756".pm.) OR ("10.2196/jmir.2021".do.) OR ("10.1007/s00423-017-1646-x".do.) OR ("10.1111/hir.12070".do.) OR ("10.1016/j.jclinepi.2014.09.016".do.) OR ("10.1186/1472-6947-5-8".do.) OR ("10.1111/j.1471-1842.2004.00511.x".do.) OR ("10.3310/hta21690".do.) OR ("10.1002/14651858.MR000041.pub2".do.) OR ("10.1111/j.1528-1157.1996.tb00575.x".do.) OR ("10.1186/1471-2288-6-33".do.) OR ("10.1136/bmj.313.7053.342".do.) OR ("10.1136/amiajnl-2012-001075".do.)*


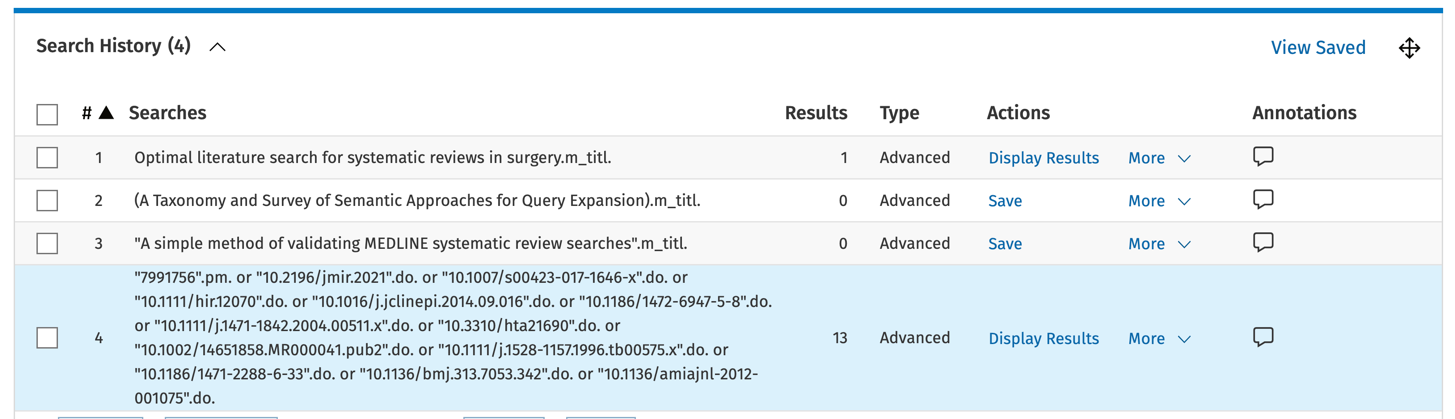


***Screenshot OVID Embase 2.***

*Search History window with a list of used search strings. The revised search string is number 4.*

- 1. **Alternatively, you can just note which and how many benchmark studies are missing from a given database, and later adjust your search string refinements and calculations accordingly:**

Not applicable (removed the missing benchmarks).

- 1. **If relevant, set aside any benchmark studies that are absent from all of the databases you had planned to search. You can come back to these later to determine where they can be found (e.g. a grey literature source, an unindexed journal) and to determine if additional sources should be searched for your review.**

### Step4

1. **Run your target search string on a database.**
   1. **Typically, your target search string is a string composed by combining review scope-related terms (e.g., keywords, fixed expressions, controlled vocabulary, etc.) using Boolean (AND, OR) or other operators and field filters (e.g., which part of the bibliographic record to search, and any additional search limitations, like publication years or subject areas):**

Target search string:

*(search* and (benchmark* or "gold standard" or "golden-standard set" or "gold studies" or "validation set" or "test set" or "comparator set" or "reference standard records" or "seed documents" or "seed studies") and (database* or retriev* or find*) and (valid* or test* or assess* or compar* or effic* or success* or "relative recall" or "recall ratio" or sensitiv*))*

This will be interpreted using Default Fields for Unqualified Searches (MP):**“**Searching for a term without specifying a field in Advanced search, or specifying .mp., defaults to the following ‘multi-purpose’ (.mp.) fields for this database: ti,ab,hw,tn,ot,dm,mf,dv,kf,fx,dq.” (<https://ospguides.ovid.com/OSPguides/embase.htm>). This preliminary and rough search string results in 5,912 hits.

- 1. **The number of returned records (“hits”) can vary vastly and you should keep track of it for later target search string refinement.**

Although some graphical user interfaces of search engines allow saving or sharing search history, there is no way to annotate them on the go with custom comments (e.g., how and why a given search string has been changed or other issues). It is usually easy enough to copy and paste search history into an independent document (e.g., a spreadsheet) and add comments and notes. This spreadsheet can be used for multiple search sessions, including search dates and outcomes, list of benchmarking articles and sensitivity estimates.

### Step5

1. **Find the benchmark studies among the target search results.**
   1. **This step tests the overlap between records retrieved by the target search string and the benchmark set. Here we can simply combine the two strings. For example, if StringA is a target search string to be evaluated for recall, and if StringB retrieves bibliographic records for all benchmark studies by using their ID numbers, then running a combined search sting in a format “(StringA) AND (StringB)” will retrieve the records that overlap between the two:**

Combining search strings can be done easily by going to Search History and ticking the boxes next to the benchmarking set search and the target search and then pressing button “AND” at the bottom after “Combine with:” at the bottom of the Search History view. This will add an extra row to the search history with the numbers of the combined queries and the resulting number of combined hits.

The overlap between the benchmarking search string and the target search string is 5, and the found benchmark studies are:

- - - Harbour et al. (2014)
    - Jenkins (2004)
    - Li et al. (2019)
    - Sampson et al. (2006)
    - Wilczynski et al. (2013)


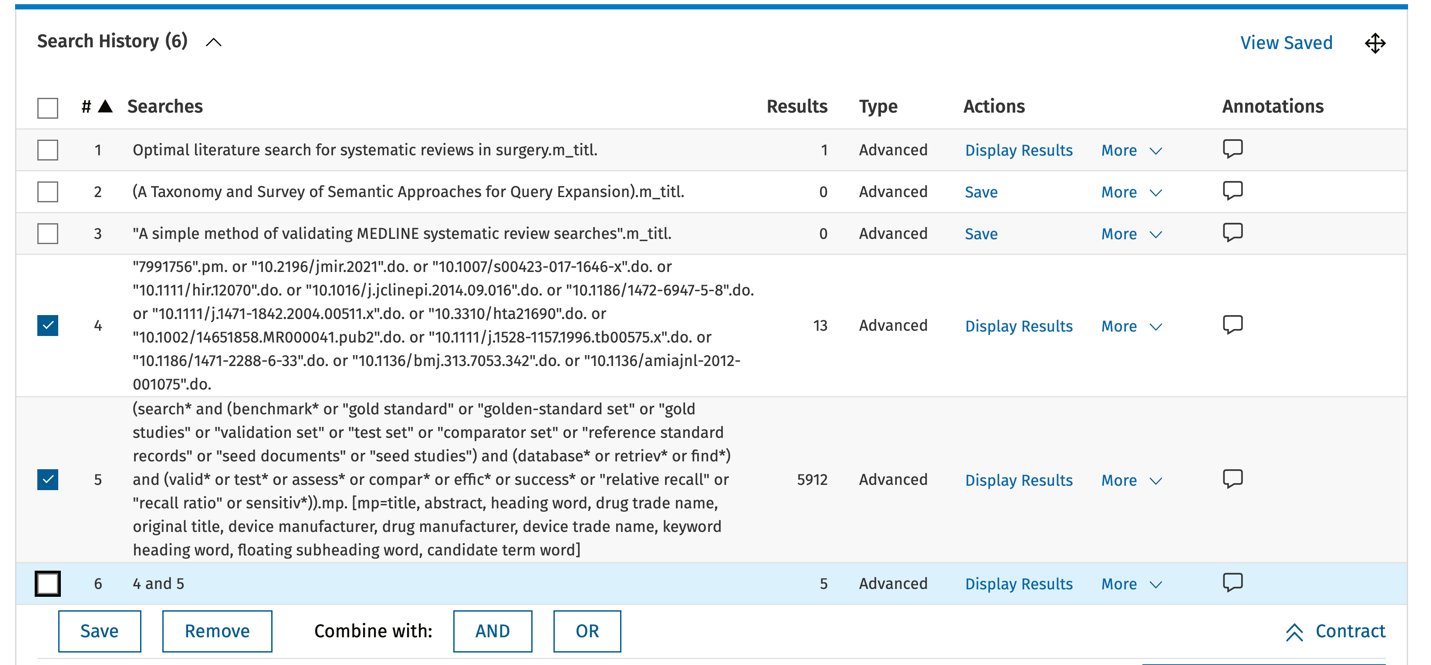


***Screenshot OVID Embase 3.***

*Search History window with buttons for combining saved queries at the bottom.*

- 1. **Optional: If some benchmark records are missing, you can sometimes use the "NOT" operator to see which one are missing (i.e. "(StringB) NOT (StringA)"):**

There is no button for combining search queries with the “NOT” operator, but you can type manually in the advanced search query box: “4 not 5”.

### Step6

1. **Calculate sensitivity of the target search string.**
   1. **The number of overlapping records between the two search strings (target and benchmarking) is the number of the benchmark studies found by the evaluated target string (StringA). Thus, this number, divided by the total number of records retrieved by benchmarking string (StringB) is the estimate of your search sensitivity (SEN or relative recall):**

Since the overlap is 5 records, the sensitivity is 5/13 = 0.38, or 38%.

- 1. **Optional: You can iteratively modify your target search string (StringA). At every iteration, it is very easy to re-evaluate new target StringA against benchmarking StringB using the same method as above (combining the strings). When modifying your search string, you can start by reading through the titles and abstracts of these missed studies. Determine why the study was missed by your current search strategy. What terms are missing from your search string? If reasonable, add the missing search terms to your search (e.g., add terms that are synonyms of concepts already included in your search, expand proximity windows, adjust stemming, etc.). If there is no reasonable way to adjust the search to capture the study, make a note of this as a potential limitation of your search strategy. You can also see which benchmark papers were found:**

To see the found benchmark papers either scroll down to the bottom of the page or click on the link to “Display Results” in the Search History, which will take you to the same place.


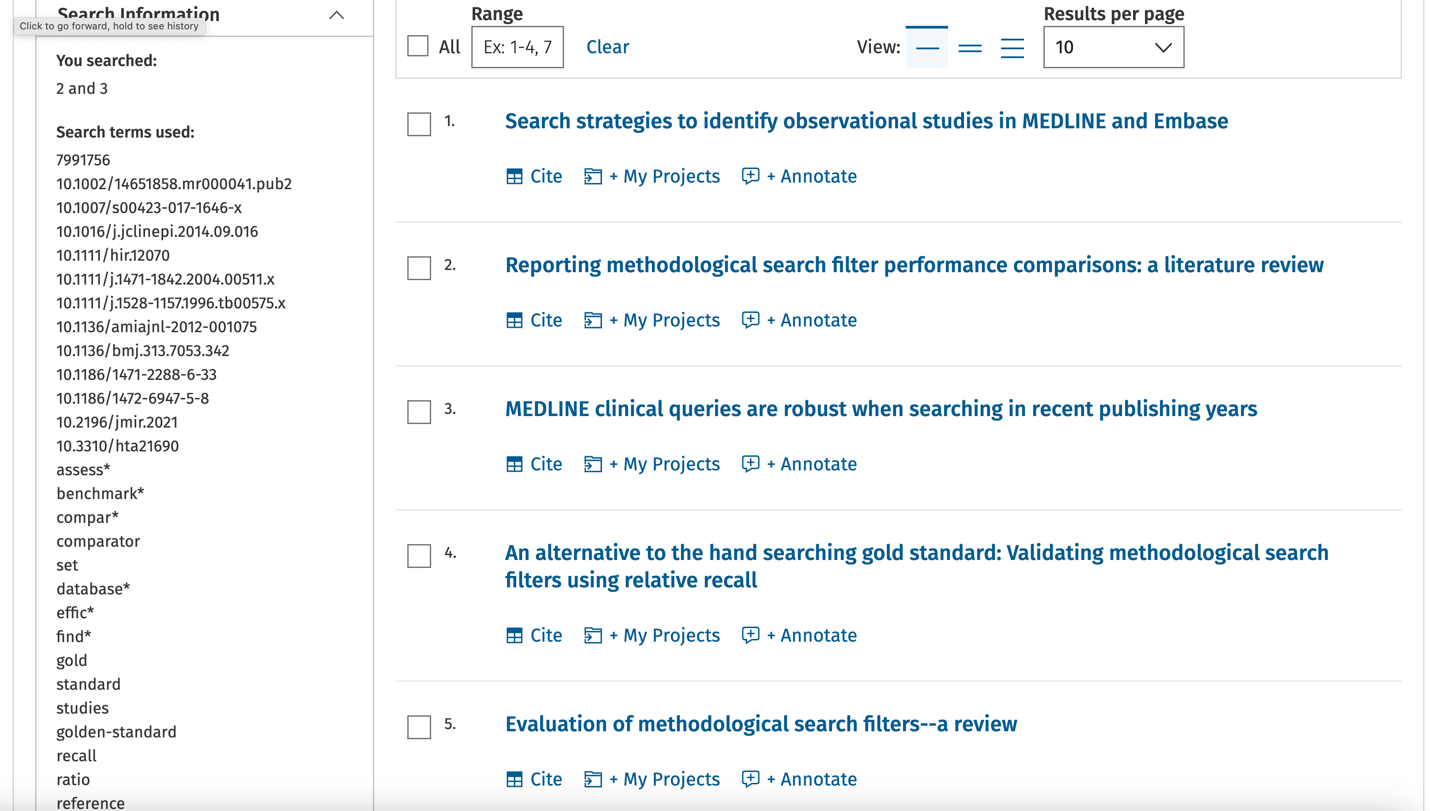


***Screenshot OVID Embase 4.***

*Displaying search results.*

- 1. **Recommended: Keep a good record of the search development and testing process (e.g., in a table), so you can document it transparently in your systematic review or protocol:**

Ovid EMBASE allows sharing Search History, by emailing it, saving a link or copying the history. For example, copied and pasted search history may look like this:

*Embase Classic+Embase <1947 to 2024 January 05>*

*1 ("10.1017/s0033291700027896" or "10.2196/jmir.2021" or "10.1007/s00423-017-1646-x" or "10.1111/hir.12070" or "10.1016/j.jclinepi.2014.09.016" or "10.1186/1472-6947-5-8" or "10.1111/j.1471-1842.2004.00511.x" or "10.3310/hta21690" or "10.1002/14651858.MR000041.pub2" or "10.1111/j.1528-1157.1996.tb00575.x" or "10.1109/ACCESS.2019.2894679" or "10.1002/jrsm.40" or "10.1186/1471-2288-6-33" or "10.1136/bmj.313.7053.342" or "10.1136/amiajnl-2012-001075").do. 12*

*2 "7991756".pm. or "10.2196/jmir.2021".do. or "10.1007/s00423-017-1646-x".do. or "10.1111/hir.12070".do. or "10.1016/j.jclinepi.2014.09.016".do. or "10.1186/1472-6947-5-8".do. or "10.1111/j.1471-1842.2004.00511.x".do. or "10.3310/hta21690".do. or "10.1002/14651858.MR000041.pub2".do. or "10.1111/j.1528-1157.1996.tb00575.x".do. or "10.1186/1471-2288-6-33".do. or "10.1136/bmj.313.7053.342".do. or "10.1136/amiajnl-2012-001075".do. 13*

*3 (search* and (benchmark* or "gold standard" or "golden-standard set" or "gold studies" or "validation set" or "test set" or "comparator set" or "reference standard records" or "seed documents" or "seed studies") and (database* or retriev* or find*) and (valid* or test* or assess* or compar* or effic* or success* or "relative recall" or "recall ratio" or sensitiv*)).mp. [mp=title, abstract, heading word, drug trade name, original title, device manufacturer, drug manufacturer, device trade name, keyword heading word, floating subheading word, candidate term word] 5912*

*4 2 and 3 5*

Same search history, manually saved in a table may look like this:

| **Date / database** | **Search nr** | **Search string** | **Search result / comment** |
| --- | --- | --- | --- |
| 2024 January 05 / Embase Classic+Embase <1947 to 2024 January 05> | 1 | *("10.1017/s0033291700027896" or "10.2196/jmir.2021" or "10.1007/s00423-017-1646-x" or "10.1111/hir.12070" or "10.1016/j.jclinepi.2014.09.016" or "10.1186/1472-6947-5-8" or "10.1111/j.1471-1842.2004.00511.x" or "10.3310/hta21690" or "10.1002/14651858.MR000041.pub2" or "10.1111/j.1528-1157.1996.tb00575.x" or "10.1109/ACCESS.2019.2894679" or "10.1002/jrsm.40" or "10.1186/1471-2288-6-33" or "10.1136/bmj.313.7053.342" or "10.1136/amiajnl-2012-001075").do.* | 12 /  12 out of 15 benchmark studies found, 3 benchmark studies not found |
|  | 2 | *"7991756".pm. or "10.2196/jmir.2021".do. or "10.1007/s00423-017-1646-x".do. or "10.1111/hir.12070".do. or "10.1016/j.jclinepi.2014.09.016".do. or "10.1186/1472-6947-5-8".do. or "10.1111/j.1471-1842.2004.00511.x".do. or "10.3310/hta21690".do. or "10.1002/14651858.MR000041.pub2".do. or "10.1111/j.1528-1157.1996.tb00575.x".do. or "10.1186/1471-2288-6-33".do. or "10.1136/bmj.313.7053.342".do. or "10.1136/amiajnl-2012-001075".do.* | 13 / Replaced one DOI with PMID, 2 benchmark studies absent from the database |
|  | 3 | *(search* and (benchmark* or "gold standard" or "golden-standard set" or "gold studies" or "validation set" or "test set" or "comparator set" or "reference standard records" or "seed documents" or "seed studies") and (database* or retriev* or find*) and (valid* or test* or assess* or compar* or effic* or success* or "relative recall" or "recall ratio" or sensitiv*)).mp. [mp=title, abstract, heading word, drug trade name, original title, device manufacturer, drug manufacturer, device trade name, keyword heading word, floating subheading word, candidate term word]* | 5912 / Target search string |
|  | 4 | *2 and 3* | 5 / Sensitivity is 5/13 = 0.38 (38%). Refine target search string |

## EBSCO Host

EBSCO Host is a collection of multiple databases. You can select one or more specific databases to be searched for a faster and more precise search. In this example we will search all databases contained within the author's institutional subscription (slower and less precise).


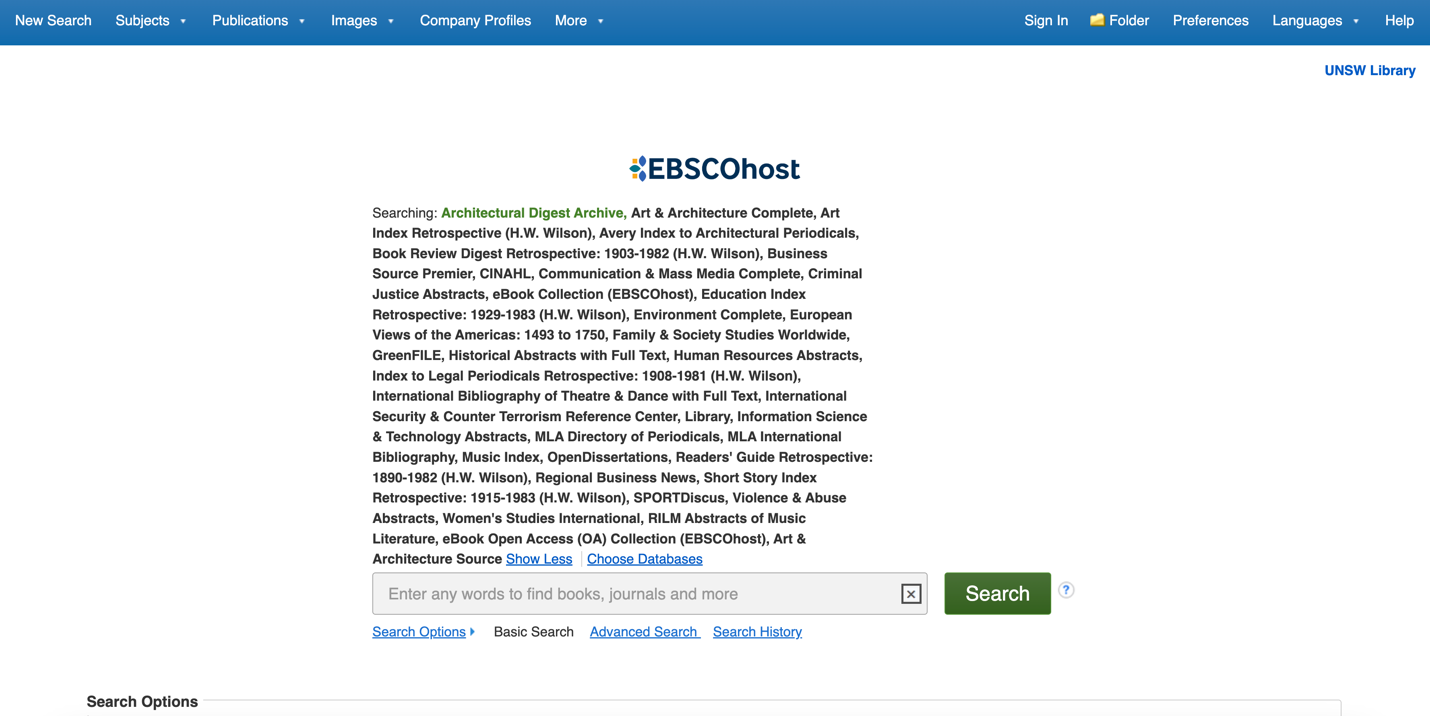


***Screenshot EBSCO Host 1.***

*Basic search window showing all databases selected for searching.*

### Step1

1. **Collect pre-known relevant studies (benchmarking set)**
   1. **Define a scope of your systematic review (or any systematic-like review using a systematic search approach), its inclusion and exclusion criteria:**

A hypothetical systematic-like review (a scoping review) on literature related to search string evaluations / benchmarking in systematic reviews and meta-analyses.

The scope of this scoping review in **PICo** framework:

- **P**opulation: systematic-like reviewers
- phenomena of **I**nterest: approaches and recommendations for benchmarking search strings
- **Co**ntext: systematic reviews and meta-analyses
  1. **Select the search sources to be used in your systematic review:**

EBSCO Host.

Link: <https://www.ebsco.com>, <https://search.ebscohost.com/Login.aspx>

Link to detailed description is available from the Help link in the top right corner of the search window. General searching information and help on conducting searches in this database can be found: <https://connect.ebsco.com>

- 1. **Decide if search evaluation will be performed for one or more search sources, and which ones:**

EBSCO Host only (for now).

- 1. **Gather a set of potential “benchmark” studies from diverse sources. Avoid using the databases you are planning to use as your systematic review search sources:**

The benchmark set of 15 relevant articles presented in **Table S1** has been assembled a priori from personal collections of articles, their reference lists and citations, similarity recommendations, and Google Scholar searches. The articles were pre-selected to represent diverse first authors, journals and disciplines. However, there was not restriction on study type, publication time or language, thus we include empirical, methodological and review articles, published anytime and in any language.

### Step2

1. **Search for the benchmark studies in a database you are evaluating:**
   1. **Create a benchmarking search string from all ID numbers (e.g., DOI) of the benchmark studies, using “OR” Boolean operator:**

EBSCO Host, when used to search multiple databases at once, does not have a search field for DOI or any other database identifiers, so we enter DOI number into first search box with an unspecified field (use DOI when available if you are working with individual databases hosted on EBSCO Host):

*"10.2196/jmir.2021" OR "10.1017/s0033291700027896" OR "10.1007/s00423-017-1646-x" OR "10.1111/hir.12070" OR "10.1016/j.jclinepi.2014.09.016" OR "10.1186/1472-6947-5-8" OR "10.1111/j.1471-1842.2004.00511.x" OR "10.3310/hta21690" OR "10.1002/14651858.MR000041.pub2” OR "10.1111/j.1528-1157.1996.tb00575.x" OR "10.1109/ACCESS.2019.2894679" OR "10.1002/jrsm.40" OR “10.1186/1471-2288-6-33" OR "10.1136/bmj.313.7053.342" OR "10.1136/amiajnl-2012-001075"*

Note that this search string will not work in the Basic Search mode, thus use Advanced Search mode instead. Here, you can enter the whole initial benchmarking search strings constructed from DOI numbers, without specifying the search field.

*
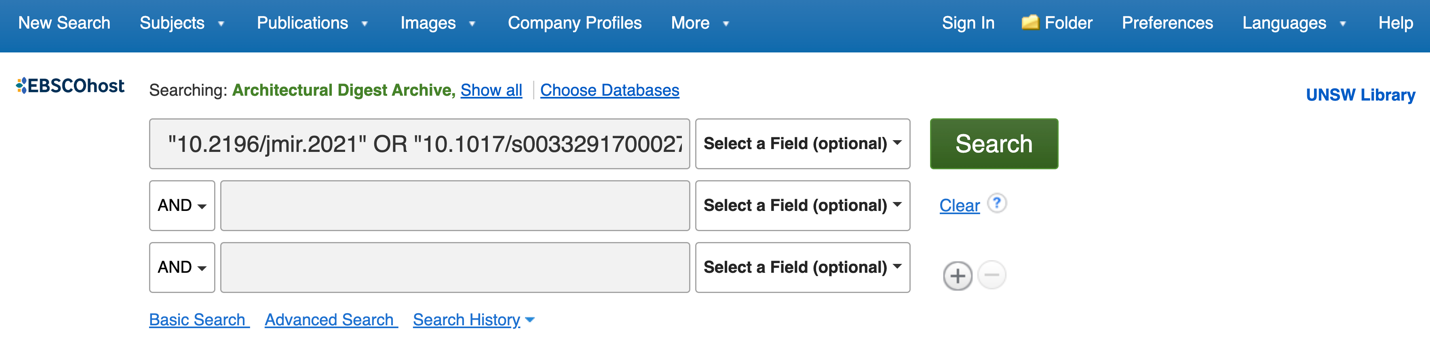
*

***Screenshot EBSCO Host 2.***

*Advanced Search window with an initial benchmarking search string in the first search box with an unspecified Field option.*

- 1. **If a benchmark study is not found by its ID, it is either because of the true absence of the study record or incorrect/missing ID. Thus, for each incorrect/missing benchmark study run a search using its title or other identifying details (e.g., author, year). If found, check if the ID is correct and fix/replace the ID if needed, then search again by ID only. Pay attention to other potential issues, such as duplicated records, or single ID representing collections of works (e.g. conference abstracts book). Continue checking and refining this sub-step until you have a benchmark search string that retrieves all benchmark studies present in each database:**

Results of the initial search using 15 DOI as search terms: 7 articles found.

*
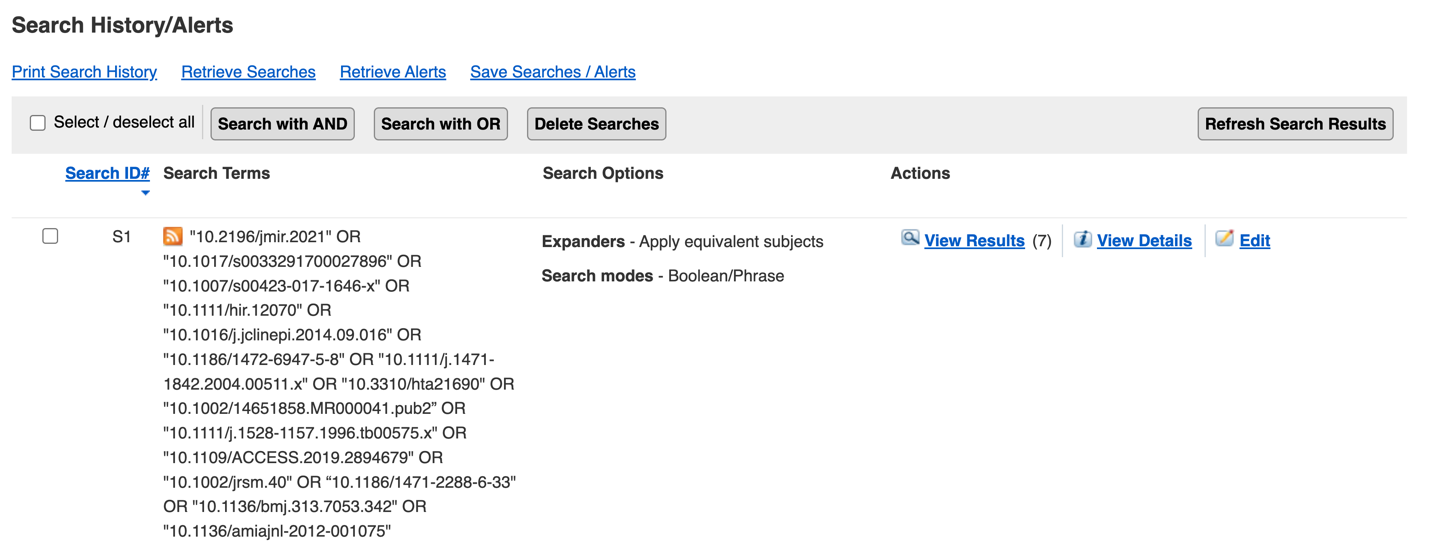
*

***Screenshot EBSCO Host 3.***

*Advanced Search window with results of running an initial benchmarking search string in the first search box with an unspecified Field option. The results appear in the Search History/Alerts section next to S1 Search_ID#.*

After identifying the missing benchmark articles, you can use Basic or Advanced Search mode to run a title-based search for each one in turn. If found, you can check whether it has a correct DOI number or any ither ID number which could be used instead of a DOI. If a title search does not bring up the record, you can try searching by a combination of author name and year or journal. If you cannot find the article in a database, it is likely not there – i.e. it is a missing benchmark.

Missing benchmark articles and comments on the reasons:

1. Haynes et al. (2005) - 10.1186/1472-6947-5-8 – not in the database

2. Lefebvre et al. (2017) - 10.3310/hta21690 – not in the database

3. Li et al. (2019) - 10.1002/14651858.MR000041.pub2 – not in the database

4. Marson et al. (1996) - 10.1111/j.1528-1157.1996.tb00575.x – not in the database

5. Raza et al. (2017) - 10.1109/ACCESS.2019.2894679 – not in the database

6. Sampson et al. (2011) - 10.1002/jrsm.40 – not in the database

7. Sampson et al. (2006) - 10.1186/1471-2288-6-33 – not in the database

8. Spoor et al. (1996) - 10.1136/bmj.313.7053.342 – not in the database

If any of the above benchmark papers were found, we could have used their Accession Number to replace thier DOI in the benchmarking search string: *“The Accession Number (AN) included in EBSCOhost records is an identifying number of an article in the database. If you know the AN of an article, you can search for it by entering AN as a search tag in EBSCOhost's Boolean search screens, followed by the number, for example: AN 1774567.”* (EBSCO Connect What is the Accession Number (AN) in EBSCOhost records?Oct 19, 2018•Knowledge). For example*,* as AN "103875088".

This search strings returns 7 (out of 15) benchmark articles which are indexed in EBSCO Host.

- 1. **Optional: Repeat for each database that will be used in search string evaluations.:**

Not applicable (single database evaluation).

### Step3

1. **Remove absent benchmark studies, keep the rest (i.e. customise your benchmarking set for each database).**
   1. **You can do it by simply removing IDs of the missing benchmark studies from a search string for a given database. This way you will have a clean benchmark search string with the IDs matching all benchmark studies present in a given database, which will make your search refinement and calculations easier:**

Revised benchmarking search string:

*"10.2196/jmir.2021" OR "10.1017/s0033291700027896" OR "10.1007/s00423-017-1646-x" OR "10.1111/hir.12070" OR "10.1016/j.jclinepi.2014.09.016" OR "10.1111/j.1471-1842.2004.00511.x" OR "10.1136/amiajnl-2012-001075"*


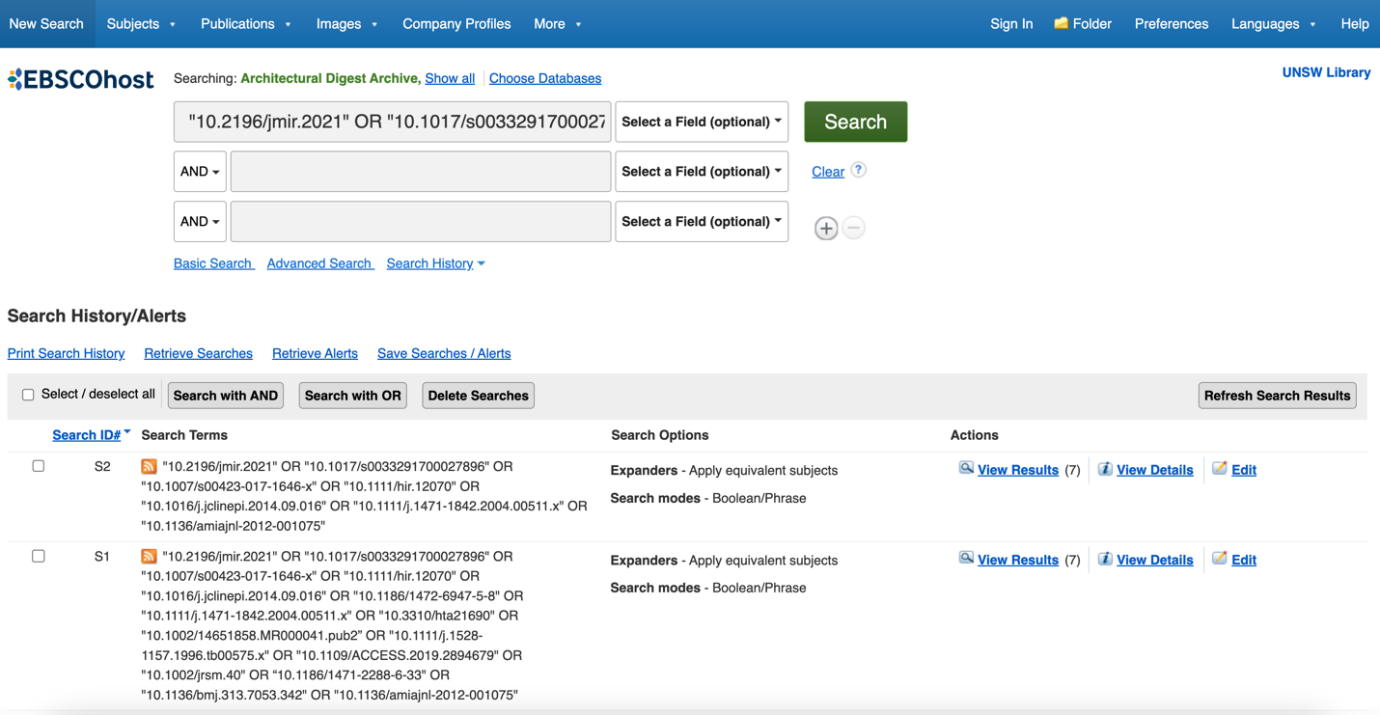


***Screenshot EBSCO Host 4.***

*Advanced Search window with a refined benchmarking search string in the first search box with an unspecified Field option. The results appear in the Search History/Alerts section next to S2 Search_ID#.*

- 1. **Alternatively, you can just note which and how many benchmark studies are missing from a given database, and later adjust your search string refinements and calculations accordingly:**

Not applicable (removed the missing benchmarks).

- 1. **If relevant, set aside any benchmark studies that are absent from all of the databases you had planned to search. You can come back to these later to determine where they can be found (e.g. a grey literature source, an unindexed journal) and to determine if additional sources should be searched for your review.**

### Step4

1. **Run your target search string on a database.**
   1. **Typically, your target search string is a string composed by combining review scope-related terms (e.g., keywords, fixed expressions, controlled vocabulary, etc.) using Boolean (AND, OR) or other operators and field filters (e.g., which part of the bibliographic record to search, and any additional search limitations, like publication years or subject areas):**

Target search string:

*(search* and (benchmark* or "gold standard" or "golden-standard set" or "gold studies" or "validation set" or "test set" or "comparator set" or "reference standard records" or "seed documents" or "seed studies") and (database* or retriev* or find*) and (valid* or test* or assess* or compar* or effic* or success* or "relative recall" or "recall ratio" or sensitiv*))*

If you don’t specify a search Field, this target search string will be interpreted across all searchable fields.

This preliminary and rough search string results in 3,665 hits.


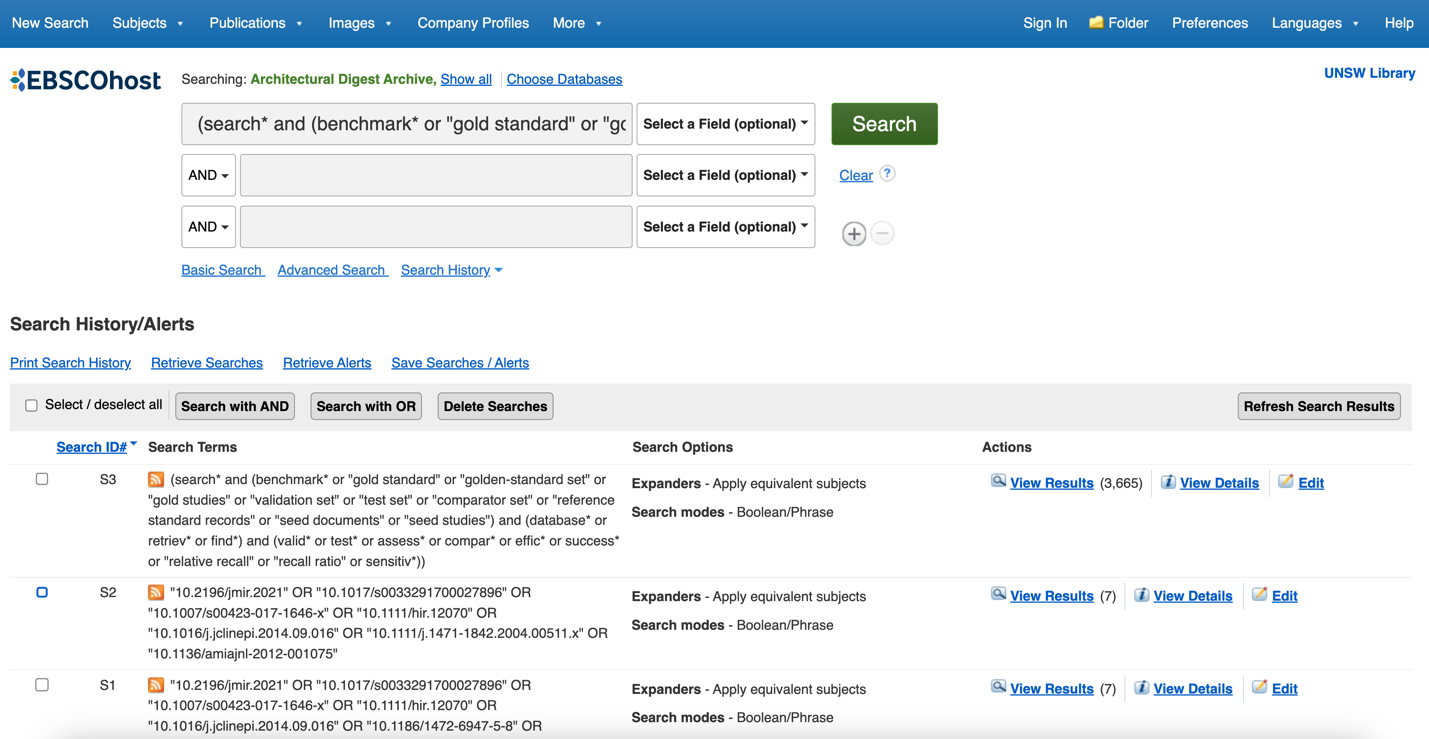


***Screenshot Ebsco Host 4.***

*Advanced Search window with an initial target search string in the first search box with an unspecified Field option. The results appear in the Search History/ Alerts section next to S3 Search_ID#.*

- 1. **The number of returned records (“hits”) can vary vastly and you should keep track of it for later target search string refinement.**

Although some graphical user interfaces of search engines allow saving or sharing search history, there is no way to annotate them on the go with custom comments (e.g., how and why a given search string has been changed or other issues). It is usually easy enough to copy and paste search history into an independent document (e.g., a spreadsheet) and add comments and notes. This spreadsheet can be used for multiple search sessions, including search dates and outcomes, list of benchmarking articles and sensitivity estimates.

### Step5

1. **Find the benchmark studies among the target search results.**
   1. **This step tests the overlap between records retrieved by the target search string and the benchmark set. Here we can simply combine the two strings. For example, if StringA is a target search string to be evaluated for recall, and if StringB retrieves bibliographic records for all benchmark studies by using their ID numbers, then running a combined search sting in a format “(StringA) AND (StringB)” will retrieve the records that overlap between the two:**

This can be done by going to Search History and ticking the boxes next to the benchmarking set search and the target search and then pressing button “AND” at the bottom after “Search with:” at the bottom of the Search History view. This will add an extra row to the search history with the numbers of the combined queries and the resulting number of combined hits. The latter is the overlap, in this case it is 3. You could combine search strings manually by entering their search numbers separated by “AND” Boolean operator in the first search box, without specifying the search Field, e.g.: *S2 and S3*


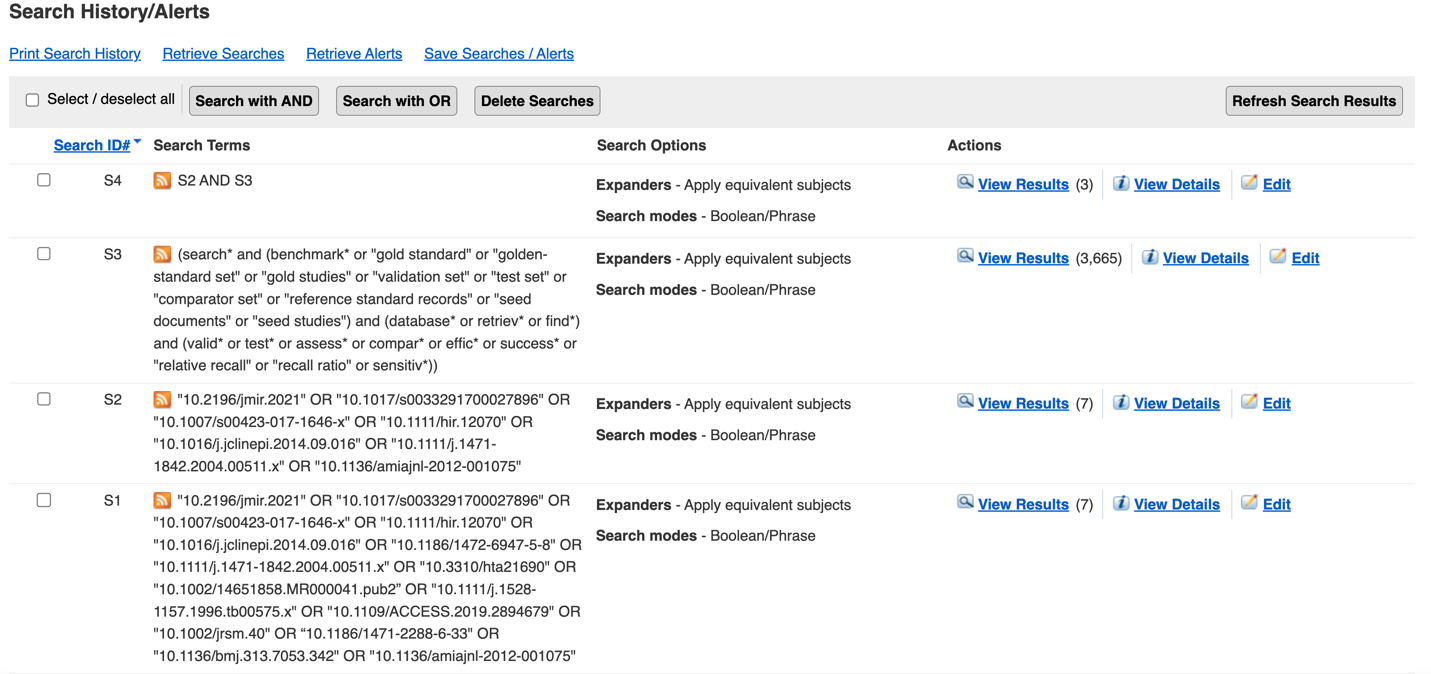


***Screenshot EBSCO Host 5.***

*Combining results of two search queries using Advanced Search window Search History/Alerts section. The two searches to be combined have ticked blue tick-boxes. Make sure the query box on the top of the page is empty (it automatically acts as another “ticked” string to use). Pressing “Search with AND” button above will combine the selected queries using “AND” Boolean operator to reveal the overlap. The results appear in the Search History/Alerts section next to S4 Search_ID#.*

To see the found benchmark papers either scroll down to the bottom of the page or click on the link to “View Results” in the Search History/Alerts, which will take you to the same place.

The overlap between the benchmarking search string and the target search string is 3, and the found benchmark studies are:

- - - Harbour et al. (2014)
    - Jenkins (2004)
    - Wilczynski et al. (2013)
  1. **Optional: If some benchmark records are missing, you can sometimes use "NOT" operator to see which one are missing (i.e. "(StringB) NOT (StringA)"):**

There is no “Search with NOT” button for combining search results, but you can use this operator to combine search results manually by using their search ID numbers in the Advanced Search interface above. E.g., enter: *S2 not S3*. After executing the search, this will return the lit of benchmarks which were not found by the target search string.

**
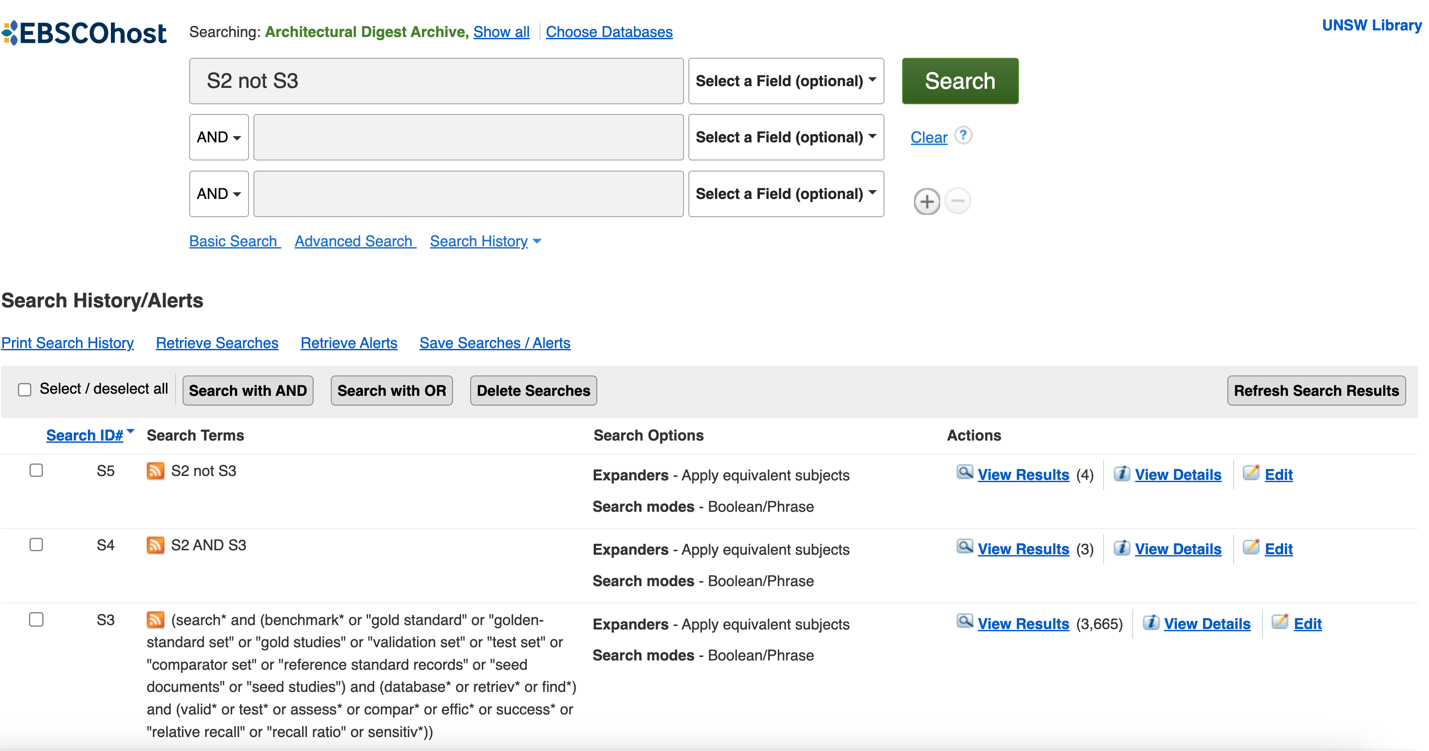
**

***Screenshot EBSCO Host 6.***

*Combining results of two search queries using Advanced Search window Search History/Alerts section. The two searches to be combined have ticked blue tick-boxes. Make sure the query box on the top of the page is empty (it automatically acts as another “ticked” string to use). Manually combine the selected queries using “NOT” Boolean operator to reveal the overlap between the benchmarking search string and the results of the benchmarking operation to see which records are missing. The results appear in the Search History/Alerts section next to S5 Search_ID#.*

To see the missing benchmark papers either scroll down to the bottom of the page or click on the link to “View Results” in the Search History/Alerts, which will take you to the same place.

### Step6

1. **Calculate sensitivity of the target search string.**
   1. **The number of overlapping records between the two search strings (target and benchmarking) is the number of the benchmark studies found by the evaluated target string (StringA). Thus, this number, divided by the total number of records retrieved by benchmarking string (StringB) is the estimate of your search sensitivity (SEN or relative recall):**

Since the overlap is 5 records, the sensitivity is 3/7 = 0.43, or 43%.

- 1. **Optional: You can iteratively modify your target search string (StringA). At every iteration, it is very easy to re-evaluate new target StringA against benchmarking StringB using the same method as above (combining the strings). When modifying your search string, you can start by reading through the titles and abstracts of these missed studies. Determine why the study was missed by your current search strategy. What terms are missing from your search string? If reasonable, add the missing search terms to your search (e.g., add terms that are synonyms of concepts already included in your search, expand proximity windows, adjust stemming, etc.). If there is no reasonable way to adjust the search to capture the study, make a note of this as a potential limitation of your search strategy. You can also see which benchmark papers were found:**

To see the found benchmark papers either scroll down to the bottom of the page or click on the link to “View Results” in the Search History/Alerts, which will take you to the same place.


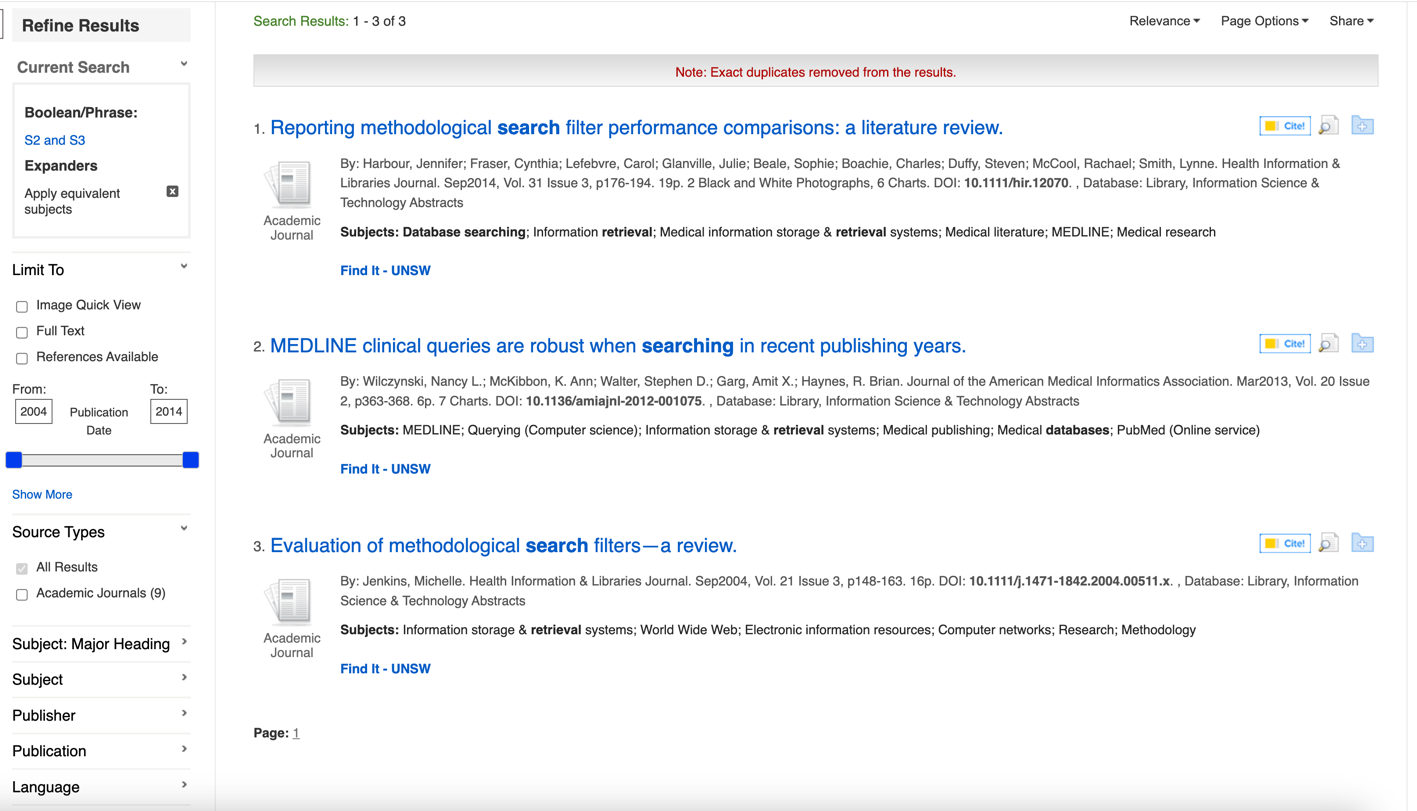


***Screenshot EBSCO Host 7.***

*Displaying search results.*

- 1. **Recommended: Keep a good record of the search development and testing process (e.g., in a table), so you can document it transparently in your systematic review or protocol:**

EBSCO Host allows saving Search History if you have an account in the system. You can also use the option to “Print Search history” to export you search record to an external file (pdf) or copying and pasting a simple history table for your record.

For example, copied and pasted search history may look like this:

| *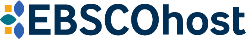* | *Sunday, January 07, 2024 9:13:07 PM* |
| --- | --- |

| ***#*** | ***Query*** | ***Limiters/Expanders*** | ***Last Run Via*** | ***Results*** |
| --- | --- | --- | --- | --- |
| *S5* | *S2 not S3* | *Expanders - Apply equivalent subjects Search modes - Boolean/Phrase* | *Interface - EBSCOhost Research Databases Search Screen - Advanced Search Database - Architectural Digest Archive;Art & Architecture Complete;Art Index Retrospective (H.W. Wilson);Avery Index to Architectural Periodicals;Book Review Digest Retrospective: 1903-1982 (H.W. Wilson);Business Source Premier;CINAHL;Communication & Mass Media Complete;Criminal Justice Abstracts;eBook Collection (EBSCOhost);Education Index Retrospective: 1929-1983 (H.W. Wilson);Environment Complete;European Views of the Americas: 1493 to 1750;Family & Society Studies Worldwide;GreenFILE;Historical Abstracts with Full Text;Human Resources Abstracts;Index to Legal Periodicals Retrospective: 1908-1981 (H.W. Wilson);International Bibliography of Theatre & Dance with Full Text;International Security & Counter Terrorism Reference Center;Library, Information Science & Technology Abstracts;MLA Directory of Periodicals;MLA International Bibliography;Music Index;OpenDissertations;Readers' Guide Retrospective: 1890-1982 (H.W. Wilson);Regional Business News;Short Story Index Retrospective: 1915-1983 (H.W. Wilson);SPORTDiscus;Violence & Abuse Abstracts;Women's Studies International;RILM Abstracts of Music Literature;eBook Open Access (OA) Collection (EBSCOhost);Art & Architecture Source* | *4* |
| *S4* | *S2 AND S3* | *Expanders - Apply equivalent subjects Search modes - Boolean/Phrase* | *Interface - EBSCOhost Research Databases Search Screen - Advanced Search Database - Architectural Digest Archive;Art & Architecture Complete;Art Index Retrospective (H.W. Wilson);Avery Index to Architectural Periodicals;Book Review Digest Retrospective: 1903-1982 (H.W. Wilson);Business Source Premier;CINAHL;Communication & Mass Media Complete;Criminal Justice Abstracts;eBook Collection (EBSCOhost);Education Index Retrospective: 1929-1983 (H.W. Wilson);Environment Complete;European Views of the Americas: 1493 to 1750;Family & Society Studies Worldwide;GreenFILE;Historical Abstracts with Full Text;Human Resources Abstracts;Index to Legal Periodicals Retrospective: 1908-1981 (H.W. Wilson);International Bibliography of Theatre & Dance with Full Text;International Security & Counter Terrorism Reference Center;Library, Information Science & Technology Abstracts;MLA Directory of Periodicals;MLA International Bibliography;Music Index;OpenDissertations;Readers' Guide Retrospective: 1890-1982 (H.W. Wilson);Regional Business News;Short Story Index Retrospective: 1915-1983 (H.W. Wilson);SPORTDiscus;Violence & Abuse Abstracts;Women's Studies International;RILM Abstracts of Music Literature;eBook Open Access (OA) Collection (EBSCOhost);Art & Architecture Source* | *3* |
| *S3* | *(search* and (benchmark* or "gold standard" or "golden-standard set" or "gold studies" or "validation set" or "test set" or "comparator set" or "reference standard records" or "seed documents" or "seed studies") and (database* or retriev* or find*) and (valid* or test* or assess* or compar* or effic* or success* or "relative recall" or "recall ratio" or sensitiv*))* | *Expanders - Apply equivalent subjects Search modes - Boolean/Phrase* | *Interface - EBSCOhost Research Databases Search Screen - Advanced Search Database - Architectural Digest Archive;Art & Architecture Complete;Art Index Retrospective (H.W. Wilson);Avery Index to Architectural Periodicals;Book Review Digest Retrospective: 1903-1982 (H.W. Wilson);Business Source Premier;CINAHL;Communication & Mass Media Complete;Criminal Justice Abstracts;eBook Collection (EBSCOhost);Education Index Retrospective: 1929-1983 (H.W. Wilson);Environment Complete;European Views of the Americas: 1493 to 1750;Family & Society Studies Worldwide;GreenFILE;Historical Abstracts with Full Text;Human Resources Abstracts;Index to Legal Periodicals Retrospective: 1908-1981 (H.W. Wilson);International Bibliography of Theatre & Dance with Full Text;International Security & Counter Terrorism Reference Center;Library, Information Science & Technology Abstracts;MLA Directory of Periodicals;MLA International Bibliography;Music Index;OpenDissertations;Readers' Guide Retrospective: 1890-1982 (H.W. Wilson);Regional Business News;Short Story Index Retrospective: 1915-1983 (H.W. Wilson);SPORTDiscus;Violence & Abuse Abstracts;Women's Studies International;RILM Abstracts of Music Literature;eBook Open Access (OA) Collection (EBSCOhost);Art & Architecture Source* | *3,665* |
| *S2* | *"10.2196/jmir.2021" OR "10.1017/s0033291700027896" OR "10.1007/s00423-017-1646-x" OR "10.1111/hir.12070" OR "10.1016/j.jclinepi.2014.09.016" OR "10.1111/j.1471-1842.2004.00511.x" OR "10.1136/amiajnl-2012-001075"* | *Expanders - Apply equivalent subjects Search modes - Boolean/Phrase* | *Interface - EBSCOhost Research Databases Search Screen - Advanced Search Database - Architectural Digest Archive;Art & Architecture Complete;Art Index Retrospective (H.W. Wilson);Avery Index to Architectural Periodicals;Book Review Digest Retrospective: 1903-1982 (H.W. Wilson);Business Source Premier;CINAHL;Communication & Mass Media Complete;Criminal Justice Abstracts;eBook Collection (EBSCOhost);Education Index Retrospective: 1929-1983 (H.W. Wilson);Environment Complete;European Views of the Americas: 1493 to 1750;Family & Society Studies Worldwide;GreenFILE;Historical Abstracts with Full Text;Human Resources Abstracts;Index to Legal Periodicals Retrospective: 1908-1981 (H.W. Wilson);International Bibliography of Theatre & Dance with Full Text;International Security & Counter Terrorism Reference Center;Library, Information Science & Technology Abstracts;MLA Directory of Periodicals;MLA International Bibliography;Music Index;OpenDissertations;Readers' Guide Retrospective: 1890-1982 (H.W. Wilson);Regional Business News;Short Story Index Retrospective: 1915-1983 (H.W. Wilson);SPORTDiscus;Violence & Abuse Abstracts;Women's Studies International;RILM Abstracts of Music Literature;eBook Open Access (OA) Collection (EBSCOhost);Art & Architecture Source* | *7* |
| *S1* | *"10.2196/jmir.2021" OR "10.1017/s0033291700027896" OR "10.1007/s00423-017-1646-x" OR "10.1111/hir.12070" OR "10.1016/j.jclinepi.2014.09.016" OR "10.1186/1472-6947-5-8" OR "10.1111/j.1471-1842.2004.00511.x" OR "10.3310/hta21690" OR "10.1002/14651858.MR000041.pub2” OR "10.1111/j.1528-1157.1996.tb00575.x" OR "10.1109/ACCESS.2019.2894679" OR "10.1002/jrsm.40" OR “10.1186/1471-2288-6-33" OR "10.1136/bmj.313.7053.342" OR "10.1136/amiajnl-2012-001075"* | *Expanders - Apply equivalent subjects Search modes - Boolean/Phrase* | *Interface - EBSCOhost Research Databases Search Screen - Advanced Search Database - Architectural Digest Archive;Art & Architecture Complete;Art Index Retrospective (H.W. Wilson);Avery Index to Architectural Periodicals;Book Review Digest Retrospective: 1903-1982 (H.W. Wilson);Business Source Premier;CINAHL;Communication & Mass Media Complete;Criminal Justice Abstracts;eBook Collection (EBSCOhost);Education Index Retrospective: 1929-1983 (H.W. Wilson);Environment Complete;European Views of the Americas: 1493 to 1750;Family & Society Studies Worldwide;GreenFILE;Historical Abstracts with Full Text;Human Resources Abstracts;Index to Legal Periodicals Retrospective: 1908-1981 (H.W. Wilson);International Bibliography of Theatre & Dance with Full Text;International Security & Counter Terrorism Reference Center;Library, Information Science & Technology Abstracts;MLA Directory of Periodicals;MLA International Bibliography;Music Index;OpenDissertations;Readers' Guide Retrospective: 1890-1982 (H.W. Wilson);Regional Business News;Short Story Index Retrospective: 1915-1983 (H.W. Wilson);SPORTDiscus;Violence & Abuse Abstracts;Women's Studies International;RILM Abstracts of Music Literature;eBook Open Access (OA) Collection (EBSCOhost);Art & Architecture Source* | *7* |

Same search history, manually saved in a table may look like this:

| **Date / database** | **Search nr** | **Search string** | **Search result / comment** |
| --- | --- | --- | --- |
| *2024 January 08 / Interface - EBSCOhost Research Databases Search Screen - Advanced Search Database - Architectural Digest Archive;Art & Architecture Complete;Art Index Retrospective (H.W. Wilson);Avery Index to Architectural Periodicals;Book Review Digest Retrospective: 1903-1982 (H.W. Wilson);Business Source Premier;CINAHL;Communication & Mass Media Complete;Criminal Justice Abstracts;eBook Collection (EBSCOhost);Education Index Retrospective: 1929-1983 (H.W. Wilson);Environment Complete;European Views of the Americas: 1493 to 1750;Family & Society Studies Worldwide;GreenFILE;Historical Abstracts with Full Text;Human Resources Abstracts;Index to Legal Periodicals Retrospective: 1908-1981 (H.W. Wilson);International Bibliography of Theatre & Dance with Full Text;International Security & Counter Terrorism Reference Center;Library, Information Science & Technology Abstracts;MLA Directory of Periodicals;MLA International Bibliography;Music Index;OpenDissertations;Readers' Guide Retrospective: 1890-1982 (H.W. Wilson);Regional Business News;Short Story Index Retrospective: 1915-1983 (H.W. Wilson);SPORTDiscus;Violence & Abuse Abstracts;Women's Studies International;RILM Abstracts of Music Literature;eBook Open Access (OA) Collection (EBSCOhost);Art & Architecture Source* | 1 | *"10.2196/jmir.2021" OR "10.1017/s0033291700027896" OR "10.1007/s00423-017-1646-x" OR "10.1111/hir.12070" OR "10.1016/j.jclinepi.2014.09.016" OR "10.1186/1472-6947-5-8" OR "10.1111/j.1471-1842.2004.00511.x" OR "10.3310/hta21690" OR "10.1002/14651858.MR000041.pub2” OR "10.1111/j.1528-1157.1996.tb00575.x" OR "10.1109/ACCESS.2019.2894679" OR "10.1002/jrsm.40" OR “10.1186/1471-2288-6-33" OR "10.1136/bmj.313.7053.342" OR "10.1136/amiajnl-2012-001075"*  *Expanders - Apply equivalent subjects Search modes - Boolean/Phrase* | 7 /  7 out of 15 benchmark studies found, 8 benchmark studies not found |
|  | S2 | *"10.2196/jmir.2021" OR "10.1017/s0033291700027896" OR "10.1007/s00423-017-1646-x" OR "10.1111/hir.12070" OR "10.1016/j.jclinepi.2014.09.016" OR "10.1111/j.1471-1842.2004.00511.x" OR "10.1136/amiajnl-2012-001075"*  *Expanders - Apply equivalent subjects Search modes - Boolean/Phrase* | 7 /  7 out of 15 benchmark studies found, 8 benchmark studies not found |
|  | S3 | *(search* and (benchmark* or "gold standard" or "golden-standard set" or "gold studies" or "validation set" or "test set" or "comparator set" or "reference standard records" or "seed documents" or "seed studies") and (database* or retriev* or find*) and (valid* or test* or assess* or compar* or effic* or success* or "relative recall" or "recall ratio" or sensitiv*))*  *Expanders - Apply equivalent subjects Search modes - Boolean/Phrase* | 3665 / Target search string |
|  | S4 | *S2 and S3* | 3 / Sensitivity is 3/7 = 0.43 (43%). Refine target search string |
|  | S5 | *S2 not S4* | 4 missing benchmark papers |

## PubMed

PubMed is a single database which is freely available.


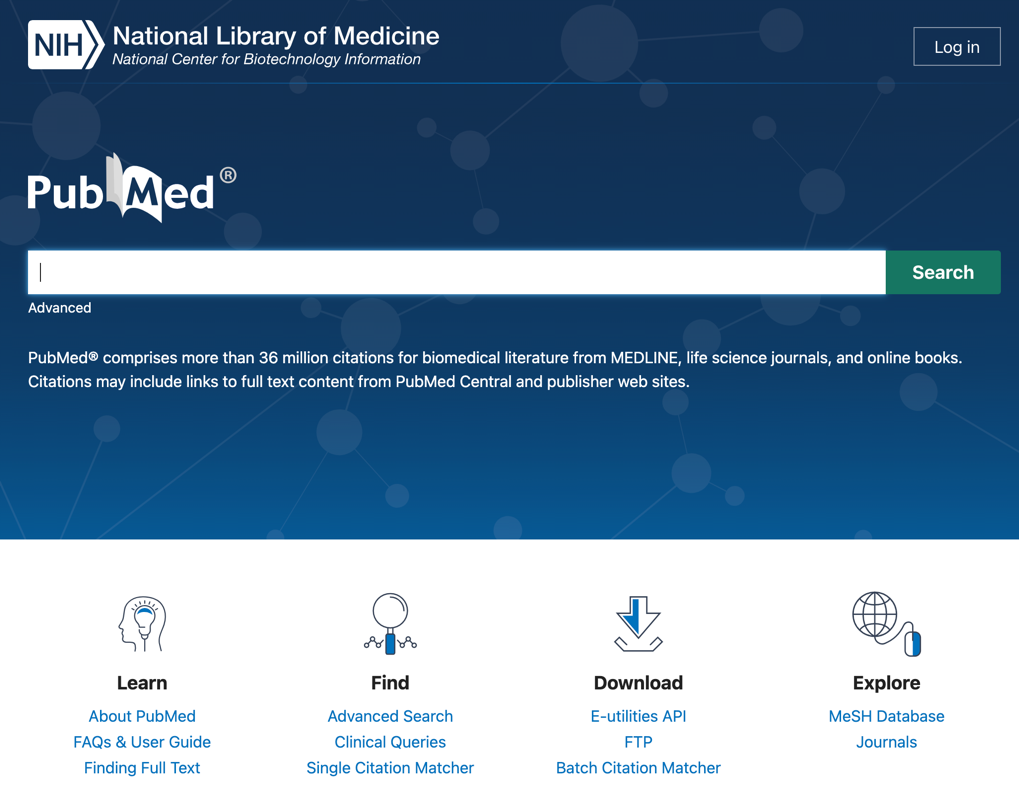


***Screenshot PubMed 1.***

*Home window of PubMed database with a Basic search box.*

### Step1

1. **Collect pre-known relevant studies (benchmarking set)**
   1. **Define a scope of your systematic review (or any systematic-like review using a systematic search approach), its inclusion and exclusion criteria:**

A hypothetical systematic-like review (a scoping review) on literature related to search string evaluations / benchmarking in systematic reviews and meta-analyses.

The scope of this scoping review in **PICo** framework:

- **P**opulation: systematic-like reviewers
- phenomena of **I**nterest: approaches and recommendations for benchmarking search strings
- **Co**ntext: systematic reviews and meta-analyses
  1. **Select the search sources to be used in your systematic review:**

PubMed.

Link: <https://pubmed.ncbi.nlm.nih.gov/>

Link to detailed description and User Guide is available from the FAQ & User Guide in the bottom left of the Home page: <https://pubmed.ncbi.nlm.nih.gov/help/>

- 1. **Decide if search evaluation will be performed for one or more search sources, and which ones:**

PubMed only (for now).

- 1. **Gather a set of potential “benchmark” studies from diverse sources. Avoid using the databases you are planning to use as your systematic review search sources:**

The benchmark set of 15 relevant articles presented in **Table S1** has been assembled a priori from personal collections of articles, their reference lists and citations, similarity recommendations, and Google Scholar searches. The articles were pre-selected to represent diverse first authors, journals and disciplines. However, there was not restriction on study type, publication time or language, thus we include empirical, methodological and review articles, published anytime and in any language.

### Step2

1. **Search for the benchmark studies in a database you are evaluating:**
   1. **Create a benchmarking search string from all ID numbers (e.g., DOI) of the benchmark studies, using “OR” Boolean operator:**

PubMed has a search field for “Article Identifier”, which is specified as “[aid]” field tag. So, we can enter create a following search string using DOI benchmark article numbers:

*"10.2196/jmir.2021"[aid] OR "10.1017/s0033291700027896"[aid] OR "10.1007/s00423-017-1646-x"[aid] OR "10.1111/hir.12070"[aid] OR "10.1016/j.jclinepi.2014.09.016"[aid] OR "10.1186/1472-6947-5-8"[aid] OR "10.1111/j.1471-1842.2004.00511.x"[aid] OR "10.3310/hta21690"[aid] OR "10.1002/14651858.MR000041.pub2” [aid] OR "10.1111/j.1528-1157.1996.tb00575.x"[aid] OR "10.1109/ACCESS.2019.2894679"[aid] OR "10.1002/jrsm.40"[aid] OR “10.1186/1471-2288-6-33"[aid] OR "10.1136/bmj.313.7053.342"[aid] OR "10.1136/amiajnl-2012-001075"[aid]*

Note that this search string will work in the Basic Search mode, or we can use Advanced Search mode instead.

*
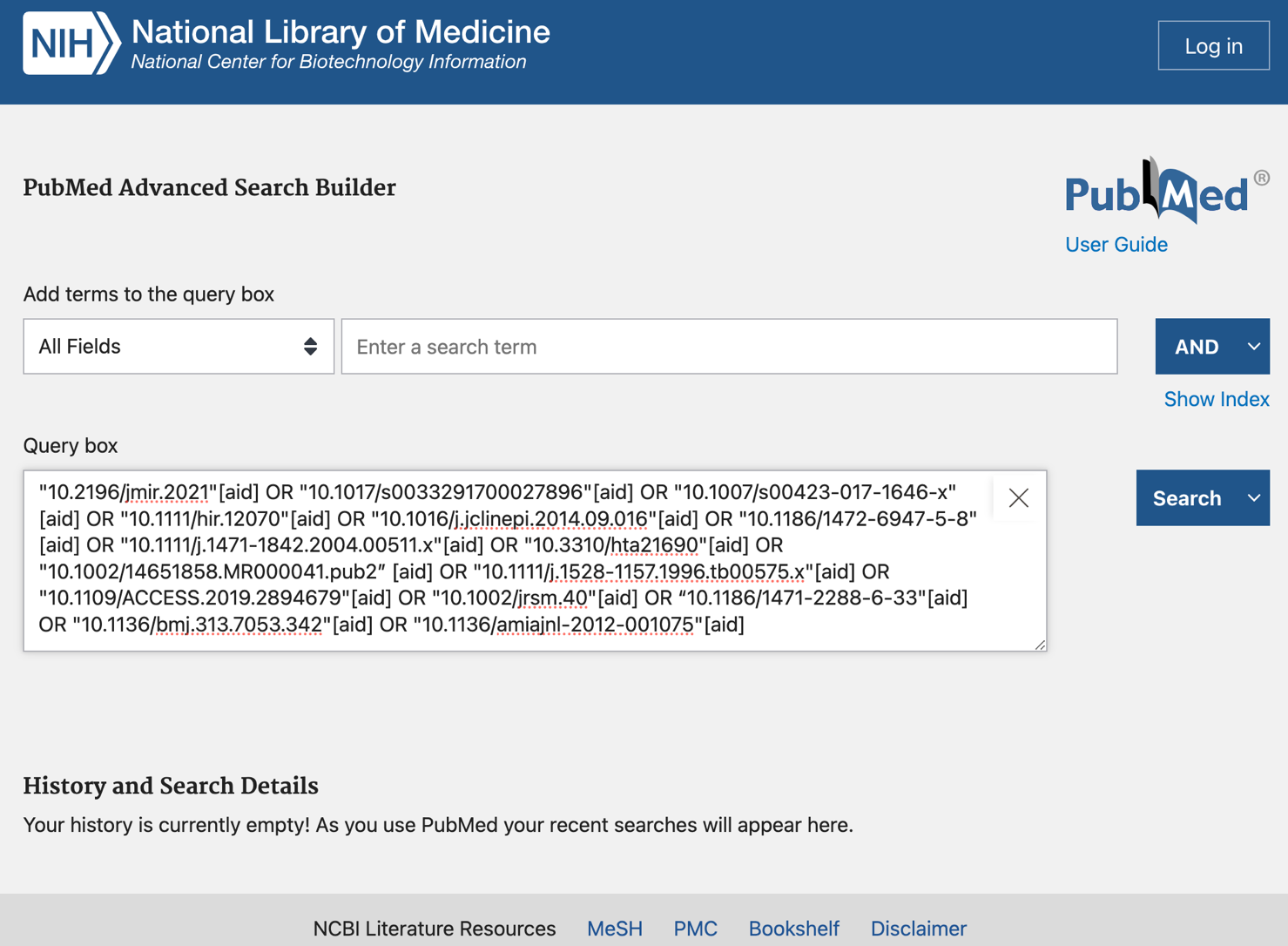
*

***Screenshot PubMed 2.***

*Advanced Search window with an initial benchmarking search string added directly into the Query search box.*

- 1. **If a benchmark study is not found by its ID, it is either because of the true absence of the study record or incorrect/missing ID. Thus, for each incorrect/missing benchmark study run a search using its title or other identifying details (e.g., author, year). If found, check if the ID is correct and fix/replace the ID if needed, then search again by ID only. Pay attention to other potential issues, such as duplicated records, or single ID representing collections of works (e.g. conference abstracts book). Continue checking and refining this sub-step until you have a benchmark search string that retrieves all benchmark studies present in each database:**

Results of the initial search using 15 DOI as search terms: 14 articles found. Note that in this case the database issued a warning specifying which DOI was not found.

*
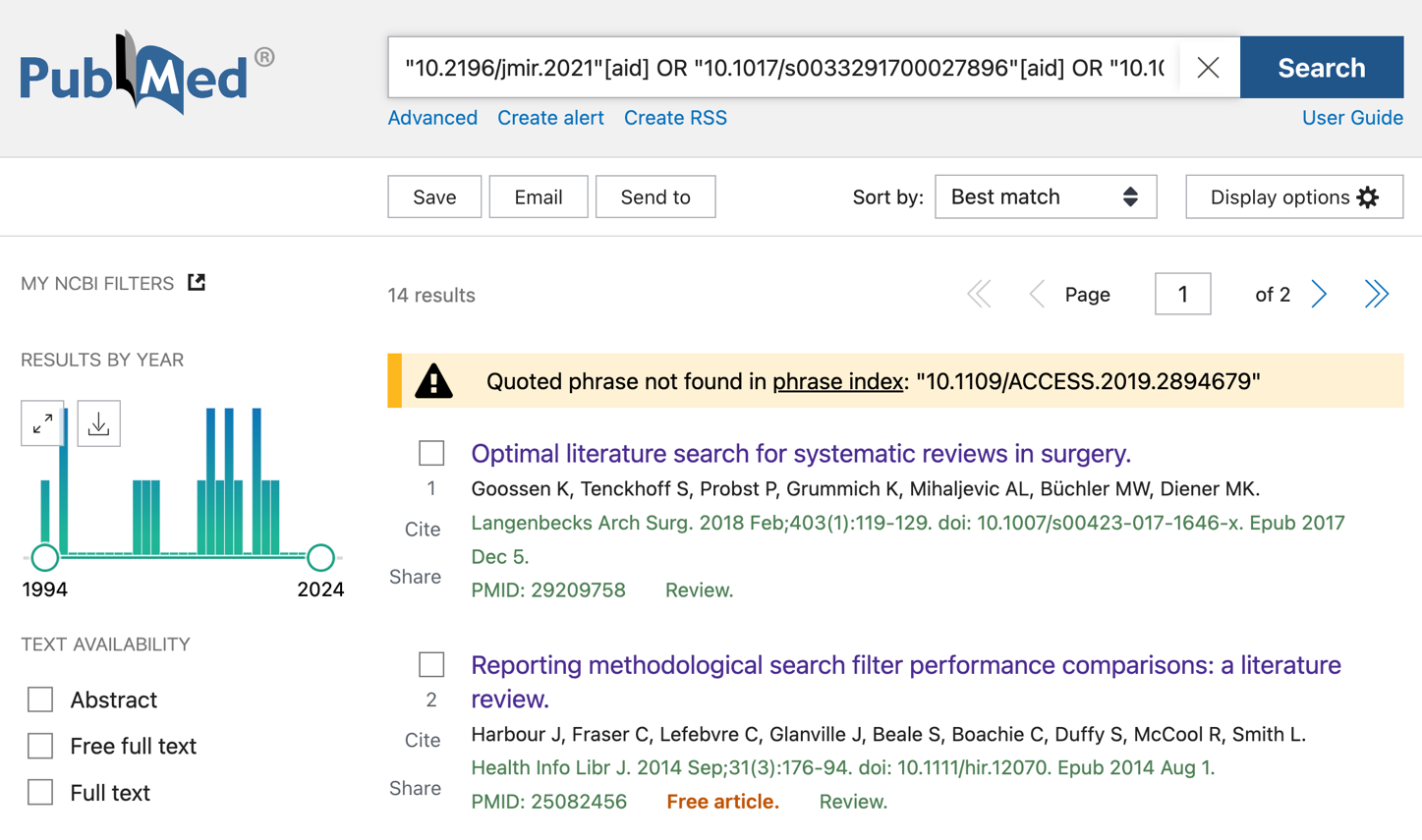
*

***Screenshot PubMed 3.***

*Search results window with results of running a search based on DOI of the benchmarking set of articles.*

After identifying the missing benchmark articles, you can use Basic or Advanced Search mode to run a title-based search for each one in turn. If found, you can check whether it has a correct DOI number or any ither ID number which could be used instead of a DOI. If a title search does not bring up the record, you can try searching by a combination of author name and year or journal. If you cannot find the article in a database, it is likely not there – i.e. it is a missing benchmark.

Missing benchmark articles and comments on the reasons:

1. Raza et al. (2017) - 10.1109/ACCESS.2019.2894679 – not in the database

This search strings returns 14 (out of 15) benchmark articles which are indexed in PubMed.

- 1. **Optional: Repeat for each database that will be used in search string evaluations.:**

Not applicable (single database evaluation).

### Step3

1. **Remove absent benchmark studies, keep the rest (i.e. customise your benchmarking set for each database).**
   1. **You can do it by simply removing IDs of the missing benchmark studies from a search string for a given database. This way you will have a clean benchmark search string with the IDs matching all benchmark studies present in a given database, which will make your search refinement and calculations easier:**

Revised benchmarking search string:

*"10.2196/jmir.2021"[aid] OR "10.1017/s0033291700027896"[aid] OR "10.1007/s00423-017-1646-x"[aid] OR "10.1111/hir.12070"[aid] OR "10.1016/j.jclinepi.2014.09.016"[aid] OR "10.1186/1472-6947-5-8"[aid] OR "10.1111/j.1471-1842.2004.00511.x"[aid] OR "10.3310/hta21690"[aid] OR "10.1002/14651858.MR000041.pub2” [aid] OR "10.1111/j.1528-1157.1996.tb00575.x"[aid] OR "10.1002/jrsm.40"[aid] OR “10.1186/1471-2288-6-33"[aid] OR "10.1136/bmj.313.7053.342"[aid] OR "10.1136/amiajnl-2012-001075"[aid]*


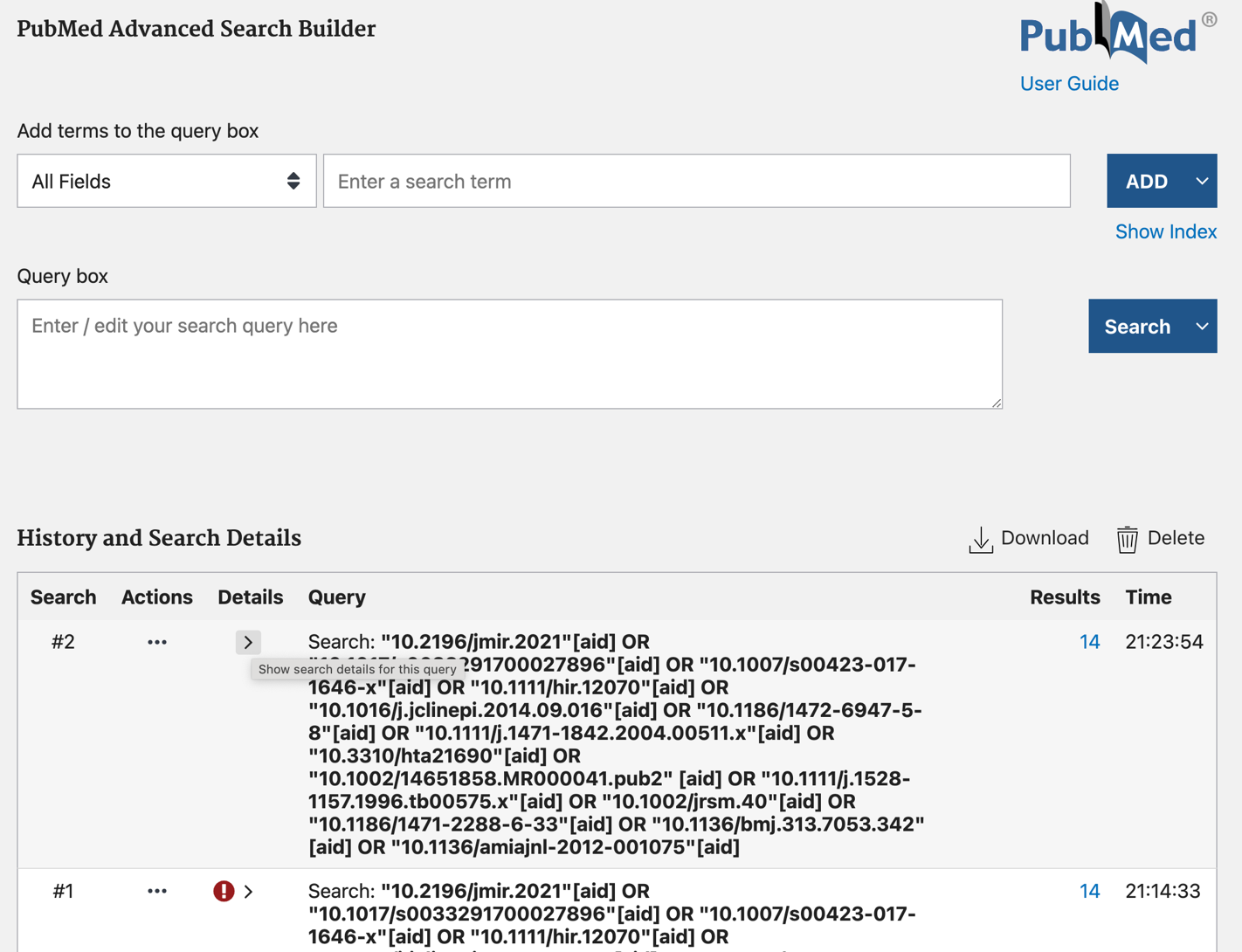


***Screenshot PubMed 4.***

*Advanced Search window after running a refined benchmarking search string. The translated query is added in the Search History and Search Details section next to Search #2.*

- 1. **Alternatively, you can just note which and how many benchmark studies are missing from a given database, and later adjust your search string refinements and calculations accordingly:**

Not applicable (removed the missing benchmarks).

- 1. **If relevant, set aside any benchmark studies that are absent from all of the databases you had planned to search. You can come back to these later to determine where they can be found (e.g. a grey literature source, an unindexed journal) and to determine if additional sources should be searched for your review.**

### Step4

1. **Run your target search string on a database.**
   1. **Typically, your target search string is a string composed by combining review scope-related terms (e.g., keywords, fixed expressions, controlled vocabulary, etc.) using Boolean (AND, OR) or other operators and field filters (e.g., which part of the bibliographic record to search, and any additional search limitations, like publication years or subject areas):**

Target search string (note the use of Boolean operators in capital letters):

*(search* AND (benchmark* OR "gold standard" OR "golden-standard set" OR "gold studies" OR "validation set" OR "test set" OR "comparator set" OR "reference standard records" OR "seed documents" OR "seed studies") AND (database* OR retriev* OR find*) AND (valid* OR test* OR assess* OR compar* OR effic* OR success* OR "relative recall" OR "recall ratio" OR sensitiv*))*

If you don’t specify a search Field, this target search string will be interpreted across all searchable fields.

This preliminary and rough search string results in 8,517 hits.


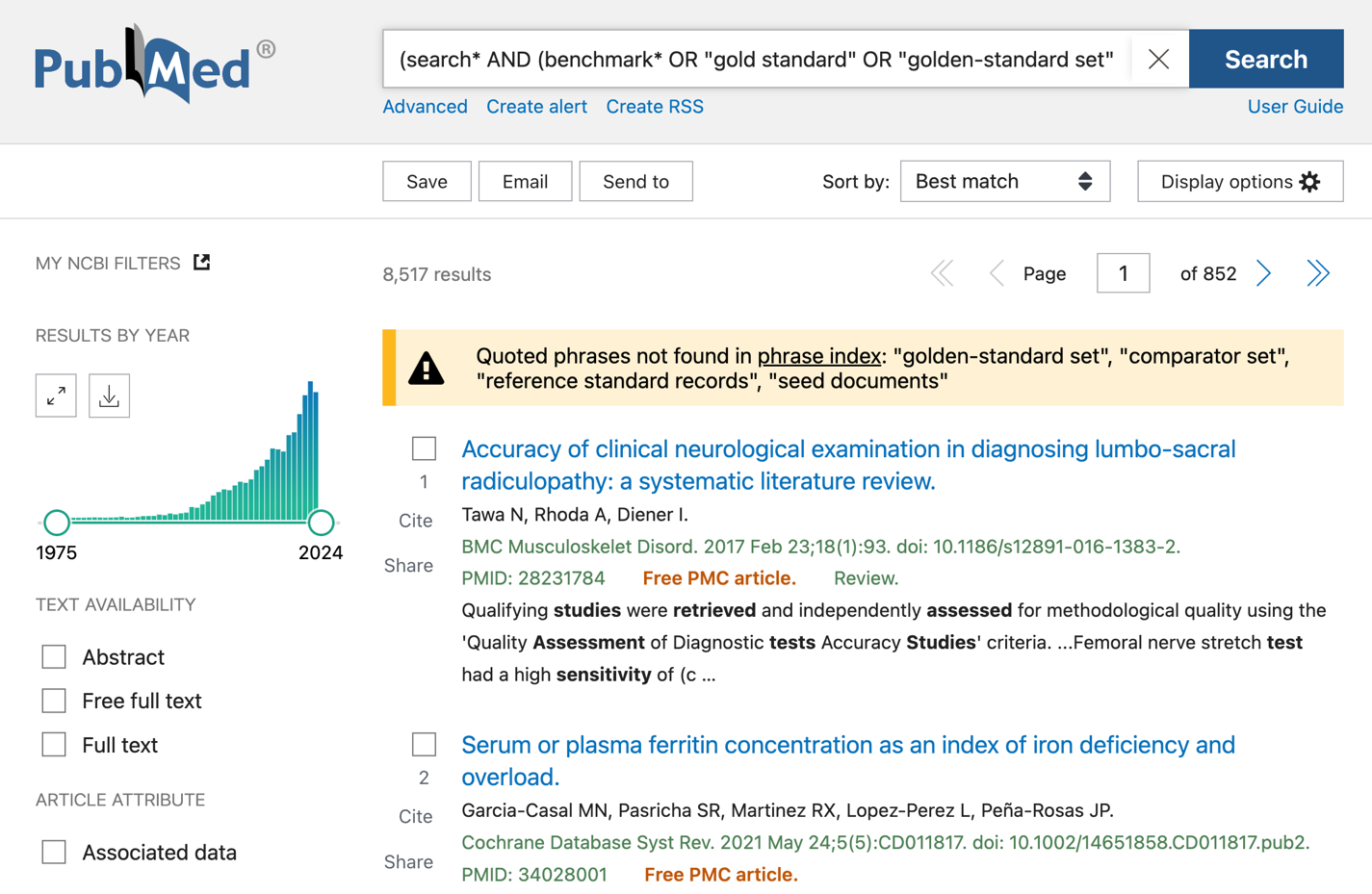


***Screenshot PubMed 5.***

*Results window with an initial target search string in the search box.*

- 1. **The number of returned records (“hits”) can vary vastly and you should keep track of it for later target search string refinement.**

Although some graphical user interfaces of search engines allow saving or sharing search history, there is no way to annotate them on the go with custom comments (e.g., how and why a given search string has been changed or other issues). It is usually easy enough to copy and paste search history into an independent document (e.g., a spreadsheet) and add comments and notes. This spreadsheet can be used for multiple search sessions, including search dates and outcomes, list of benchmarking articles and sensitivity estimates.

### Step5

1. **Find the benchmark studies among the target search results.**
   1. **This step tests the overlap between records retrieved by the target search string and the benchmark set. Here we can simply combine the two strings. For example, if StringA is a target search string to be evaluated for recall, and if StringB retrieves bibliographic records for all benchmark studies by using their ID numbers, then running a combined search sting in a format “(StringA) AND (StringB)” will retrieve the records that overlap between the two:**

This can be done by going to Advanced Search window. It is easiest to use search numbers, as stored in search history, to combine the already executed strings, e.g., by entering in the Query box:

*#1 AND #2*

Alternatively, you could use Actions next to each saved query in History and Search Details section. Select Actions and then Add Query and which Boolean operator to use to combine selected queries.

The overlap between the benchmarking search string and the target search string is 6, and the found benchmark studies are:

- - - Gossen et al. (2018)
    - Harbour et al. (2014)
    - Jenkins (2004)
    - Li et al. (2019)
    - Sampson et al. (2006)
    - Wilczynski et al. (2013)


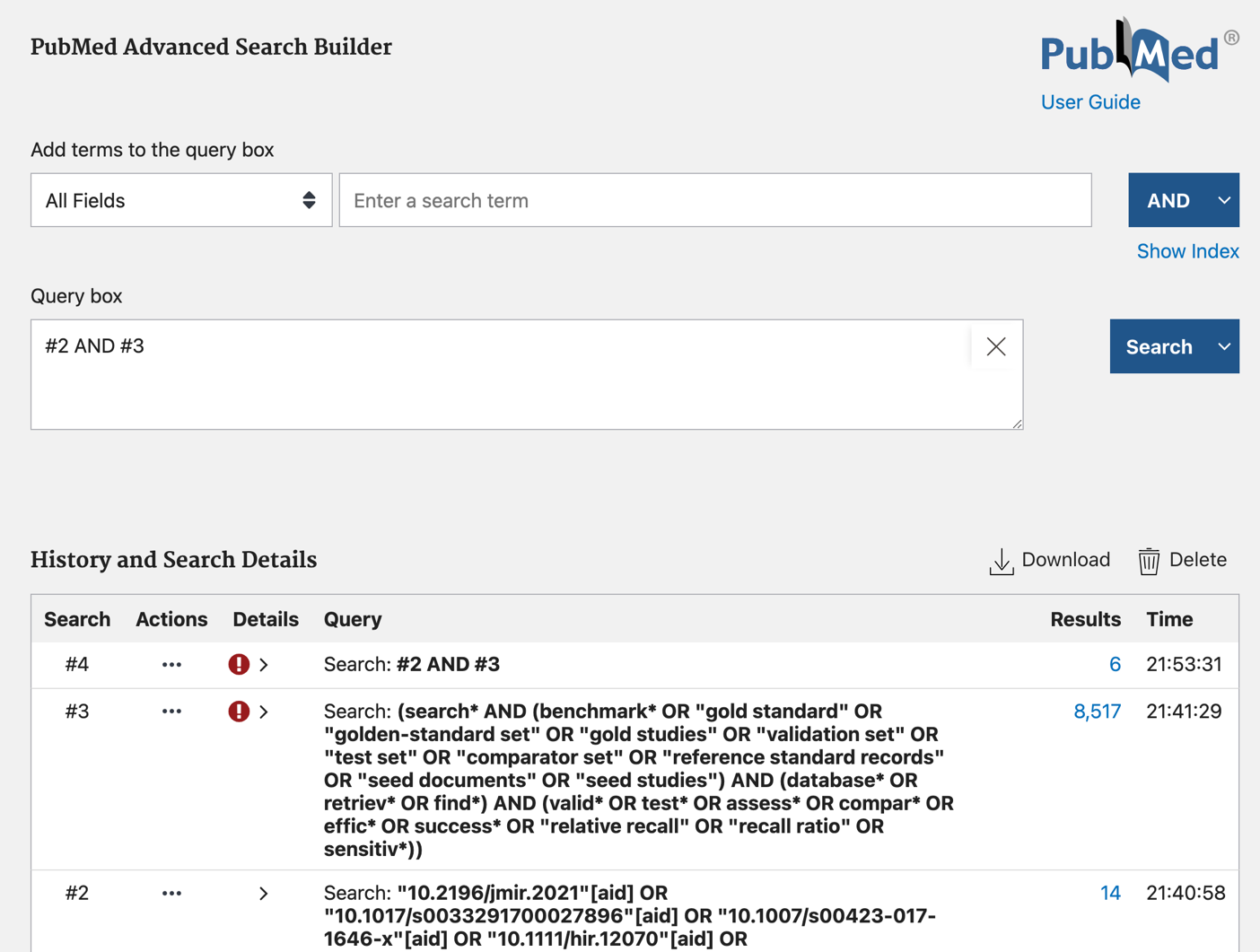


***Screenshot PubMed 6.***

*Combining results of two search queries using Advanced Search window Query box. The results appear in the History and Search Details section next to #4 Search.*

- 1. **Optional: If some benchmark records are missing, you can sometimes use "NOT" operator to see which one are missing (i.e. "(StringB) NOT (StringA)"):**

There is “Add with NOT” Action option for combining search results, but it is easy to combine search results manually by using their search ID numbers in the Advanced Search Query box, e.g., enter: *#2 NOT #4*. After executing the search, this will return the lit of benchmarks which were not found by the target search string.

**
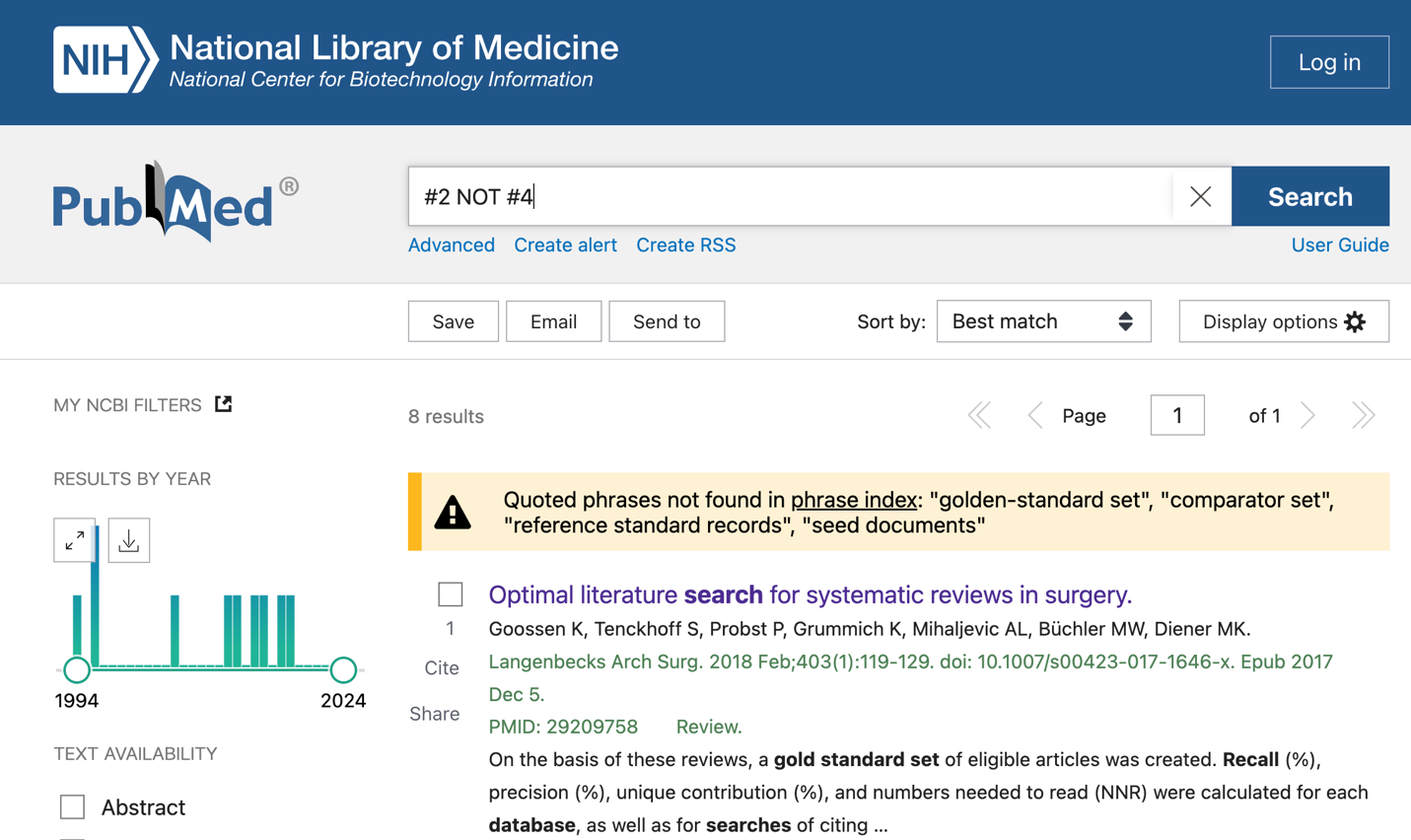
**

***Screenshot PubMed 7.***

*Manually combining results of two search queries using Boolean NOT operator with search strings numbers from search History.). Using “NOT” Boolean operator reveals the benchmark records not found by the target search string.*

### Step6

1. **Calculate sensitivity of the target search string.**
   1. **The number of overlapping records between the two search strings (target and benchmarking) is the number of the benchmark studies found by the evaluated target string (StringA). Thus, this number, divided by the total number of records retrieved by benchmarking string (StringB) is the estimate of your search sensitivity (SEN or relative recall):**

Since the overlap is 5 records, the sensitivity is 6/14 = 0.43, or 43%.

- 1. **Optional: You can iteratively modify your target search string (StringA). At every iteration, it is very easy to re-evaluate new target StringA against benchmarking StringB using the same method as above (combining the strings). When modifying your search string, you can start by reading through the titles and abstracts of these missed studies. Determine why the study was missed by your current search strategy. What terms are missing from your search string? If reasonable, add the missing search terms to your search (e.g., add terms that are synonyms of concepts already included in your search, expand proximity windows, adjust stemming, etc.). If there is no reasonable way to adjust the search to capture the study, make a note of this as a potential limitation of your search strategy. You can also see which benchmark papers were found:**

The list of results appears automatically when running the search string from the Query box. You can also use the link in the “Results” column in the search History.

- 1. **Recommended: Keep a good record of the search development and testing process (e.g., in a table), so you can document it transparently in your systematic review or protocol:**

PubMed allows downloading search History as a .csv file. You can also copy and paste History table for your record.

For example, table from a downloaded .csv file may look like this:

| ***Search***  ***number*** | ***Query*** |
| --- | --- |
| *5* | *#2 NOT #4* |
| *4* | *#2 AND #3* |
| *3* | *(search* AND (benchmark* OR "gold standard" OR "golden-standard set" OR "gold studies" OR "validation set" OR "test set" OR "comparator set" OR "reference standard records" OR "seed documents" OR "seed studies") AND (database* OR retriev* OR find*) AND (valid* OR test* OR assess* OR compar* OR effic* OR success* OR "relative recall" OR "recall ratio" OR sensitiv*))* |
| *2* | *"10.2196/jmir.2021"[aid] OR "10.1017/s0033291700027896"[aid] OR "10.1007/s00423-017-1646-x"[aid] OR "10.1111/hir.12070"[aid] OR "10.1016/j.jclinepi.2014.09.016"[aid] OR "10.1186/1472-6947-5-8"[aid] OR "10.1111/j.1471-1842.2004.00511.x"[aid] OR "10.3310/hta21690"[aid] OR "10.1002/14651858.MR000041.pub2" [aid] OR "10.1111/j.1528-1157.1996.tb00575.x"[aid] OR "10.1002/jrsm.40"[aid] OR "10.1186/1471-2288-6-33"[aid] OR "10.1136/bmj.313.7053.342"[aid] OR "10.1136/amiajnl-2012-001075"[aid]* |
| *1* | *"10.2196/jmir.2021"[aid] OR "10.1017/s0033291700027896"[aid] OR "10.1007/s00423-017-1646-x"[aid] OR "10.1111/hir.12070"[aid] OR "10.1016/j.jclinepi.2014.09.016"[aid] OR "10.1186/1472-6947-5-8"[aid] OR "10.1111/j.1471-1842.2004.00511.x"[aid] OR "10.3310/hta21690"[aid] OR "10.1002/14651858.MR000041.pub2" [aid] OR "10.1111/j.1528-1157.1996.tb00575.x"[aid] OR "10.1109/ACCESS.2019.2894679"[aid] OR "10.1002/jrsm.40"[aid] OR "10.1186/1471-2288-6-33"[aid] OR "10.1136/bmj.313.7053.342"[aid] OR "10.1136/amiajnl-2012-001075"[aid]* |

Same search history, manually saved in a table may look like this:

| **Date / database** | **Search nr** | **Search string** | **Search result / comment** |
| --- | --- | --- | --- |
| *8 January 2024* | 1 | *"10.2196/jmir.2021"[aid] OR "10.1017/s0033291700027896"[aid] OR "10.1007/s00423-017-1646-x"[aid] OR "10.1111/hir.12070"[aid] OR "10.1016/j.jclinepi.2014.09.016"[aid] OR "10.1186/1472-6947-5-8"[aid] OR "10.1111/j.1471-1842.2004.00511.x"[aid] OR "10.3310/hta21690"[aid] OR "10.1002/14651858.MR000041.pub2" [aid] OR "10.1111/j.1528-1157.1996.tb00575.x"[aid] OR "10.1109/ACCESS.2019.2894679"[aid] OR "10.1002/jrsm.40"[aid] OR "10.1186/1471-2288-6-33"[aid] OR "10.1136/bmj.313.7053.342"[aid] OR "10.1136/amiajnl-2012-001075"[aid]* | 14 /  14 out of 15 benchmark studies found, 8 benchmark studies not found |
|  | 2 | *"10.2196/jmir.2021"[aid] OR "10.1017/s0033291700027896"[aid] OR "10.1007/s00423-017-1646-x"[aid] OR "10.1111/hir.12070"[aid] OR "10.1016/j.jclinepi.2014.09.016"[aid] OR "10.1186/1472-6947-5-8"[aid] OR "10.1111/j.1471-1842.2004.00511.x"[aid] OR "10.3310/hta21690"[aid] OR "10.1002/14651858.MR000041.pub2" [aid] OR "10.1111/j.1528-1157.1996.tb00575.x"[aid] OR "10.1002/jrsm.40"[aid] OR "10.1186/1471-2288-6-33"[aid] OR "10.1136/bmj.313.7053.342"[aid] OR "10.1136/amiajnl-2012-001075"[aid]* | 14 / Removed missing benchmark studies from the string |
|  | 3 | *(search* AND (benchmark* OR "gold standard" OR "golden-standard set" OR "gold studies" OR "validation set" OR "test set" OR "comparator set" OR "reference standard records" OR "seed documents" OR "seed studies") AND (database* OR retriev* OR find*) AND (valid* OR test* OR assess* OR compar* OR effic* OR success* OR "relative recall" OR "recall ratio" OR sensitiv*))* | 8,518 / Target search string |
|  | 4 | *#2 AND #3* | 6 / Sensitivity is 6/14 = 0.43 (43%). Refine target search string |
|  | 5 | *#2 NOT #4* | See 8 Missing benchmark papers |

## Web of Science Core Collection

Web of Science (WoS; previously known as Web of Knowledge) is a subscription-based collection of multiple academic databases. Access to specific databases depends on the terms of an institutional subscription. Web of Science Core Collection, which includes six databases, is commonly used as a cross-disciplinary source of academic literature.


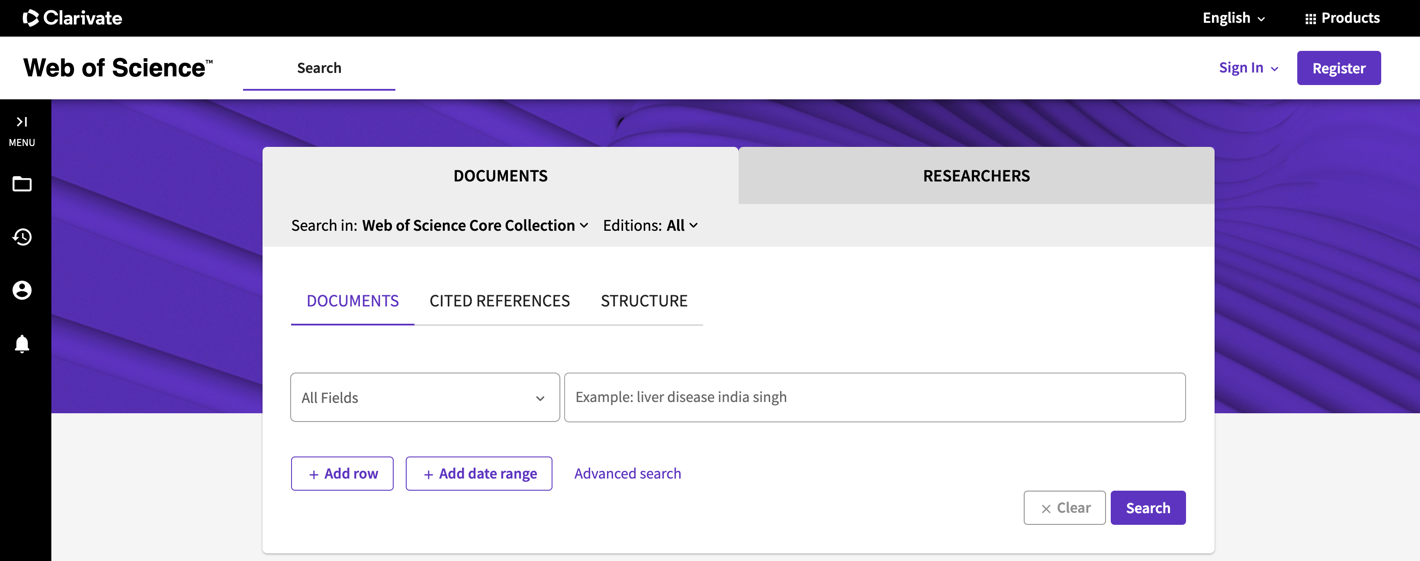


***Screenshot WoS 1.***

*Home window of WoS platform with Basic documents search box.*

### Step1

1. **Collect pre-known relevant studies (benchmarking set)**
   1. **Define a scope of your systematic review (or any systematic-like review using a systematic search approach), its inclusion and exclusion criteria:**

A hypothetical systematic-like review (a scoping review) on literature related to search string evaluations / benchmarking in systematic reviews and meta-analyses.

The scope of this scoping review in **PICo** framework:

- **P**opulation: systematic-like reviewers
- phenomena of **I**nterest: approaches and recommendations for benchmarking search strings
- **Co**ntext: systematic reviews and meta-analyses
  1. **Select the search sources to be used in your systematic review:**

Web of Science (WoS), Core Collection.

Link: <https://www.webofscience.com/>

Link to detailed description and help page: <https://images.webofknowledge.com/images/help/WOS/contents.html>

- 1. **Decide if search evaluation will be performed for one or more search sources, and which ones:**

PubMed only (for now).

- 1. **Gather a set of potential “benchmark” studies from diverse sources. Avoid using the databases you are planning to use as your systematic review search sources:**

The benchmark set of 15 relevant articles presented in **Table S1** has been assembled a priori from personal collections of articles, their reference lists and citations, similarity recommendations, and Google Scholar searches. The articles were pre-selected to represent diverse first authors, journals and disciplines. However, there was not restriction on study type, publication time or language, thus we include empirical, methodological and review articles, published anytime and in any language.

### Step2

1. **Search for the benchmark studies in a database you are evaluating:**
   1. **Create a benchmarking search string from all ID numbers (e.g., DOI) of the benchmark studies, using “OR” Boolean operator:**

WoS has a search field for DOI, which is specified as “DO” field tag. So, we can enter create a following search string using DOI benchmark article numbers:

*DO=(10.2196/jmir.2021 OR 10.1017/s0033291700027896 OR 10.1007/s00423-017-1646-x OR 10.1111/hir.12070 OR 10.1016/j.jclinepi.2014.09.016 OR 10.1186/1472-6947-5-8 OR 10.1111/j.1471-1842.2004.00511.x OR 10.3310/hta21690 OR 10.1002/14651858.MR000041.pub2 OR 10.1111/j.1528-1157.1996.tb00575.x OR 10.1109/ACCESS.2019.2894679 OR 10.1002/jrsm.40 OR 10.1186/1471-2288-6-33 OR 10.1136/bmj.313.7053.342 OR 10.1136/amiajnl-2012-001075)*

Note that this search string will work in the Basic Search mode (where DOI needs to be selected from the drop-down list in the left box and “DO=” removed from the search string that goes in the editable search box on the right) or we can use Advanced Search mode instead. Note that you should not use quotation marks around DOI numbers.

*
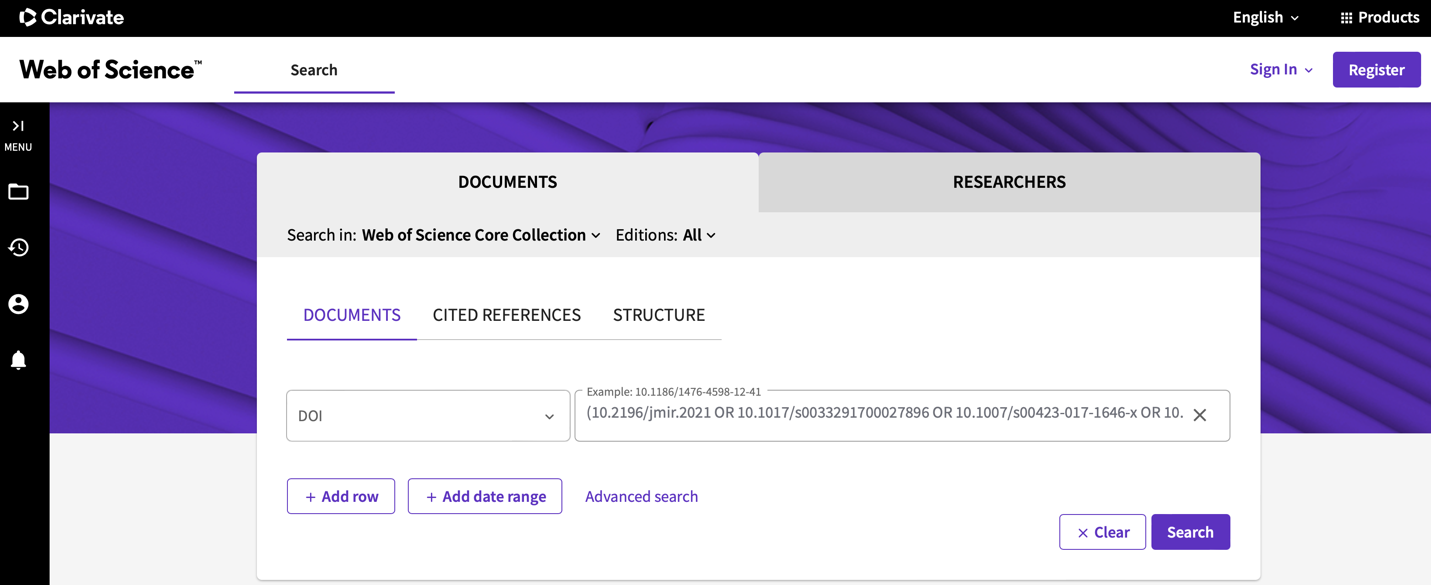
*

***Screenshot WoS 2.***

*Basic documents search window with an initial benchmarking search string pasted directly into editable box and DOI selected as a search field.*

*
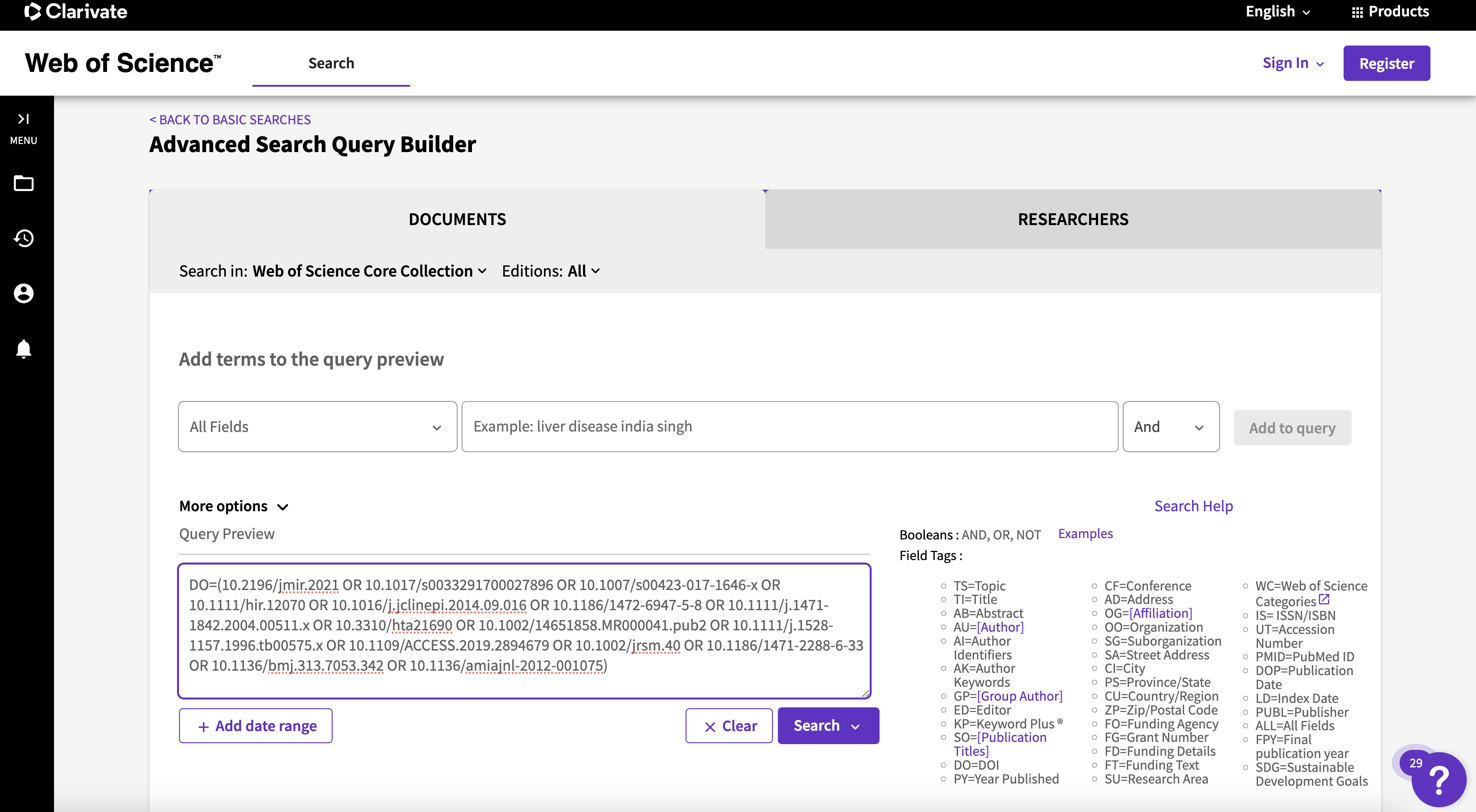
*

***Screenshot WoS 3.***

*Advanced Search Query Builder window with an initial benchmarking search string pasted directly into Query Preview box.*

- 1. **If a benchmark study is not found by its ID, it is either because of the true absence of the study record or incorrect/missing ID. Thus, for each incorrect/missing benchmark study run a search using its title or other identifying details (e.g., author, year). If found, check if the ID is correct and fix/replace the ID if needed, then search again by ID only. Pay attention to other potential issues, such as duplicated records, or single ID representing collections of works (e.g. conference abstracts book). Continue checking and refining this sub-step until you have a benchmark search string that retrieves all benchmark studies present in each database:**

Results of the initial search using 15 DOI as search terms: 12 articles found.

*
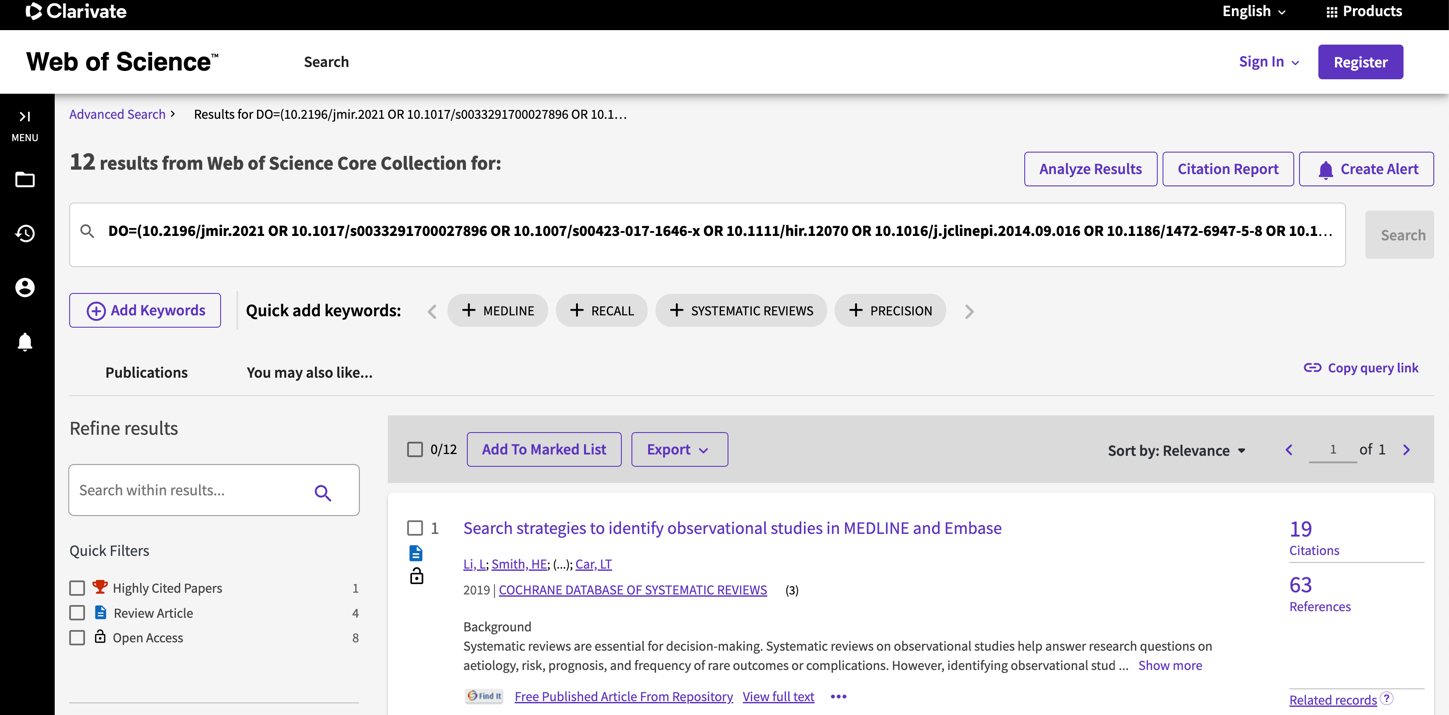
*

***Screenshot WoS 4.***

*Advanced Search results of running an initial benchmarking search string shown in the simple query box at the top of the window. The link to results will also appear in the search history table at the bottom of the Advanced Search Query Builder window.*

After identifying the missing benchmark articles, you can use Basic or Advanced Search mode for documents to run a title-based search for each one in turn. If found, you can check whether it has a correct DOI number or any ither ID number which could be used instead of a DOI. If a title search does not bring up the record, you can try searching by a combination of author name and year or journal. If you cannot find the article in a database, it is likely not there – i.e. it is a missing benchmark.

Missing benchmark articles and comments on the reasons:

1. Haynes et al. (2005) - 10.1186/1472-6947-5-8 – not in the database

2. Jenkins (2004) - 10.1111/j.1471-1842.2004.00511.x – not in the database

3. Sampson et al. (2006) - 10.1186/1471-2288-6-33 – not in the database

This search strings returns 12 (out of 15) benchmark articles which are indexed in WoS.

- 1. **Optional: Repeat for each database that will be used in search string evaluations.:**

Not applicable (single database evaluation).

### Step3

1. **Remove absent benchmark studies, keep the rest (i.e. customise your benchmarking set for each database).**
   1. **You can do it by simply removing IDs of the missing benchmark studies from a search string for a given database. This way you will have a clean benchmark search string with the IDs matching all benchmark studies present in a given database, which will make your search refinement and calculations easier:**

Revised benchmarking search string:

*DO=(10.2196/jmir.2021 OR 10.1017/s0033291700027896 OR 10.1007/s00423-017-1646-x OR 10.1111/hir.12070 OR 10.1016/j.jclinepi.2014.09.016 OR 10.3310/hta21690 OR 10.1002/14651858.MR000041.pub2 OR 10.1111/j.1528-1157.1996.tb00575.x OR 10.1109/ACCESS.2019.2894679 OR 10.1002/jrsm.40 OR 10.1136/bmj.313.7053.342 OR 10.1136/amiajnl-2012-001075)*

- 1. **Alternatively, you can just note which and how many benchmark studies are missing from a given database, and later adjust your search string refinements and calculations accordingly:**

Not applicable (removed the missing benchmarks).

- 1. **If relevant, set aside any benchmark studies that are absent from all of the databases you had planned to search. You can come back to these later to determine where they can be found (e.g. a grey literature source, an unindexed journal) and to determine if additional sources should be searched for your review.**

### Step4

1. **Run your target search string on a database.**
   1. **Typically, your target search string is a string composed by combining review scope-related terms (e.g., keywords, fixed expressions, controlled vocabulary, etc.) using Boolean (AND, OR) or other operators and field filters (e.g., which part of the bibliographic record to search, and any additional search limitations, like publication years or subject areas):**

Target search string (note the use of Boolean operators in capital letters):

*TS = (search* AND (benchmark* OR "gold standard" OR "golden-standard set" OR "gold studies" OR "validation set" OR "test set" OR "comparator set" OR "reference standard records" OR "seed documents" OR "seed studies") AND (database* OR retriev* OR find*) AND (valid* OR test* OR assess* OR compar* OR effic* OR success* OR "relative recall" OR "recall ratio" OR sensitiv*))*

If you don’t specify a search Field, this target search string will be interpreted across all searchable fields.

This preliminary and rough search string results in 10,945 hits.


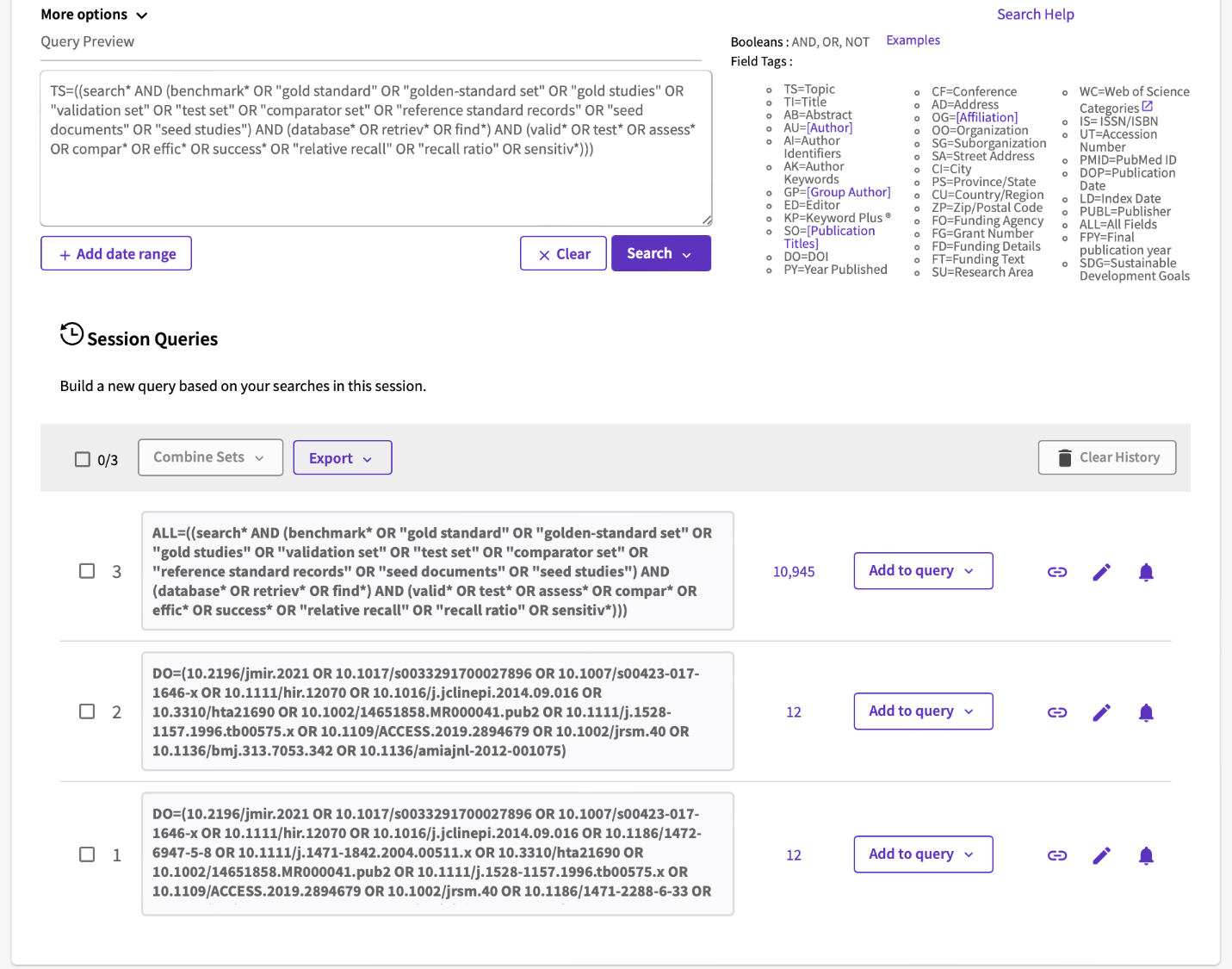


***Screenshot WoS 5.***

*Advanced Search window with an initial target search string in the search box and search history at the bottom (Session Queries) showing numbers of found records for each search string run.*

- 1. **The number of returned records (“hits”) can vary vastly and you should keep track of it for later target search string refinement.**

Although some graphical user interfaces of search engines allow saving or sharing search history, there is no way to annotate them on the go with custom comments (e.g., how and why a given search string has been changed or other issues). It is usually easy enough to copy and paste search history into an independent document (e.g., a spreadsheet) and add comments and notes. This spreadsheet can be used for multiple search sessions, including search dates and outcomes, list of benchmarking articles and sensitivity estimates.

### Step5

1. **Find the benchmark studies among the target search results.**
   1. **This step tests the overlap between records retrieved by the target search string and the benchmark set. Here we can simply combine the two strings. For example, if StringA is a target search string to be evaluated for recall, and if StringB retrieves bibliographic records for all benchmark studies by using their ID numbers, then running a combined search sting in a format “(StringA) AND (StringB)” will retrieve the records that overlap between the two:**

This can be done by going to Advanced Search window. It is easiest to use search numbers, as stored in the search history, to combine the already executed strings, e.g., by entering in the Query box:

*#2 AND #3*

Alternatively, you could use “Add to query” boxes next to each saved query in History and Search Details section. From the drop-down menus within an “Add to query” select the appropriate Boolean operator to use. Clear old query before adding further actions, if needed.

The overlap between the benchmarking search string and the target search string is 4, and the found benchmark studies are:

- - - Gossen et al. (2018)
    - Harbour et al. (2014)
    - Li et al. (2019)
    - Wilczynski et al. (2013)


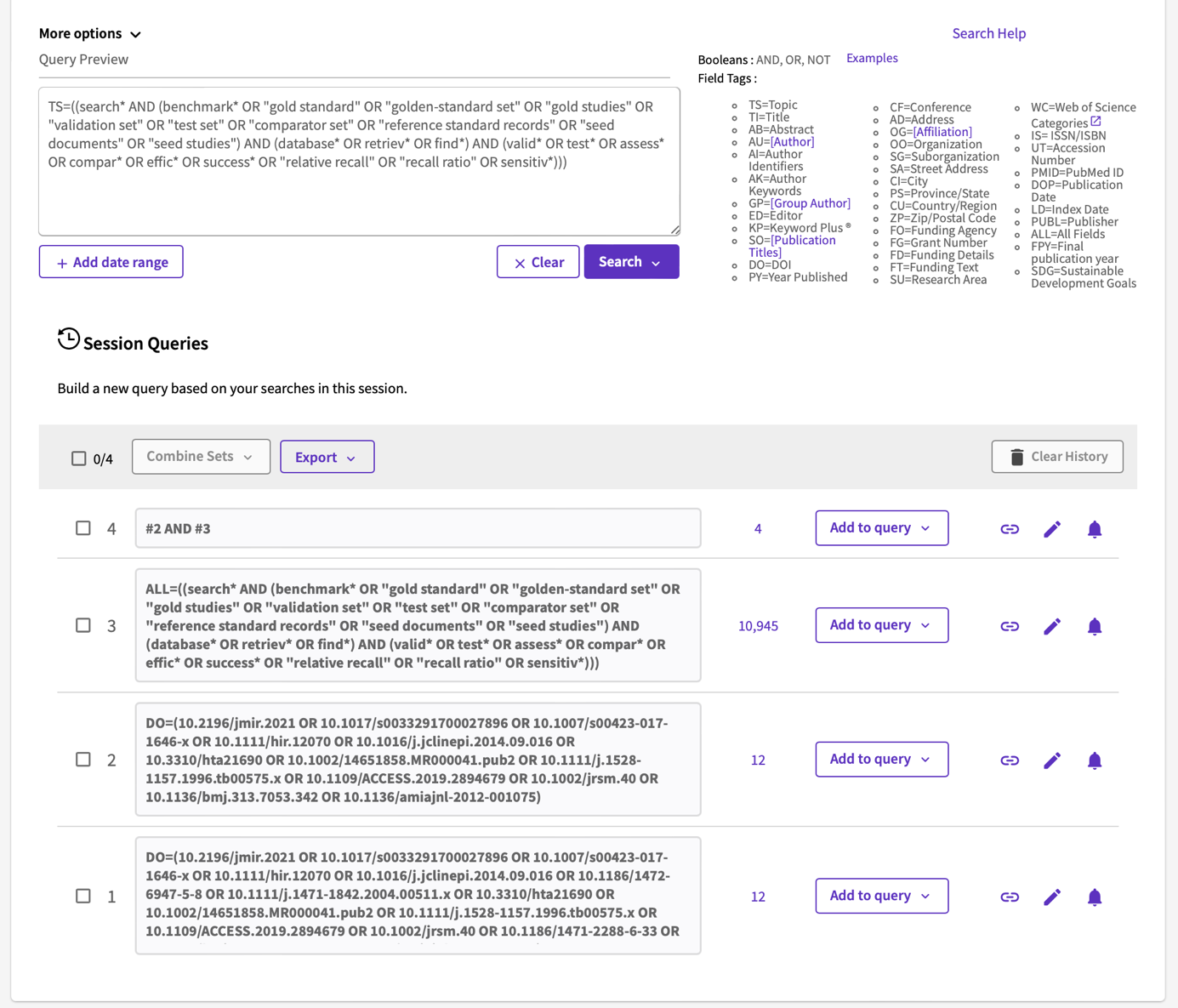


***Screenshot WoS 6.***

*Combining results of two search queries using Advanced Search window. The results appear in the search history (Session Queries) as search number 4.*

- 1. **Optional: If some benchmark records are missing, you can sometimes use "NOT" operator to see which one are missing (i.e. "(StringB) NOT (StringA)"):**

There is “Combine with NOT” option for combining search results using tick-boxes, but it is easy to combine search results manually by using their search numbers in the Advanced Search Query Preview box, e.g., enter: *#2 NOT #4*. After executing the search, this will return the lit of benchmarks which were not found by the target search string.

**
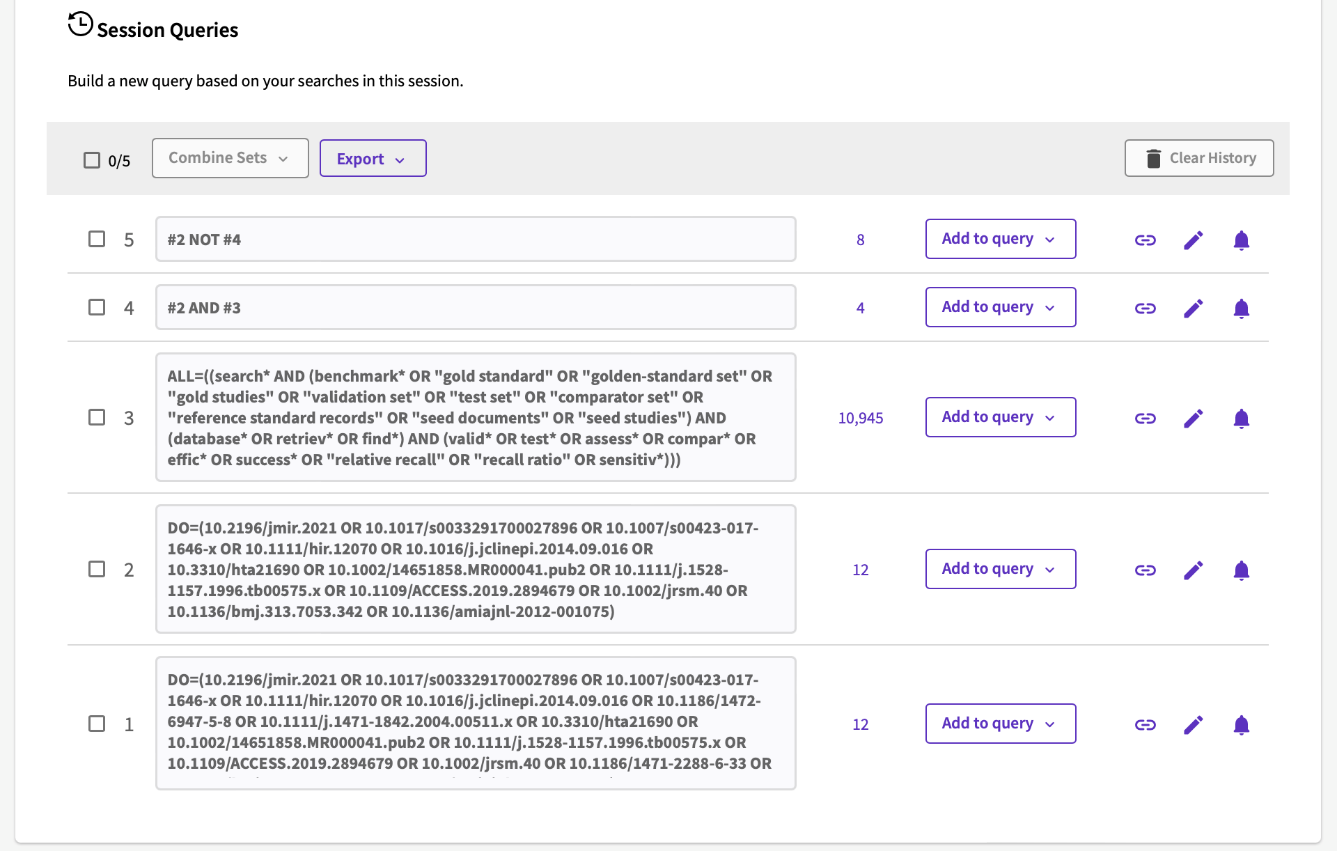
**

***Screenshot WoS 7.***

*Manually combining results of two search queries using Boolean NOT operator with search strings numbers from search History.). Using “NOT” Boolean operator reveals the benchmark records not found by the target search string.*

### Step6

1. **Calculate sensitivity of the target search string.**
   1. **The number of overlapping records between the two search strings (target and benchmarking) is the number of the benchmark studies found by the evaluated target string (StringA). Thus, this number, divided by the total number of records retrieved by benchmarking string (StringB) is the estimate of your search sensitivity (SEN or relative recall):**

Since the overlap is 4 records, the sensitivity is 4/12 = 0.33, or 33%.

- 1. **Optional: You can iteratively modify your target search string (StringA). At every iteration, it is very easy to re-evaluate new target StringA against benchmarking StringB using the same method as above (combining the strings). When modifying your search string, you can start by reading through the titles and abstracts of these missed studies. Determine why the study was missed by your current search strategy. What terms are missing from your search string? If reasonable, add the missing search terms to your search (e.g., add terms that are synonyms of concepts already included in your search, expand proximity windows, adjust stemming, etc.). If there is no reasonable way to adjust the search to capture the study, make a note of this as a potential limitation of your search strategy. You can also see which benchmark papers were found:**

The list of results appears automatically when running the search string from the Query box. You can also use the links in the search History, by clicking on the number of the records found by each search string.

- 1. **Recommended: Keep a good record of the search development and testing process (e.g., in a table), so you can document it transparently in your systematic review or protocol:**

WoS allows downloading search History in three different formats: as a plain text file, pdf, or Excel file. You can also copy and paste History table for your record.

For example, table from a downloaded Excel file may look like this:

| **Entitlements** | **#** | **Search Query** | **Database** | **Results** | **Date Run** |
| --- | --- | --- | --- | --- | --- |
| - WOS.IC: 1993 to 2024 - WOS.CCR: 1985 to 2024 - WOS.SCI: 1900 to 2024 - WOS.AHCI: 1975 to 2024 - WOS.ESCI: 2005 to 2024 - WOS.ISTP: 1990 to 2024 - WOS.SSCI: 1900 to 2024 - WOS.ISSHP: 1990 to 2024 | 1 | *DO=(10.2196/jmir.2021 OR 10.1017/s0033291700027896 OR 10.1007/s00423-017-1646-x OR 10.1111/hir.12070 OR 10.1016/j.jclinepi.2014.09.016 OR 10.1186/1472-6947-5-8 OR 10.1111/j.1471-1842.2004.00511.x OR 10.3310/hta21690 OR 10.1002/14651858.MR000041.pub2 OR 10.1111/j.1528-1157.1996.tb00575.x OR 10.1109/ACCESS.2019.2894679 OR 10.1002/jrsm.40 OR 10.1186/1471-2288-6-33 OR 10.1136/bmj.313.7053.342 OR 10.1136/amiajnl-2012-001075)* | Web of Science Core Collection | 12 | Wed Jan 10 2024 08:08:52 GMT+1100 (Australian Eastern Daylight Time) |
| - WOS.IC: 1993 to 2024 - WOS.CCR: 1985 to 2024 - WOS.SCI: 1900 to 2024 - WOS.AHCI: 1975 to 2024 - WOS.ESCI: 2005 to 2024 - WOS.ISTP: 1990 to 2024 - WOS.SSCI: 1900 to 2024 - WOS.ISSHP: 1990 to 2024 | 2 | *DO=(10.2196/jmir.2021 OR 10.1017/s0033291700027896 OR 10.1007/s00423-017-1646-x OR 10.1111/hir.12070 OR 10.1016/j.jclinepi.2014.09.016 OR 10.3310/hta21690 OR 10.1002/14651858.MR000041.pub2 OR 10.1111/j.1528-1157.1996.tb00575.x OR 10.1109/ACCESS.2019.2894679 OR 10.1002/jrsm.40 OR 10.1136/bmj.313.7053.342 OR 10.1136/amiajnl-2012-001075)* | Web of Science Core Collection | 12 | Wed Jan 10 2024 08:09:08 GMT+1100 (Australian Eastern Daylight Time) |
| - WOS.IC: 1993 to 2024 - WOS.CCR: 1985 to 2024 - WOS.SCI: 1900 to 2024 - WOS.AHCI: 1975 to 2024 - WOS.ESCI: 2005 to 2024 - WOS.ISTP: 1990 to 2024 - WOS.SSCI: 1900 to 2024 - WOS.ISSHP: 1990 to 2024 | 3 | *ALL=((search* AND (benchmark* OR "gold standard" OR "golden-standard set" OR "gold studies" OR "validation set" OR "test set" OR "comparator set" OR "reference standard records" OR "seed documents" OR "seed studies") AND (database* OR retriev* OR find*) AND (valid* OR test* OR assess* OR compar* OR effic* OR success* OR "relative recall" OR "recall ratio" OR sensitiv*)))* | Web of Science Core Collection | 10945 | Wed Jan 10 2024 08:10:26 GMT+1100 (Australian Eastern Daylight Time) |
| - WOS.IC: 1993 to 2024 - WOS.CCR: 1985 to 2024 - WOS.SCI: 1900 to 2024 - WOS.AHCI: 1975 to 2024 - WOS.ESCI: 2005 to 2024 - WOS.ISTP: 1990 to 2024 - WOS.SSCI: 1900 to 2024 - WOS.ISSHP: 1990 to 2024 | 4 | *#2 AND #3* | Web of Science Core Collection | 4 | Wed Jan 10 2024 08:14:42 GMT+1100 (Australian Eastern Daylight Time) |
| - WOS.IC: 1993 to 2024 - WOS.CCR: 1985 to 2024 - WOS.SCI: 1900 to 2024 - WOS.AHCI: 1975 to 2024 - WOS.ESCI: 2005 to 2024 - WOS.ISTP: 1990 to 2024 - WOS.SSCI: 1900 to 2024 - WOS.ISSHP: 1990 to 2024 | 5 | *#2 NOT #4* | Web of Science Core Collection | 8 | Wed Jan 10 2024 08:17:26 GMT+1100 (Australian Eastern Daylight Time) |

Same search history, manually saved in a table may look like this:

| **Date / database** | **Search nr** | **Search string** | **Search result / comment** |
| --- | --- | --- | --- |
| 10 January 2024  Web of Science Core Collection:  - WOS.IC: 1993 to 2024 - WOS.CCR: 1985 to 2024 - WOS.SCI: 1900 to 2024 - WOS.AHCI: 1975 to 2024 - WOS.ESCI: 2005 to 2024 - WOS.ISTP: 1990 to 2024 - WOS.SSCI: 1900 to 2024 - WOS.ISSHP: 1990 to 2024 | 1 | *DO=(10.2196/jmir.2021 OR 10.1017/s0033291700027896 OR 10.1007/s00423-017-1646-x OR 10.1111/hir.12070 OR 10.1016/j.jclinepi.2014.09.016 OR 10.1186/1472-6947-5-8 OR 10.1111/j.1471-1842.2004.00511.x OR 10.3310/hta21690 OR 10.1002/14651858.MR000041.pub2 OR 10.1111/j.1528-1157.1996.tb00575.x OR 10.1109/ACCESS.2019.2894679 OR 10.1002/jrsm.40 OR 10.1186/1471-2288-6-33 OR 10.1136/bmj.313.7053.342 OR 10.1136/amiajnl-2012-001075)* | 12 /  12 out of 15 benchmark studies found, 3 benchmark studies not found |
|  | 2 | *DO=(10.2196/jmir.2021 OR 10.1017/s0033291700027896 OR 10.1007/s00423-017-1646-x OR 10.1111/hir.12070 OR 10.1016/j.jclinepi.2014.09.016 OR 10.3310/hta21690 OR 10.1002/14651858.MR000041.pub2 OR 10.1111/j.1528-1157.1996.tb00575.x OR 10.1109/ACCESS.2019.2894679 OR 10.1002/jrsm.40 OR 10.1136/bmj.313.7053.342 OR 10.1136/amiajnl-2012-001075)* | 12 / Removed missing benchmark studies from the string |
|  | 3 | *ALL=((search* AND (benchmark* OR "gold standard" OR "golden-standard set" OR "gold studies" OR "validation set" OR "test set" OR "comparator set" OR "reference standard records" OR "seed documents" OR "seed studies") AND (database* OR retriev* OR find*) AND (valid* OR test* OR assess* OR compar* OR effic* OR success* OR "relative recall" OR "recall ratio" OR sensitiv*)))* | 10,945 / Target search string |
|  | 4 | *#2 AND #3* | 4 / Sensitivity is 6/12 = 0.33 (33%). Refine target search string |
|  | 5 | *#2 NOT #4* | 8 Missing benchmark papers |

## Scopus

Scopus is a subscription-based cross-disciplinary academic database. It mostly indexes publications from academic journals deemed to be of sufficiently high quality based on bibliometric evaluations. Scopus is in the process of updating its graphical user interface, so it has an older and a newer version of the interface currently in use (they are similar and the workflow will work on both).


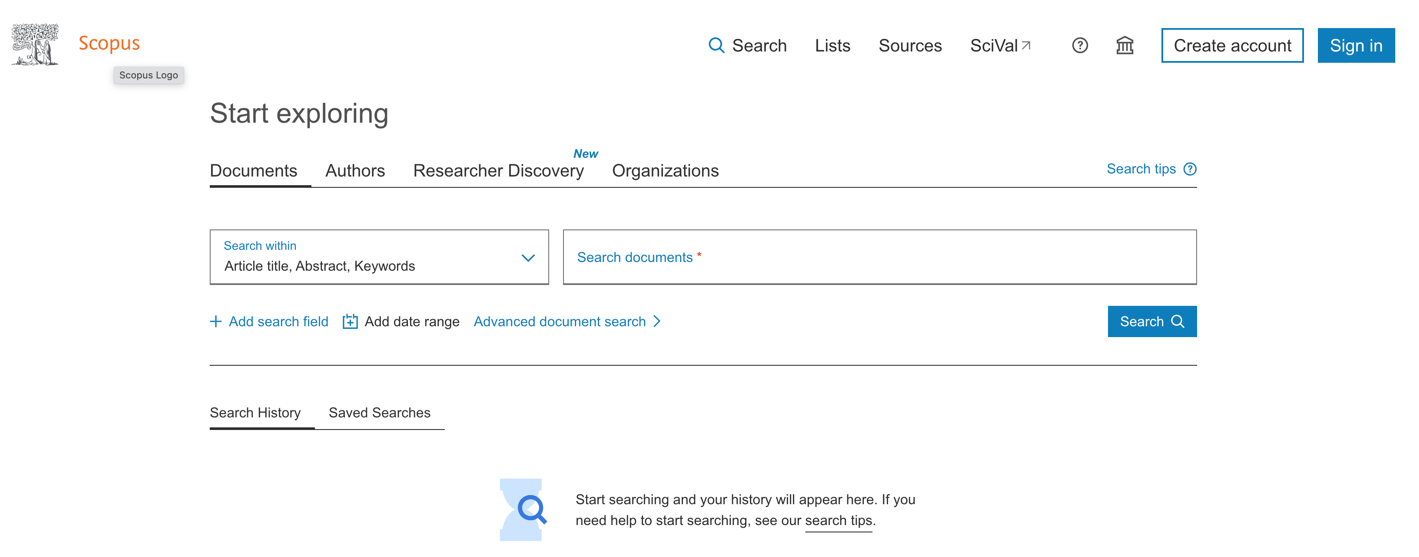


***Screenshot Scopus 1.***

*Home window of Scopus platform with Basic documents search box.*

### Step1

1. **Collect pre-known relevant studies (benchmarking set)**
   1. **Define a scope of your systematic review (or any systematic-like review using a systematic search approach), its inclusion and exclusion criteria:**

A hypothetical systematic-like review (a scoping review) on literature related to search string evaluations / benchmarking in systematic reviews and meta-analyses.

The scope of this scoping review in **PICo** framework:

- **P**opulation: systematic-like reviewers
- phenomena of **I**nterest: approaches and recommendations for benchmarking search strings
- **Co**ntext: systematic reviews and meta-analyses
  1. **Select the search sources to be used in your systematic review:**

Scopus.

Link: <https://www.scopus.com>

Link to detailed description and help page:

<https://schema.elsevier.com/dtds/document/bkapi/search/SCOPUSSearchTips.htm>

- 1. **Decide if search evaluation will be performed for one or more search sources, and which ones:**

Scopus only (for now).

- 1. **Gather a set of potential “benchmark” studies from diverse sources. Avoid using the databases you are planning to use as your systematic review search sources:**

The benchmark set of 15 relevant articles presented in **Table S1** has been assembled a priori from personal collections of articles, their reference lists and citations, similarity recommendations, and Google Scholar searches. The articles were pre-selected to represent diverse first authors, journals and disciplines. However, there was not restriction on study type, publication time or language, thus we include empirical, methodological and review articles, published anytime and in any language.

### Step2

1. **Search for the benchmark studies in a database you are evaluating:**
   1. **Create a benchmarking search string from all ID numbers (e.g., DOI) of the benchmark studies, using “OR” Boolean operator:**

Scopus has a search field for DOI, which is specified as “DOI” field tag. So, we can enter create a following search string using DOI benchmark article numbers:

*DOI(10.2196/jmir.2021) OR DOI(10.1017/s0033291700027896) OR DOI(10.1007/s00423-017-1646-x) OR DOI(10.1111/hir.12070) OR DOI(10.1016/j.jclinepi.2014.09.016) OR DOI(10.1186/1472-6947-5-8) OR DOI(10.1111/j.1471-1842.2004.00511.x) OR DOI(10.3310/hta21690) OR DOI(10.1002/14651858.mr000041.pub2) OR DOI(10.1111/j.1528-1157.1996.tb00575.x) OR DOI(10.1109/access.2019.2894679) OR DOI(10.1002/jrsm.40) OR DOI(10.1186/1471-2288-6-33) OR DOI(10.1136/bmj.313.7053.342) OR DOI(10.1136/amiajnl-2012-001075)*

Note that this search string will not work in the Basic Search mode unless you enter each Doi in a separates search box with Doi selected as a search field for each. It is convenient to use Advanced search mode instead, where you can copy and paste whole search string. Note that you should not use quotation marks around DOI numbers when they are enclosed in their own round brackets.

*
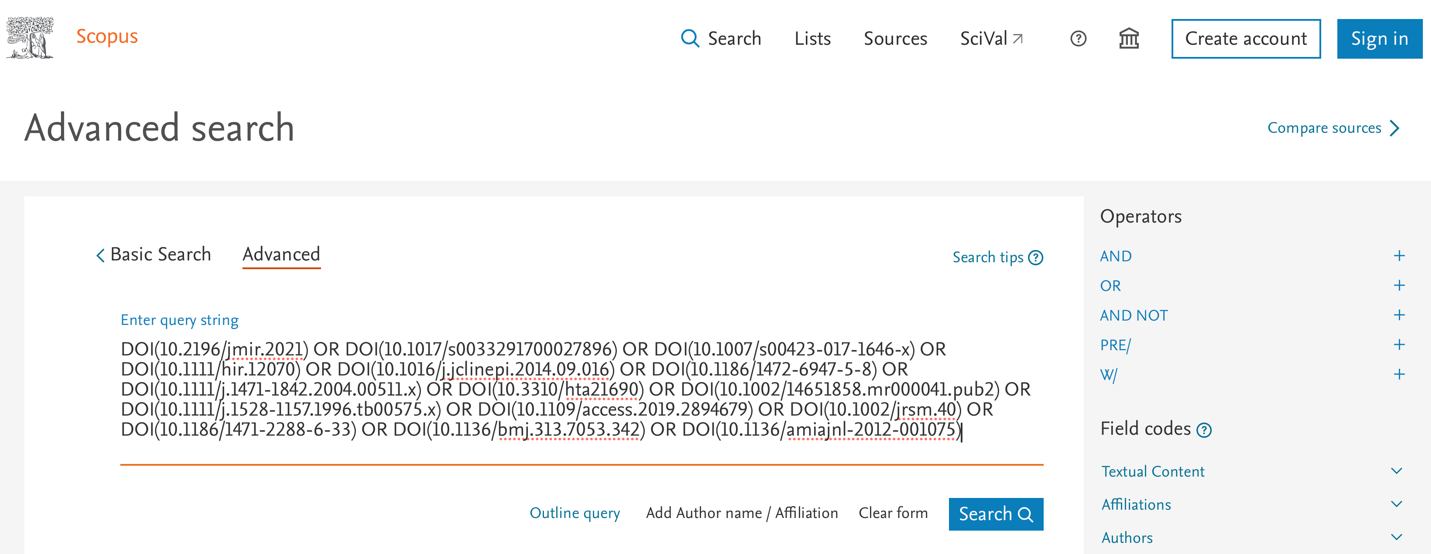
*

***Screenshot Scopus 2.***

*Advanced search window with an initial benchmarking search string pasted directly into query box.*

- 1. **If a benchmark study is not found by its ID, it is either because of the true absence of the study record or incorrect/missing ID. Thus, for each incorrect/missing benchmark study run a search using its title or other identifying details (e.g., author, year). If found, check if the ID is correct and fix/replace the ID if needed, then search again by ID only. Pay attention to other potential issues, such as duplicated records, or single ID representing collections of works (e.g. conference abstracts book). Continue checking and refining this sub-step until you have a benchmark search string that retrieves all benchmark studies present in each database:**

Results of the initial search using 15 DOI as search terms: 14 articles found.

*
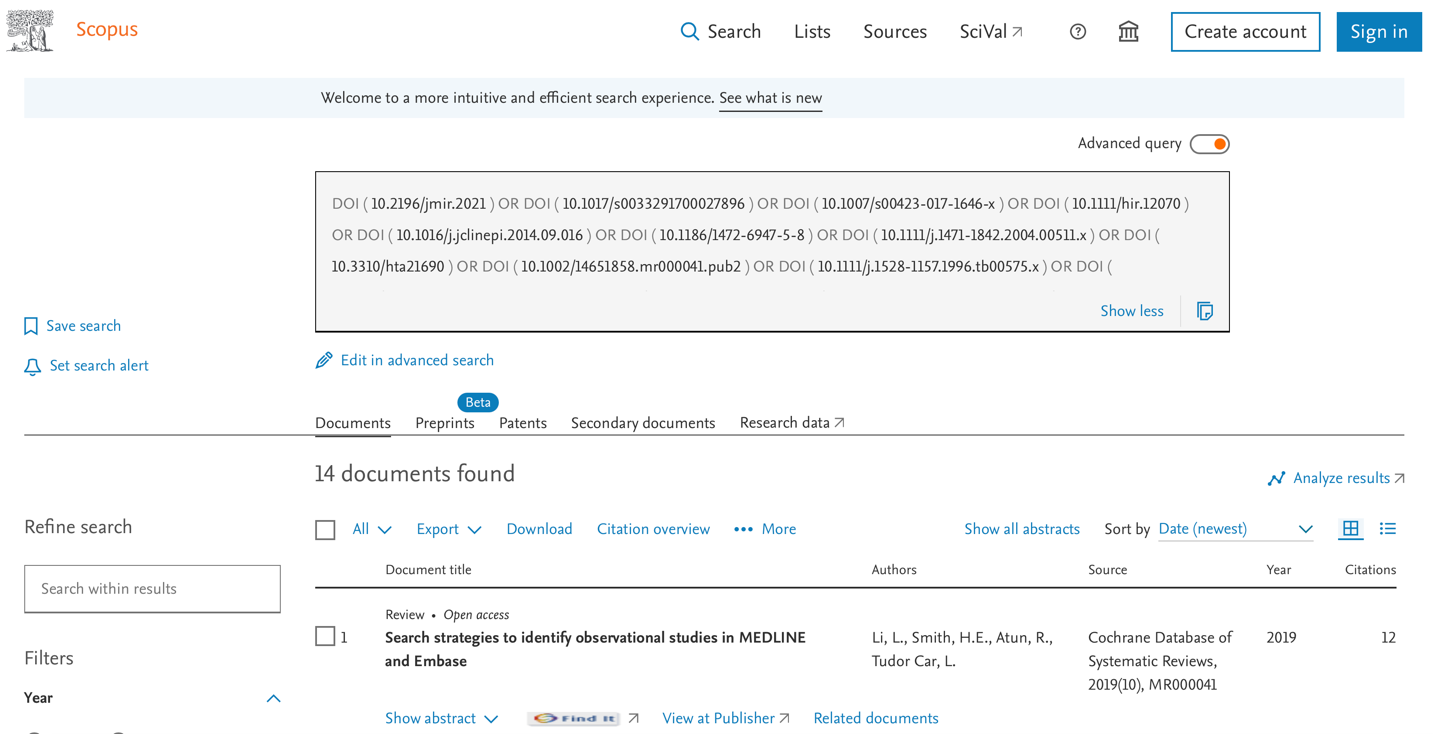
*

***Screenshot Scopus 3.***

*Advanced search results of running an initial benchmarking search string shown in the simple query box at the top of the window. The link to results will also appear in the search history table at the bottom of the Advanced search window.*

After identifying the missing benchmark articles, you can use Basic or Advanced Search mode to run a title-based search for each one in turn. If found, you can check whether it has a correct DOI number or any ither ID number which could be used instead of a DOI. If a title search does not bring up the record, you can try searching by a combination of author name and year or journal. If you cannot find the article in a database, it is likely not there – i.e. it is a missing benchmark.

Missing benchmark articles and comments on the reasons:

1. Jenkins et al. (2004) - 10.1111/j.1471-1842.2004.00511.x – searched by title and found. This article has no DOI indexed in this database, but has PubMed ID number (PMID), which can be used instead (2004165685).

Revised benchmarking search string:

*DOI(10.2196/jmir.2021) OR DOI(10.1017/s0033291700027896) OR DOI(10.1007/s00423-017-1646-x) OR DOI(10.1111/hir.12070) OR DOI(10.1016/j.jclinepi.2014.09.016) OR DOI(10.1186/1472-6947-5-8) OR DOI(10.3310/hta21690) OR DOI(10.1002/14651858.mr000041.pub2) OR DOI(10.1111/j.1528-1157.1996.tb00575.x) OR DOI(10.1109/access.2019.2894679) OR DOI(10.1002/jrsm.40) OR DOI(10.1186/1471-2288-6-33) OR DOI(10.1136/bmj.313.7053.342) OR DOI(10.1136/amiajnl-2012-001075) OR PMID ( 2004165685)*

This search strings returns 15 (out of 15) benchmark articles which are indexed in Scopus.

- 1. **Optional: Repeat for each database that will be used in search string evaluations.:**

Not applicable (single database evaluation).

### Step3

1. **Remove absent benchmark studies, keep the rest (i.e. customise your benchmarking set for each database).**
   1. **You can do it by simply removing IDs of the missing benchmark studies from a search string for a given database. This way you will have a clean benchmark search string with the IDs matching all benchmark studies present in a given database, which will make your search refinement and calculations easier:**

Not applicable – the benchmarking search string finds all benchmark papers.

- 1. **Alternatively, you can just note which and how many benchmark studies are missing from a given database, and later adjust your search string refinements and calculations accordingly:**

Not applicable – the benchmarking search string finds all benchmark papers.

- 1. **If relevant, set aside any benchmark studies that are absent from all of the databases you had planned to search. You can come back to these later to determine where they can be found (e.g. a grey literature source, an unindexed journal) and to determine if additional sources should be searched for your review.**

### Step4

1. **Run your target search string on a database.**
   1. **Typically, your target search string is a string composed by combining review scope-related terms (e.g., keywords, fixed expressions, controlled vocabulary, etc.) using Boolean (AND, OR) or other operators and field filters (e.g., which part of the bibliographic record to search, and any additional search limitations, like publication years or subject areas):**

Target search string (note the use of Boolean operators in capital letters) for searching titles, abstracts, and keywords:

*TITLE-ABS-KEY(search* AND (benchmark* OR "gold standard" OR "golden-standard set" OR "gold studies" OR "validation set" OR "test set" OR "comparator set" OR "reference standard records" OR "seed documents" OR "seed studies") AND (database* OR retriev* OR find*) AND (valid* OR test* OR assess* OR compar* OR effic* OR success* OR "relative recall" OR "recall ratio" OR sensitiv*))*

If you don’t specify a search Field, this target search string will be interpreted across all searchable fields.

This preliminary and rough search string results in 13,819 hits.


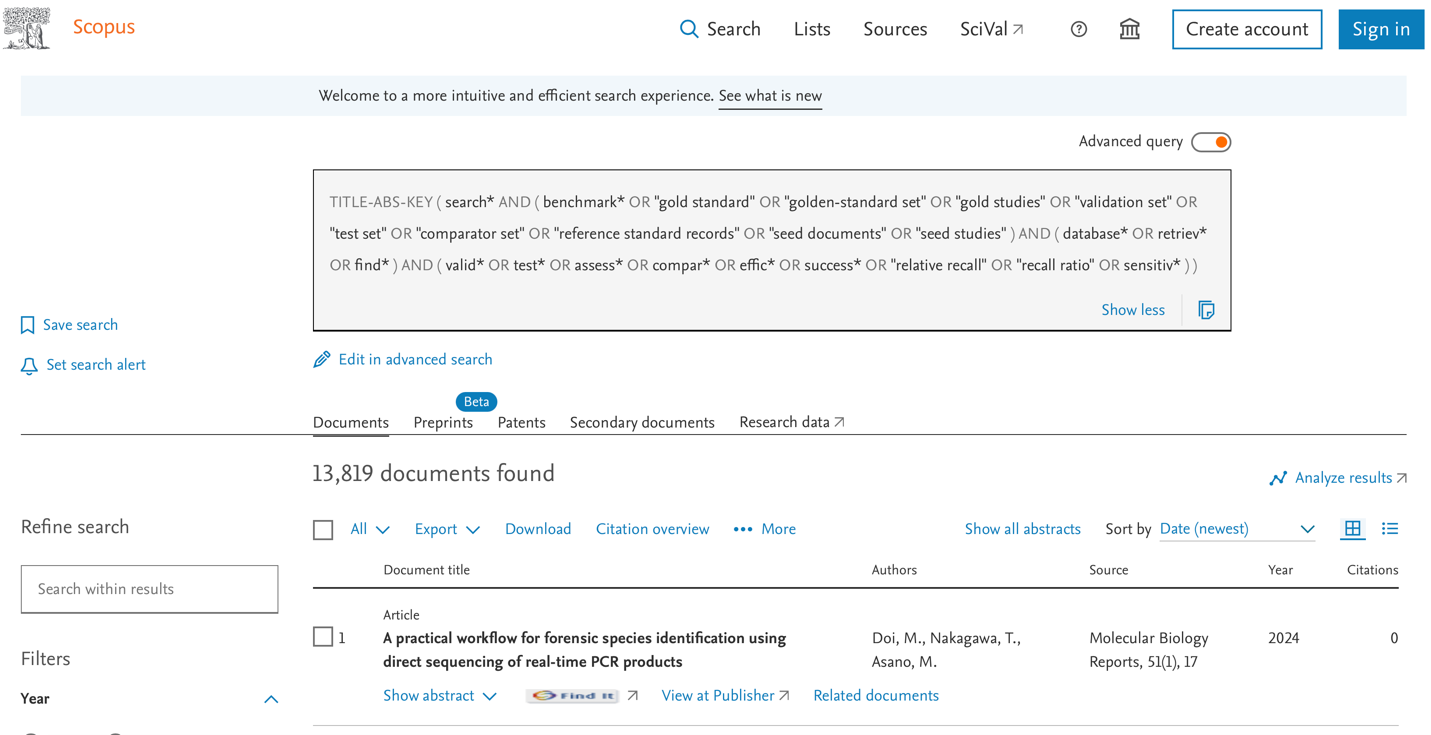


***Screenshot Scopus 4.***

*Advanced search window with an initial target search string in the search box and found records at the bottom.*

- 1. **The number of returned records (“hits”) can vary vastly and you should keep track of it for later target search string refinement.**

Although some graphical user interfaces of search engines allow saving or sharing search history, there is no way to annotate them on the go with custom comments (e.g., how and why a given search string has been changed or other issues). It is usually easy enough to copy and paste search history into an independent document (e.g., a spreadsheet) and add comments and notes. This spreadsheet can be used for multiple search sessions, including search dates and outcomes, list of benchmarking articles and sensitivity estimates.

### Step5

1. **Find the benchmark studies among the target search results.**
   1. **This step tests the overlap between records retrieved by the target search string and the benchmark set. Here we can simply combine the two strings. For example, if StringA is a target search string to be evaluated for recall, and if StringB retrieves bibliographic records for all benchmark studies by using their ID numbers, then running a combined search sting in a format “(StringA) AND (StringB)” will retrieve the records that overlap between the two:**

This can be done by going to Advanced search window. It is easiest to use search numbers, as stored in the search history, to combine the already executed strings, e.g., by using the “Combine queries” box above the search history table. In our example the combined search string (query) will be expressed as: *#2 AND #3.*

The overlap between the benchmarking search string and the target search string is 5, and the found benchmark studies are:

- - - Harbour et al. (2014)
    - Jenkins (2004)
    - Li et al. (2019)
    - Sampson et al. (2006)
    - Wilczynski et al. (2013)


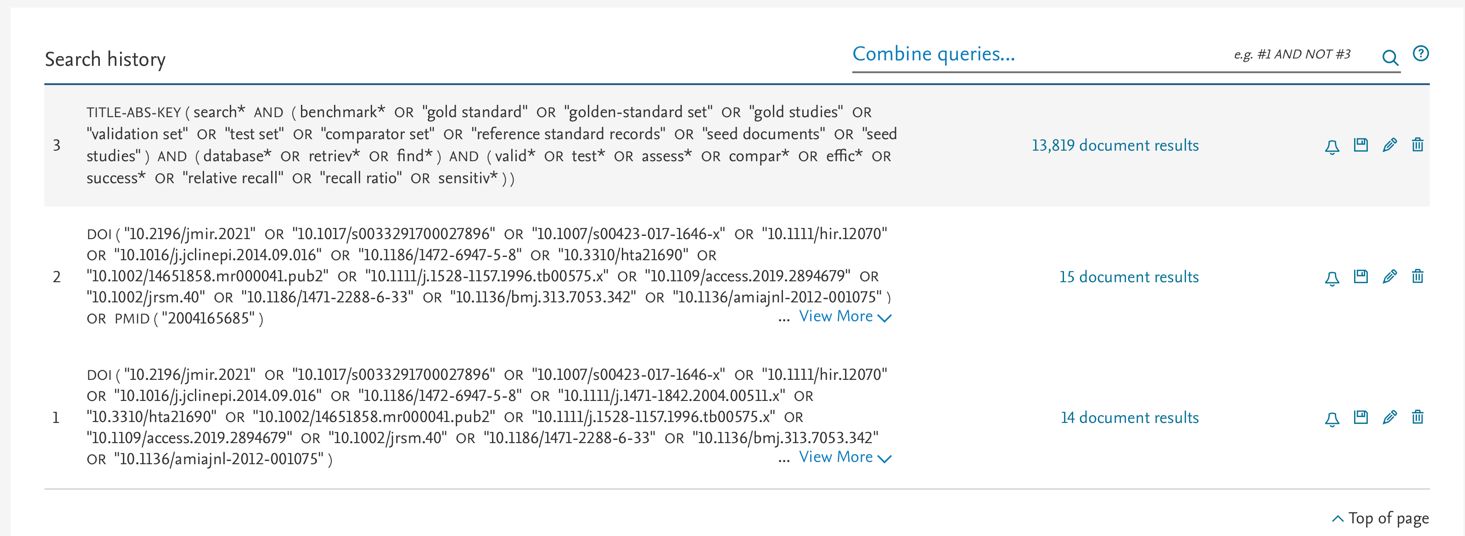


***Screenshot Scopus 5.***

*Search history at the bottom of the Advanced search window. There is a “Combine queries” box in the top right corner of the Search history table in the older version of the user interface.*

*
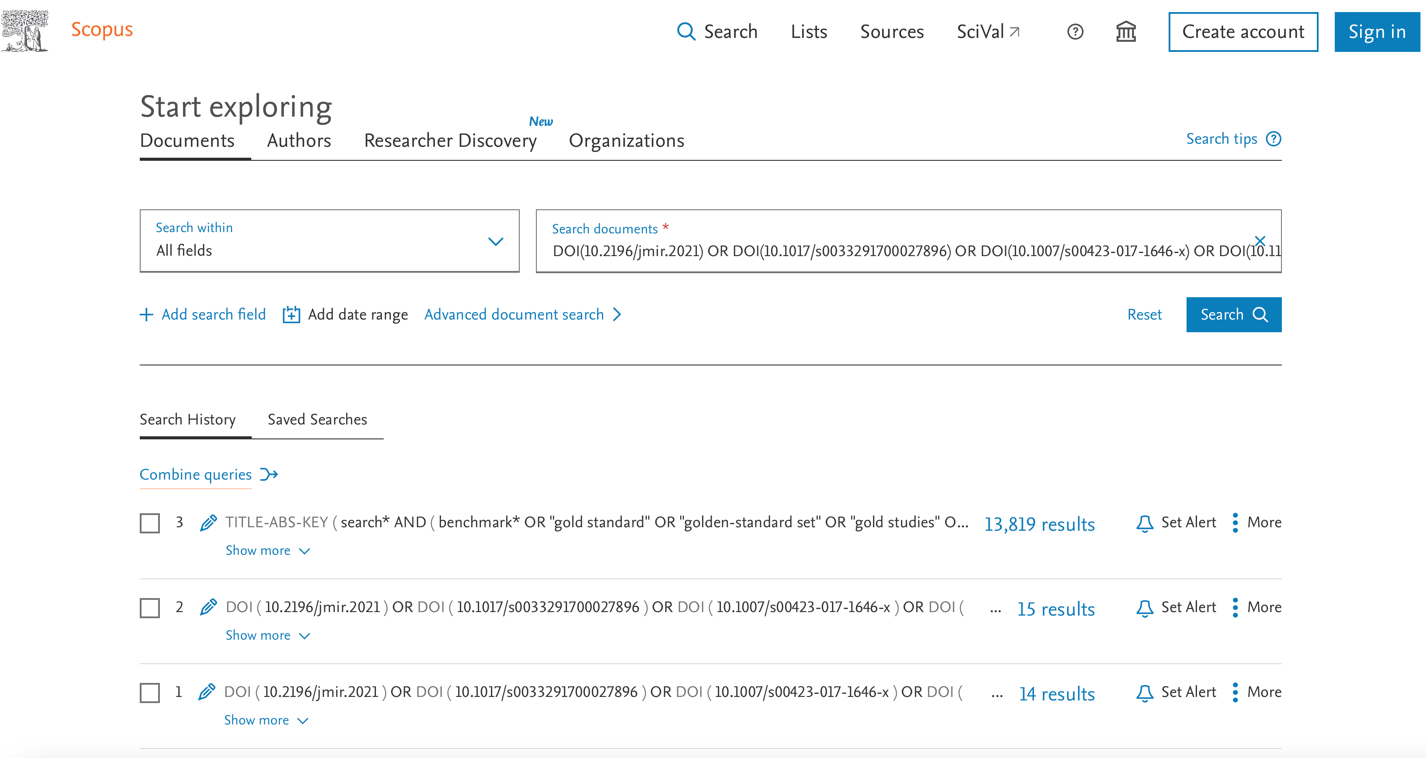
*

***Screenshot Scopus 6.***

*Search history at the bottom of the Advanced search window. There is a “Combine queries” box in the top left corner of the Search History table in the newer version of the user interface.*

*
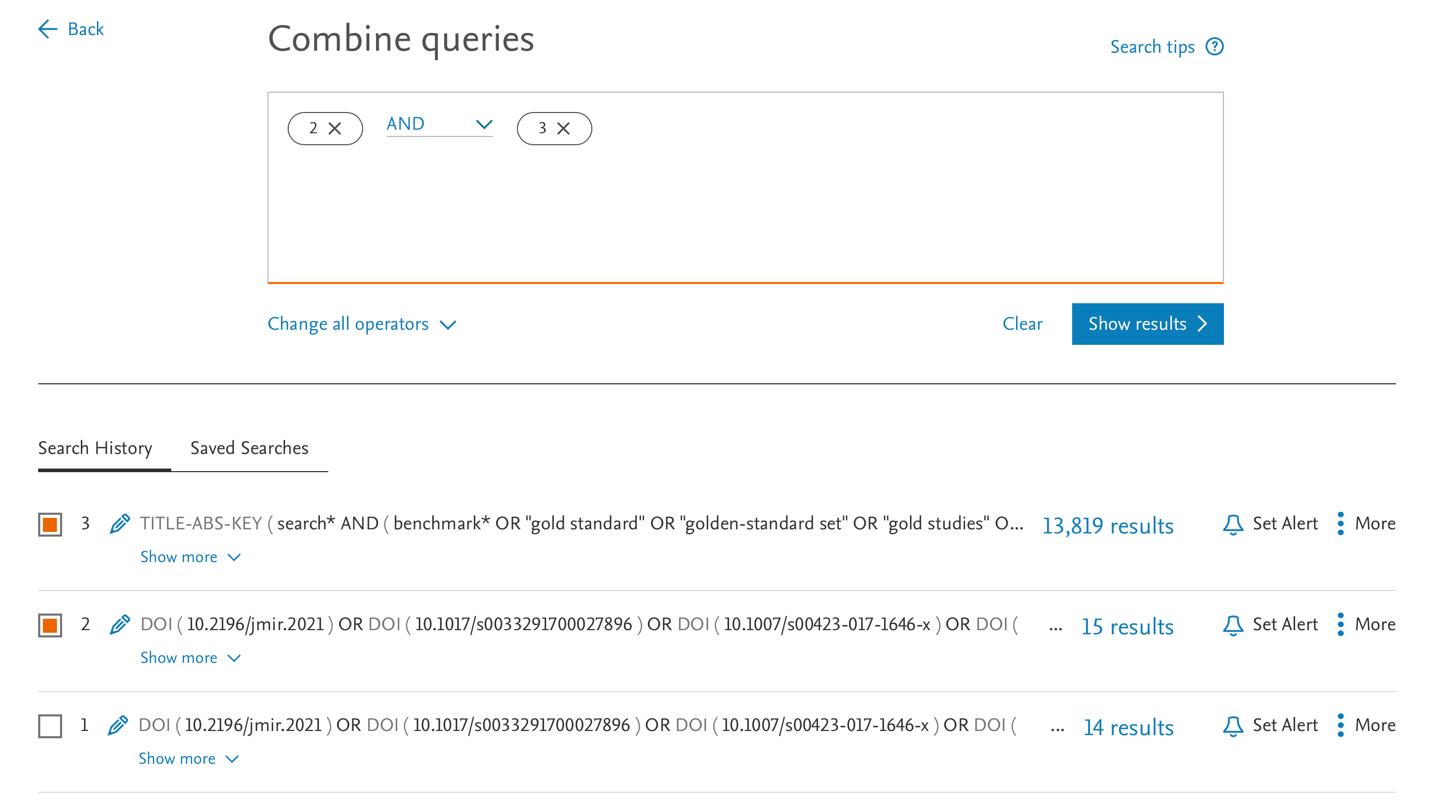
*

***Screenshot Scopus 7.***

*“Combine queries” window in the newer version of the interface. After selecting queries to combine (using tick-boxes) you can choose which Boolean operator to use between them.*

*
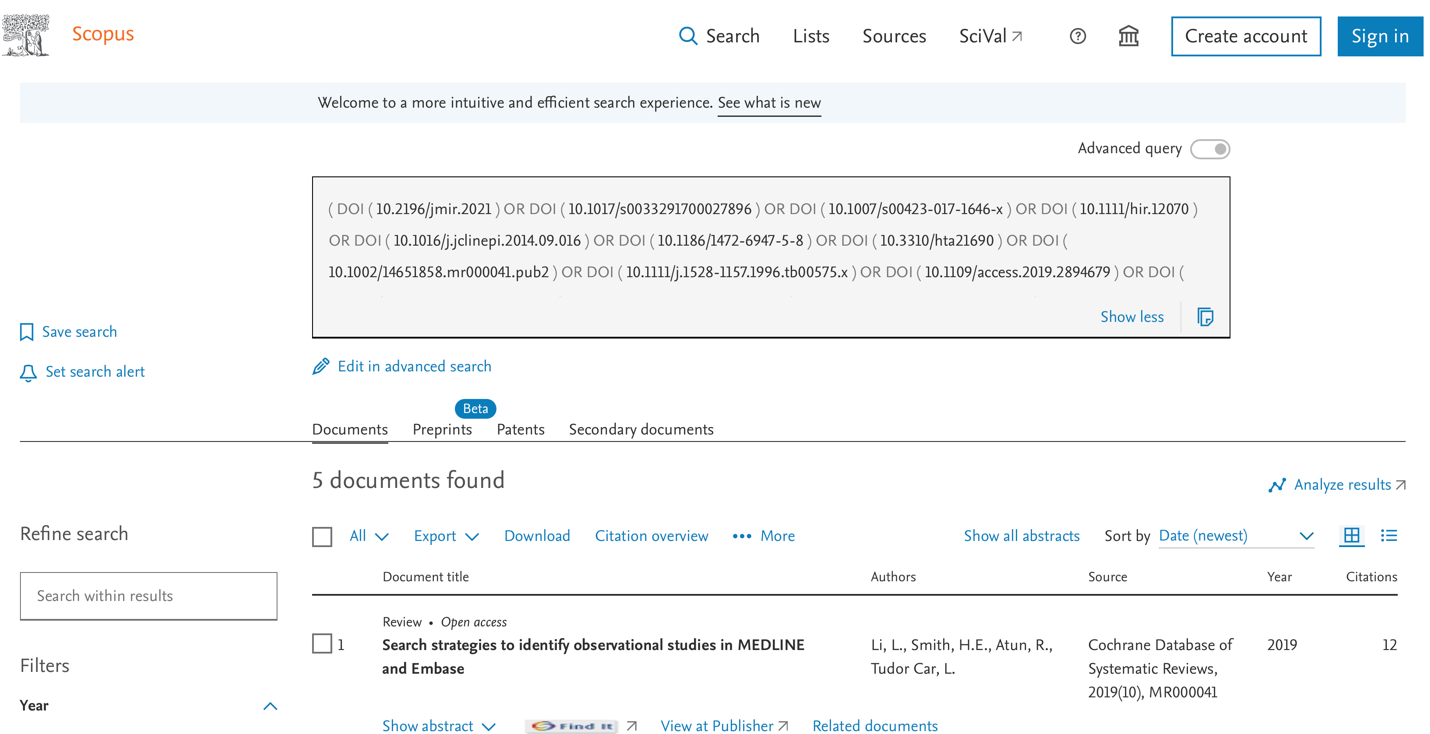
*

***Screenshot Scopus 8.***

*Example of results combining benchmarking and Target search strings using in the newer version of the interface.*

- 1. **Optional: If some benchmark records are missing, you can sometimes use "NOT" operator to see which one are missing (i.e. "(StringB) NOT (StringA)"):**

There is a “AND NOT” operator for combining search results (queries) manually by using their search numbers, e.g., enter: *#2 AND NOT #4*. After executing the search, this will return the lit of benchmarks which were not found by the target search string.

**
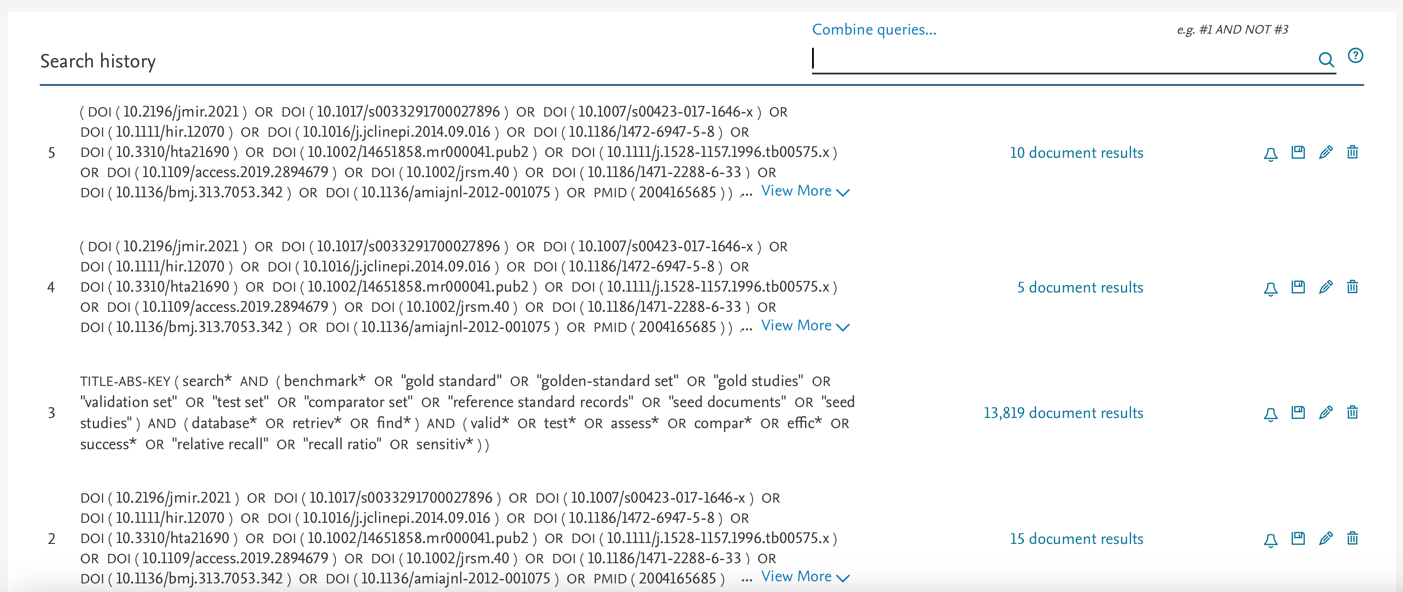
**

***Screenshot Scopus 6.***

*Results of combining two search queries using Boolean NOT operator are shown as search history record number 5. Using “AND NOT” Boolean operator reveals the benchmark records not found by the target search string.*

### Step6

1. **Calculate sensitivity of the target search string.**
   1. **The number of overlapping records between the two search strings (target and benchmarking) is the number of the benchmark studies found by the evaluated target string (StringA). Thus, this number, divided by the total number of records retrieved by benchmarking string (StringB) is the estimate of your search sensitivity (SEN or relative recall):**

Since the overlap is 4 records, the sensitivity is 5/15 = 0.33, or 33%.

- 1. **Optional: You can iteratively modify your target search string (StringA). At every iteration, it is very easy to re-evaluate new target StringA against benchmarking StringB using the same method as above (combining the strings). When modifying your search string, you can start by reading through the titles and abstracts of these missed studies. Determine why the study was missed by your current search strategy. What terms are missing from your search string? If reasonable, add the missing search terms to your search (e.g., add terms that are synonyms of concepts already included in your search, expand proximity windows, adjust stemming, etc.). If there is no reasonable way to adjust the search to capture the study, make a note of this as a potential limitation of your search strategy. You can also see which benchmark papers were found:**

The list of results appears automatically when running the search string from the Query box. You can also use the links in the search History, by clicking on the number of the records found by each search string.

- 1. **Recommended: Keep a good record of the search development and testing process (e.g., in a table), so you can document it transparently in your systematic review or protocol:**

Scopus does not allow exporting search history from the search window. You can also copy and paste Search history table for your record.

Search history manually saved in a table may look like this:

| **Date / database** | **Search nr** | **Search string** | **Search result / comment** |
| --- | --- | --- | --- |
| 10 January 2024  Scopus | 1 | *DOI ( 10.2196/jmir.2021 ) OR DOI ( 10.1017/s0033291700027896 ) OR DOI ( 10.1007/s00423-017-1646-x ) OR DOI ( 10.1111/hir.12070 ) OR DOI ( 10.1016/j.jclinepi.2014.09.016 ) OR DOI ( 10.1186/1472-6947-5-8 ) OR DOI ( 10.1111/j.1471-1842.2004.00511.x ) OR DOI ( 10.3310/hta21690 ) OR DOI ( 10.1002/14651858.mr000041.pub2 ) OR DOI ( 10.1111/j.1528-1157.1996.tb00575.x ) OR DOI ( 10.1109/access.2019.2894679 ) OR DOI ( 10.1002/jrsm.40 ) OR DOI ( 10.1186/1471-2288-6-33 ) OR DOI ( 10.1136/bmj.313.7053.342 ) OR DOI ( 10.1136/amiajnl-2012-001075 )* | 14 /  14 out of 15 benchmark studies found, 1 benchmark study not found |
|  | 2 | *DOI ( 10.2196/jmir.2021 ) OR DOI ( 10.1017/s0033291700027896 ) OR DOI ( 10.1007/s00423-017-1646-x ) OR DOI ( 10.1111/hir.12070 ) OR DOI ( 10.1016/j.jclinepi.2014.09.016 ) OR DOI ( 10.1186/1472-6947-5-8 ) OR DOI ( 10.3310/hta21690 ) OR DOI ( 10.1002/14651858.mr000041.pub2 ) OR DOI ( 10.1111/j.1528-1157.1996.tb00575.x ) OR DOI ( 10.1109/access.2019.2894679 ) OR DOI ( 10.1002/jrsm.40 ) OR DOI ( 10.1186/1471-2288-6-33 ) OR DOI ( 10.1136/bmj.313.7053.342 ) OR DOI ( 10.1136/amiajnl-2012-001075 ) OR PMID ( 2004165685 )* | 15 / Replaced missing benchmark by using its PMID and now 15/15 are found |
|  | 3 | *TITLE-ABS-KEY ( search* AND ( benchmark* OR "gold standard" OR "golden-standard set" OR "gold studies" OR "validation set" OR "test set" OR "comparator set" OR "reference standard records" OR "seed documents" OR "seed studies" ) AND ( database* OR retriev* OR find* ) AND ( valid* OR test* OR assess* OR compar* OR effic* OR success* OR "relative recall" OR "recall ratio" OR sensitiv* ) )* | 13,819 / Target search string |
|  | 4 | *#2 AND #3* | 5 / Sensitivity is 5/15 = 0.33 (33%). Refine target search string |
|  | 5 | *#2 AND NOT #4* | 10 / Missing benchmark papers |

## Combining benchmarking across multiple databases

### Databases and benchmarking searches

In this closing example, we assume that a separate benchmarking workflow was run, as described in the examples above) for each of the following five databases: OVID Embase, EBSCO Host, PubMed, Web of Science Core Collection, Scopus. Note that the same initial set of benchmarking studies was used in each example and a similar (but not identical) target search string was also used. Sensitivity of the target search string was calculated separately for each database.


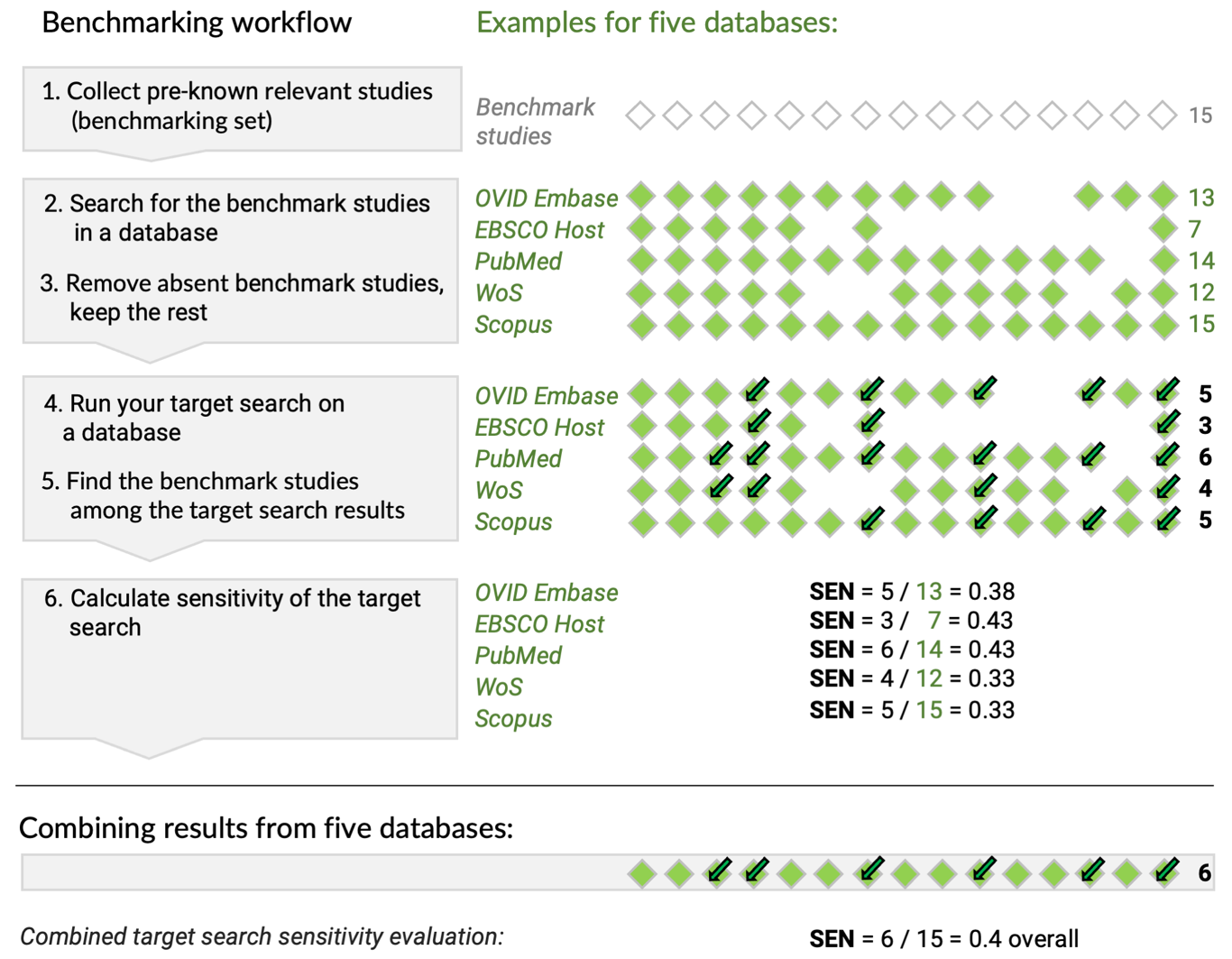


**Figure S2**

An example practical implementation of combining search sensitivity evaluations (benchmarking) across five example database searches.

### Combining benchmarking across databases

To estimate overall search sensitivity across all databases, we can aggregate results of benchmarking evaluations from each database (*Figure S2*).

The aim is to establish overall how many benchmarking papers were indexed in any of the databases and how many of the benchmarking papers were found in any of the databases. We can then use this information to calculate overall search sensitivity (SEN; relative recall).

In our example, across all databases all benchmarking papers were indexed, so the total number is 15. Target searches across all databases found only 6 of the benchmark studies (the number varied from 3 to 6). This gives the overall value of search sensitivity of 6 / 15 = 0.40.
